# The Potential Impact of Connexin 43 Expression on Bcl-2 Protein Level and Taxane Sensitivity in Head and Neck Cancers–In Vitro Studies

**DOI:** 10.3390/cancers11121848

**Published:** 2019-11-22

**Authors:** Bianka Gurbi, Diána Brauswetter, Attila Varga, Pál Gyulavári, Kinga Pénzes, József Murányi, Veronika Zámbó, Ede Birtalan, Tibor Krenács, David Laurence Becker, Miklós Csala, István Vályi-Nagy, István Peták, Kornél Dános

**Affiliations:** 1 Department of Medical Chemistry, Molecular Biology and Pathobiochemistry, Semmelweis University, H-1094 Budapest, Hungary; brauswetterdiana@gmail.com (D.B.); varga.attila@med.semmelweis-univ.hu (A.V.); gyulavari.pal@med.semmelweis-univ.hu (P.G.); kingaviktoria@yahoo.com (K.P.); jozsefmuranyi84@gmail.com (J.M.); zambo.veronika@med.semmelweis-univ.hu (V.Z.); csala.miklos@med.semmelweis-univ.hu (M.C.); 2 MTA-SE Pathobiochemistry Research Group, Semmelweis University, H-1094 Budapest, Hungary; istvan.petak@oncompassmedicine.com; 3 Department of Oto-Rhino-Laryngology, Head and Neck Surgery, Semmelweis University, H-1083 Budapest, Hungary; birtalanede@gmail.com (E.B.);; 4 Department of Pathology and Experimental Cancer Research, Semmelweis University, H-1085 Budapest, Hungary; 5 Lee Kong Chian School of Medicine, Nanyang Technological University, Singapore 308232, Singapore; david.becker@ntu.edu.sg; 6 Central Hospital of Southern Pest National Institute of Hematology and Infectious Diseases, H-1097 Budapest, Hungary; foigazgatosag@dpckorhaz.hu; 7 Oncompass Medicine Ltd, H-1024 Budapest, Hungary

**Keywords:** head and neck cancer, paclitaxel, connexin 43, Bcl-2

## Abstract

The poor prognosis of head and neck squamous cell carcinoma (HNSCC) is partly due to the lack of reliable predictive markers. Connexin 43 (Cx43) protein and its cell-communication channels have been assigned tumor suppressor functions while the anti-apoptotic Bcl-2 (B-cell lymphoma-2) protein has been associated with negative prognostic significance in cancer. This study aimed to test the role of Cx43 protein on Bcl-2 expression, tumor progression and response to taxane-based treatment in HNSCC. Human papillomavirus (HPV) negative HNSCC cell lines were tested for paclitaxel sensitivity through measuring apoptosis induction, cell viability and changes in Cx43 and Bcl-2 levels using flow cytometry, cell viability assay, immunocytochemistry and western blot. Inhibition of Cx43 expression using siRNA increased Bcl-2 protein levels in SCC25 (tongue squamous cell carcinoma) cells, while forced Cx43 expression reduced Bcl-2 levels and supported paclitaxel cytotoxicity in FaDu (hypopharynx squamous cell carcinoma) cells. In vitro results were in line with protein expression and clinicopathological features tested in tissue microarray samples of HNSCC patients. Our data demonstrate that elevated Cx43 and reduced Bcl-2 levels may indicate HNSCC sensitivity to taxane-based treatments. On the contrary, silencing of the Cx43 gene *GJA1* (gap junction protein alpha-1) can result in increased Bcl-2 expression and reduced paclitaxel efficiency. Clinical tumor-based analysis also confirmed the inverse correlation between Cx43 and Bcl-2 expression.

## 1. Introduction

An estimated 600,000 new head and neck cancers (tumors of the lip, oral cavity, larynx, nasopharynx, oropharynx and hypopharynx) are diagnosed annually worldwide [1]. The 5-year overall survival is only about 50%, which can be due to the fact that more than half of the tumors are diagnosed at advanced stages [2].

Modern, targeted therapy drugs—such as the Food and Drug Administration (FDA) approved cetuximab—shows clear benefit only for the treatment of recurrent or metastatic head and neck cancers as first line therapy compared to chemotherapy [3]. Certain immune checkpoint inhibitors (anti-PD-1 (programmed cell death protein 1), anti-PD-L1 (programmed death-ligand 1) and anti-CTLA-4 (cytotoxic T-lymphocyte-associated antigen 4) antibodies) demonstrated clinical benefit and were approved by FDA for patients with HNSCCs (head and neck squamous cell carcinoma) which showed progression after treatment with platinum-based chemotherapy. However, there is a lack of reliable predictive biomarkers for immunotherapy [4].

Currently, chemoradiotherapy (CRT) is still the most often used non-surgical therapeutic choice in locoregionally advanced HNSCC. However, no biomarker is available to predict the therapeutic response, resulting in a substantial number of patients who suffer the side effects of such a toxic treatment without clinical benefit. Bioradiotherapy with cetuximab for the same setting has recently been shown to be less effective than platinum-based CRT [5,6]. One of the therapeutic strategies for locally advanced HNSCC is induction chemotherapy containing docetaxel/paclitaxel+platinum+5-fluorouracil (TPF) [7]. Trials demonstrated superiority of the TPF induction regimen over platinum+5-fluorouracil (PF) in the management of locoregionally advanced HNSCC [8]. Paclitaxel is an antitumor agent that stabilizes microtubules and blocks cells in G2/M phase [9].

One of the few potential predictive biomarkers is ERCC1 (excision repair cross-complementing 1) protein. Findings show that ERCC1 expression might predict response to chemotherapeutics like 5-fluorouracil/cisplatin in HNSCCs [10]. Another candidate biomarker is connexin 43 (Cx43) protein. In vitro studies found a significant correlation between Cx43 expression and apoptosis inducing effect of paclitaxel. Cell lines with high levels of Cx43 protein were the most sensitive to taxane-based chemotherapy [11]. Paclitaxel therapy was also found to have the highest effect when high Cx43 expression was coupled with decreased expression of the specific antiapoptotic protein Bcl-2 (B-cell lymphoma 2) [12].

Connexins, such as Cx43 are proteins acting as structural elements in the formation of gap junctions. Gap junctions are responsible for intercellular communications allowing the transmission of ions or small molecules between cells [13]. Besides, Cx43 is also a tumor suppressor. The decreased expression of Cx43 correlates with tumor formation and poor prognosis in solid tumors such as breast cancer and HNSCCs [14,15].

Bcl-2 family proteins are important regulators of apoptosis. This family of interacting partners includes both inhibitors and inducers of cell death. Most Bcl-2 family proteins work on the membranes of mitochondria and endoplasmic reticulum facilitated by their hydrophobic membrane anchoring carboxyl-terminal domain. Bcl-2 protein increases the total cell number by preventing cell death rather than by increasing cell division rate [16].

The aim of this study was to evaluate the predictive value of Cx43 and Bcl-2 expression and their coexpression in HNSCCs when using paclitaxel chemotherapy.

## 2. Results

### 2.1. Expression of Cx43 and Bcl-2 in Head and Neck Cancer Cell Lines

**Figure 1:**
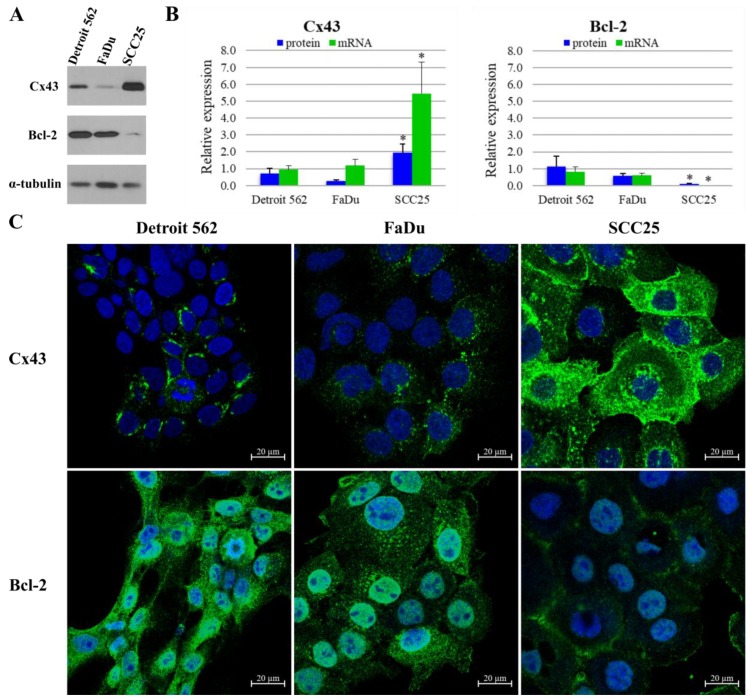
Expression of connexin 43 (Cx43) and B-cell lymphoma-2 (Bcl-2) in head and neck squamous cell carcinoma (HNSCC) cell lines. (**A**) Cells were subjected to western blot analysis with antibodies against Cx43, Bcl-2 and the loading control, α-tubulin. (**B**) Densitometry analysis of Cx43 and Bcl-2 protein expression in Detroit 562 (metastatic pharyngeal carcinoma), FaDu (hypopharynx squamous cell carcinoma) and SCC25 (tongue squamous cell carcinoma) cells. Quantitative PCR (qPCR) analysis of Cx43 and Bcl-2 mRNA expression in HNSCC cell lines. Densitometry analysis and qPCR analysis show the results of three independent experiments. The expressions of all proteins and mRNAs were normalized to the expression of α-tubulin. Data are presented as mean ± SD (standard deviation). Statistical analysis was performed by Student’s *t*-test, the Cx43 and Bcl-2 expression of the cell lines were compared to each other. * *p* < 0.05 (**C**) Representative immunofluorescence images of Cx43 and Bcl-2 expression in Detroit 562, FaDu and SCC25 cell lines. Cx43 and Bcl-2 were marked with Alexa Fluor 488 (green), nuclei were stained with DRAQ5 (blue).

Cx43 and Bcl-2 protein expression and localization were tested using western blot and immunocytochemistry in HNSCC cell lines (Figure 1). According to western blot analysis, Detroit 562 (metastatic pharyngeal carcinoma) cells express Cx43 at a medium level compared to other two cell lines. FaDu cells express approximately half (*p* = 0.05), whereas SCC25 (tongue squamous cell carcinoma) cells more than twice (*p* = 0.02) as much Cx43 than Detroit 562 cells. Expression of Cx43 is low in Detroit 562 and FaDu (hypopharynx squamous cell carcinoma), as opposed to SCC25 with high protein expression. Regarding Bcl-2 protein, Detroit 562 cells show the highest expression level with FaDu cells being close second (*p* = 0.22). In contrast, the SCC25 cells harbor very low amounts of Bcl-2 protein, about one tenth of what Detroit 562 cells express (*p* = 0.05). Expression of Bcl-2 is high in Detroit 562 and FaDu, as opposed to SCC25 with low protein expression. (Figure 1A,B) These western blot results are in line with the data acquired by immunofluorescence imaging. Cx43 protein was found to be localized in the nucleus, the cytoplasm and the plasma membrane of SCC25 cells. Cx43 was also detected in the cytoplasm of Detroit 562 and FaDu cells. Bcl-2 protein was present in the nucleus and the cytoplasm of all three cell lines (Figure 1C).

As tested with quantitative real-time PCR (qPCR), Cx43 and Bcl-2 mRNA expression pattern was in line with the protein levels measured with western blot. FaDu cells produced similar amount (*p* = 0.40), whereas SCC25 cells expressed five times more Cx43 mRNA than Detroit 562 cells (*p* = 0.02). Bcl-2 mRNA levels were not significantly different between FaDu and Detroit 562 cells (*p* = 0.26), while SCC25 cells produced only negligible amount (*p* = 8e−04) of Bcl-2 compared to Detroit 562 cells (Figure 1B). The published mRNA levels of these cell lines from Cancer Cell Line Encyclopedia are consistent with our results [17].

### 2.2. Effect of Paclitaxel on the Viability of Head and Neck Cancer Cell Lines

**Figure 2:**
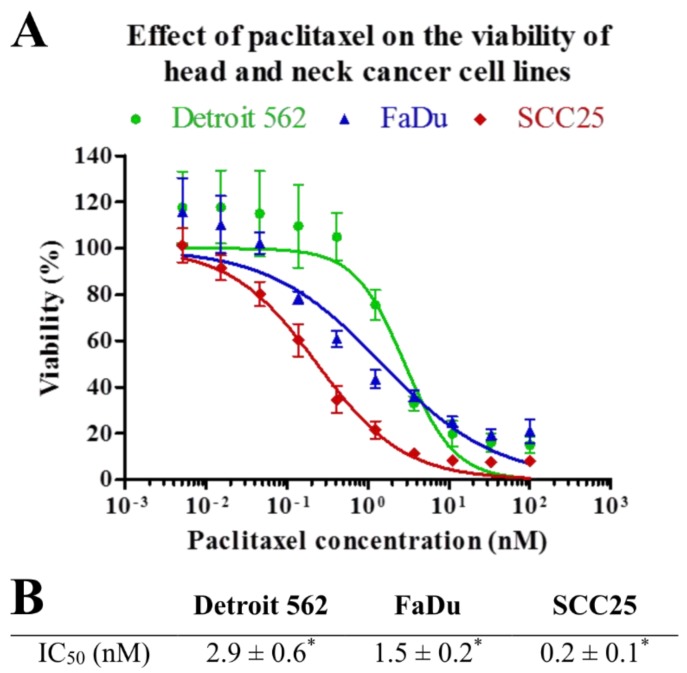
Effect of paclitaxel on cell viability. HNSCC cell lines were analyzed in parallel by MTT after 72 h of treatment with paclitaxel at different concentrations. (**A**) IC_50_ curves of paclitaxel on Detroit 562, FaDu and SCC25 cell lines. The results represent the mean of three independent experiments with SD. (**B**) IC_50_ concentrations of paclitaxel measured in Detroit 562, FaDu and SCC25 cell lines. IC_50_ values are the mean of three different measurements ± SD. Statistical analysis was performed by Student’s *t*-test, the IC_50_ concentrations of the cell lines were compared to each other. * *p* < 0.05.

Viability of HNSCC cells was analyzed in parallel experiments by using MTT after 72 h of treatment with paclitaxel at different concentrations. Paclitaxel effectively decreased viability of all three cell lines. However, SCC25 showed a significantly higher sensitivity to paclitaxel than the other two cell lines (*p* = 0.002 and *p* = 5e−04). Detroit 562 and FaDu cell lines displayed moderate sensitivity to paclitaxel. There is a slight, but statistically significant (*p* = 0.02) difference between the IC_50_ value of Detroit 562 and FaDu cell lines (Figure 2).

### 2.3. Paclitaxel-Induced Apoptosis of Head and Neck Cancer Cell Lines

**Figure 3:**
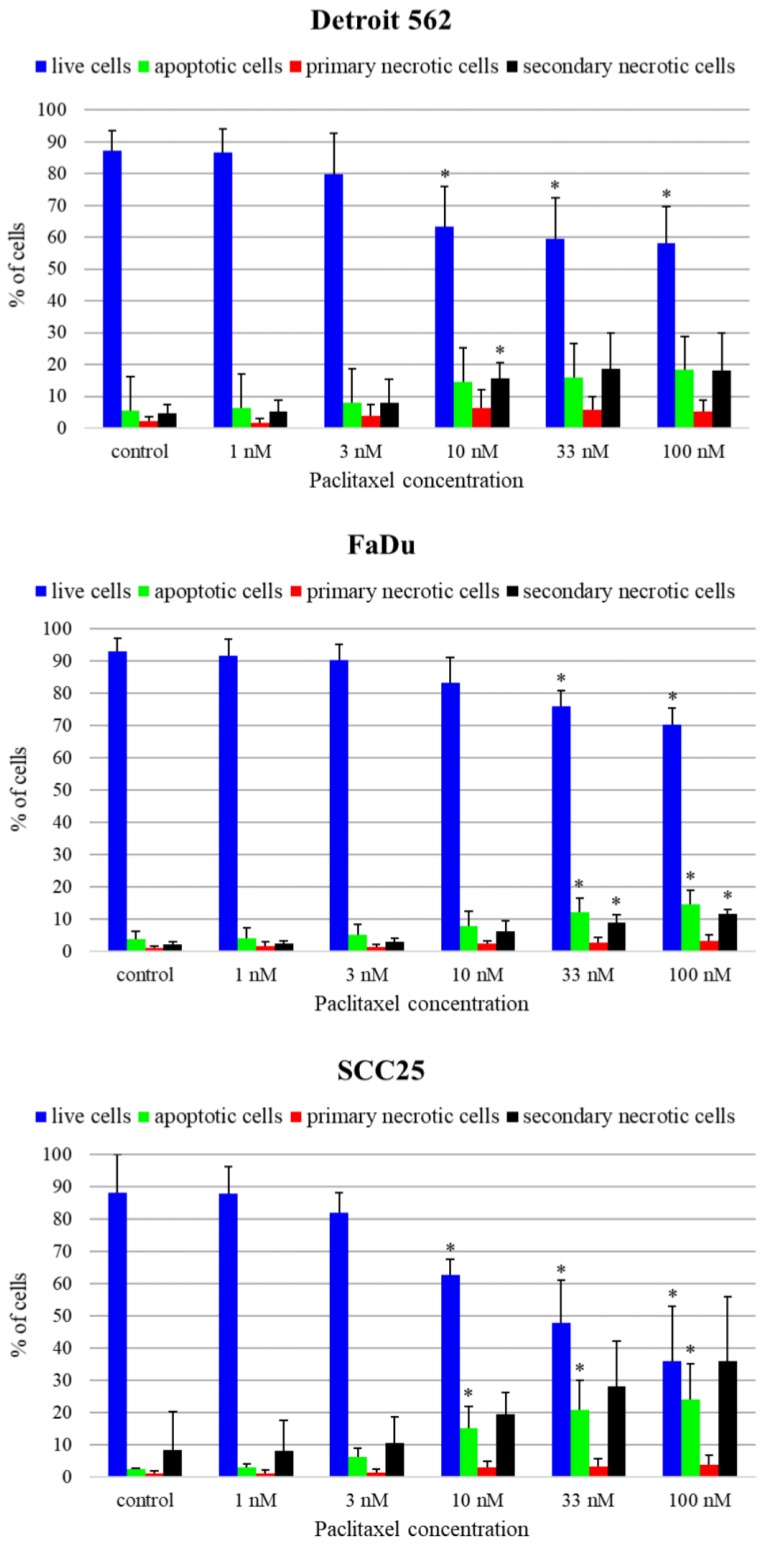
Paclitaxel-induced apoptosis of Detroit 562, FaDu and SCC25 cells. Annexin V-FLUOS/ propidium iodide (Ann/PI)-stained HNSCC cells were analyzed by FACS (fluorescence-activated cell sorting) after 48 h of treatment with paclitaxel different concentrations. Live cells are presented by the Ann-/PI- fraction, apoptotic cells by the Ann+/PI- fraction, secondary necrotic cells by the Ann+/PI+ fraction and primary necrotic cells are detected in the Ann-/PI+ fraction. Data are presented as mean ± SD. Statistical analysis was performed by Student’s *t*-test, the cell fractions at all concentrations were compared to control fractions in each cell line * *p* < 0.05.

To reveal the mechanism of cell death caused by paclitaxel, we performed flow cytometry experiments using Annexin V-FLUOS and PI (propidium iodide) double staining at one treatment time (48 h) and five paclitaxel concentrations (1 nM, 3 nM, 10 nM, 33 nM, 100 nM). According to annexin and PI positivity, four populations could be discerned: live cells, cells undergoing apoptosis, primary necrotic and secondary necrotic cells. In Detroit 562 cell line, only a little increase of the apoptosis rate could be observed. The proportion of cells undergoing apoptosis rose slightly with higher paclitaxel concentrations compared to the control sample. This increase was not significant (*p* > 0.05). We could observe only a slight increase in the apoptosis rate in FaDu cell line. The proportion of cells undergoing apoptosis rose with higher paclitaxel concentrations: there was a significant rise compared to control at 33 nM (*p* = 0.045) and 100 nM (*p* = 0.022) paclitaxel concentrations. In SCC25 cell line, a high increase in apoptosis rate could be observed. The proportion of cells undergoing apoptosis rose with higher paclitaxel concentrations. Significant differences could be seen between the control and the paclitaxel treated samples even at 10 nM concentration (10 nM *p* = 0.033, 33 nM *p* = 0.025, 100 nM *p* = 0.027). SCC25 showed a higher sensitivity to paclitaxel compared to the other two cell lines (Figure 3).

### 2.4. Effects of GJA1 siRNA and Cx43 Plasmid Transfection on Protein Expression of Head and Neck Cancer Cell Lines

**Figure 4:**
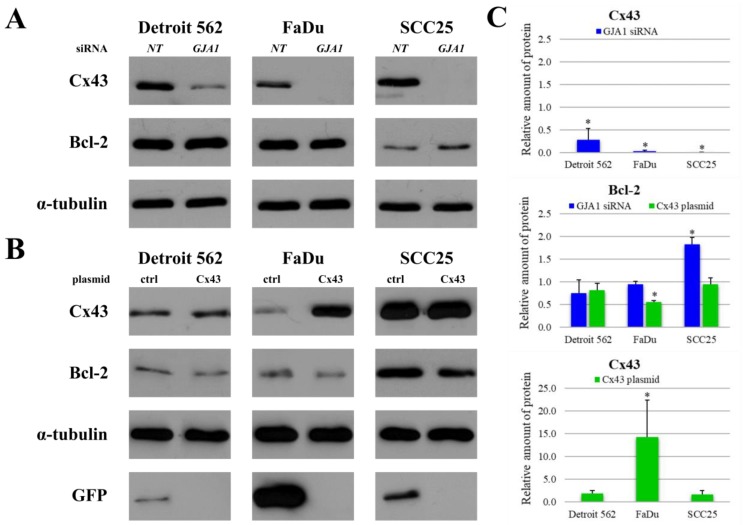
Changes in the levels of Cx43 and Bcl-2 after *GJA1* (gap junction protein alpha-1) siRNA knockdown and Cx43 plasmid transfection in HNSCC cell lines. (**A**) Cx43 knockdown cells were subjected to western blot analysis with antibodies against Cx43, Bcl-2 and the loading control, α-tubulin. NT: non-targeting (**B**) Cx43 transfected cells were subjected to western blot analysis with antibodies against Cx43, Bcl-2, GFP (green fluorescent protein) and the loading control, α-tubulin. GFP was used to confirm transfection success. ctrl: control (**C**) Densitometry analysis of Cx43 and Bcl-2 protein expression after *GJA1* siRNA knockdown and Cx43 plasmid transfection in Detroit 562, FaDu and SCC25 cells. Densitometry analysis was performed using three independent experiments. The expressions of all proteins were compared to those in the non-targeting siRNA treated negative controls or in the control plasmid treated controls, after normalization to α-tubulin. Data are presented as mean ± SD. Statistical analysis was performed using Student’s t-test, in each cell line the expression of all proteins in *GJA1* siRNA treated samples were compared to protein expression in non-targeting siRNA treated samples or in Cx43 plasmid treated samples were compared to protein expression in control plasmid treated samples. * *p* < 0.05.

In order to investigate whether Cx43 has any impact on the level of Bcl-2 protein, Cx43 gene (*GJA1,* gap junction protein alpha-1) silencing and plasmid transfection were performed on HNSCC cell lines. After *GJA1* siRNA knockdown the Cx43 protein expression was significantly reduced in all three cell lines (Detroit 562 *p* = 0.01, FaDu *p* = 3e−07, SCC25 *p* = e−11). This decrease had no effect on Bcl-2 expression in Detroit 562 (*p* = 0.22) and FaDu (*p* = 0.21) cells. However, knockdown of Cx43 in SCC25 resulted in a significant increase in Bcl-2 expression (*p* = 6e−04). The amount of Bcl-2 protein almost doubled. (Figure 4A,C) Plasmid transfection upregulated Cx43 protein levels in all three cell lines, but the difference was only significant in FaDu cells (*p* < 0.05) but not in Detroit 562 (*p* = 0.07), and SCC25 (*p* = 0.22) cells. Forced Cx43 expression reduced Bcl-2 protein level in each cell line, which was highly significant in FaDu (*p* = 8e−06) cells (Figure 4B,C) but not in Detroit 562 (*p* = 0.10) and SCC25 (*p* = 0.57) cells.

### 2.5. Effects of GJA1 siRNA on the Paclitaxel-Induced Apoptosis of SCC25 Cell Line

**Figure 5:**
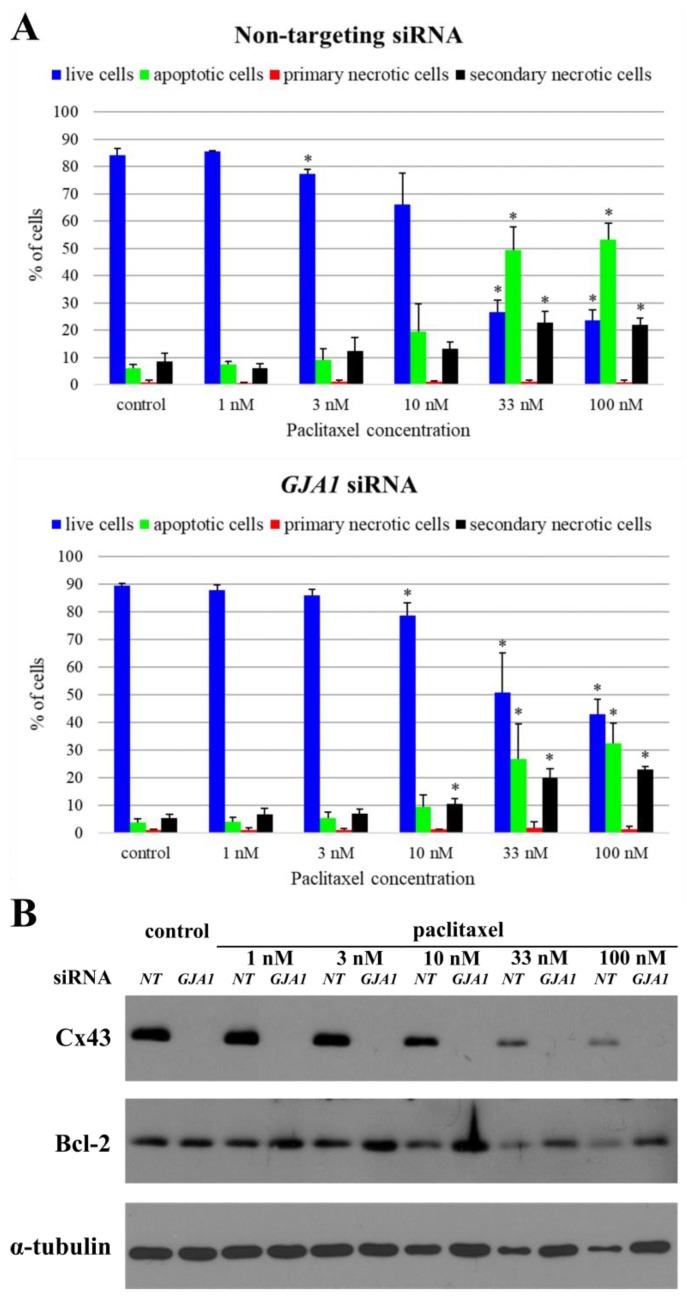
Changes in the paclitaxel-induced apoptosis of SCC25 after knocking down Cx43. (**A**) Annexin V-FLUOS/ propidium iodide (Ann/PI)-stained HNSCC cells were analyzed by FACS after 48 h of treatment with paclitaxel at different concentrations. Live cells are presented by the Ann-/PI- fraction, apoptotic cells by the Ann+/PI- fraction, secondary necrotic cells by the Ann+/PI+ fraction and primary necrotic cells are detected in the Ann-/PI+ fraction. Data are presented as mean ± SD. Statistical analysis was performed by Student’s *t*-test, the cell fractions in all concentration were compared to negative control fractions in non-targeting siRNA or *GJA1* siRNA treated samples. The cell fractions in *GJA1* siRNA treated samples were also compared to cell fractions in non-targeting siRNA treated samples. * *p* < 0.05. (**B**) Cell lysates were subjected to western blot analysis with antibodies against Cx43, Bcl-2 and the loading control, α-tubulin. NT: non-targeting.

Since *GJA1* siRNA had a significant effect on Bcl-2 protein expression in SCC25 cells, the possible impact of this siRNA on paclitaxel-induced apoptosis was also investigated. In *GJA1* siRNA treated samples, the proportion of apoptotic cells in all paclitaxel concentrations decreased compared to the negative control siRNA treated samples. This decrease was significant in 100 nM paclitaxel treated samples (*p* = 0.02). The Cx43 protein expression was reduced in all *GJA1* siRNA treated samples. Knockdown of Cx43 resulted in an increase in Bcl-2 expression. Thus, *GJA1* gene silencing explicitly reduced the effect of paclitaxel in SCC25 cell line (Figure 5).

To strengthen our assumption, paclitaxel-induced apoptosis was also analyzed in FaDu and Detroit 562 cell lines after applying *GJA1* siRNA or non-targeting siRNA (Figures S1 and S2). We found decreased paclitaxel sensitivity in the *GJA1* siRNA treated FaDu cell line, which has low Cx43 but also lower Bcl-2 expression than Detroit 562 cells. In case of Detroit 562, we did not observe remarkable changes in paclitaxel sensitivity when we used *GJA1* siRNA and non-targeting siRNA. The difference between the sensitivity of the two cell lines with low Cx43 protein expression can be explained with their different Bcl-2 protein expressions. Comparing with the other two cell lines, Detroit 562 cells had low Cx43 protein expression, but the highest Bcl-2 protein level. The elevated level of Bcl-2 protein can decrease the basal paclitaxel sensitivity of Detroit 562 cells. This could explain the lack of change in paclitaxel sensitivity of Detroit 562 cells after silencing *GJA1* gene.

Parallel with the analysis of cell apoptosis, cell proliferation was also examined in the same samples of Detroit 562, FaDu and SCC25 cell lines with trypan blue exclusion test. The obtained results confirmed our previous data, as the application of *GJA1* siRNA decreased the effect of paclitaxel in both SCC25 and FaDu cell lines (in particular in SCC25 cell line) but did not alter the paclitaxel sensitivity of Detroit 562 cells (Figure S3, Table S1).

### 2.6. Effects of Cx43 Plasmid Transfection on the Paclitaxel-Induced Cell Death of FaDu Cell Line

**Figure 6:**
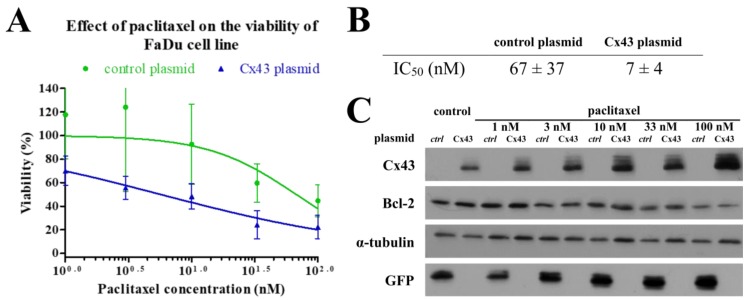
Changes in the effect of paclitaxel on cell viability of FaDu after transfection Cx43. FaDu cells were analyzed by trypan blue exclusion test after 48 h of treatment with paclitaxel at different concentrations. (**A**) IC_50_ curves of paclitaxel on FaDu cell line. The results represent the mean of three independent experiments with SD. (**B**) IC_50_ concentrations of paclitaxel measured in FaDu cell line. IC_50_ values are the mean of three different measurements ± SD. Statistical analysis was performed by Student’s *t*-test, the IC_50_ concentrations were compared to each other. *p < 0.05 (**C**) Cell lysates were subjected to western blot analysis with antibodies against Cx43, Bcl-2, GFP and the loading control, α-tubulin. GFP was used to confirm transfection success. ctrl: control.

Since forced Cx43 expression had a significant effect on Bcl-2 expression in FaDu cell line we investigated how this can influence paclitaxel sensitivity. We analyzed the cell proliferation and apoptosis using the same methods as for the *GJA1* siRNA experiments. Although the flow cytometry analysis showed a non-significant effect, the results of the trypan blue exclusion test revealed that the Cx43 transfection can sensitizes FaDu cells to paclitaxel treatment. Cx43 plasmid transfected FaDu cells had lower paclitaxel IC_50_ values (Figure 6).

### 2.7. Cx43 and Bcl-2 Expression in Patient-Derived HNSCC Samples

High Cx43 expression (Cx43^H^) was found in 44/58 (75.9%) of HNSCCs. Cx43 status correlated with disease-specific survival (DSS, *p* = 0.024), the reduction of Cx43 expression level was associated with a better prognosis. The Cx43 status did not correlate with tumor localization (*p* = 0.779), tumor size (*p* = 0.824), stage (*p* = 0.638), lymph node metastasis (*p* = 0.351) or response to neoadjuvant chemotherapy (*p* > 0.05).

Of the 58 tumor samples 9 (15.5%) cases showed high Bcl-2 expression (Bcl-2^H^). The Bcl-2 status did not correlate with DSS (*p* = 0.21), tumor localization (*p* = 0.531), tumor size (*p* = 0.136), stage (*p*= 0.748), lymph node metastasis (*p* = 0.111) or response to neoadjuvant chemotherapy (*p* = 0.544). However, the reduction in Bcl-2 expression was associated with poorer prognosis in oropharyngeal samples, this phenomenon was not significant (*p* = 0.056). It has been observed that lymph node metastasis occurs more frequently in Bcl-2^H^ cases (of the 9 Bcl-2^H^ tumor samples, 7 proved to bear lymph node metastasis).

**Figure 7:**
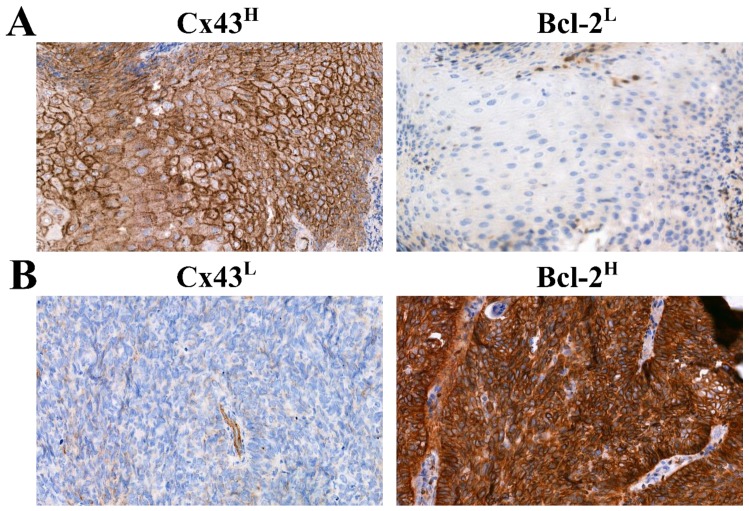
Immunohistochemical analysis of Bcl-2 and Cx43 expression (**A**) Cx43^H^/Bcl-2^L^ (**B**) Cx43^L^/Bcl-2^H^) in HNSCC tissue samples. (Magnification: 40×).

**Figure 8:**
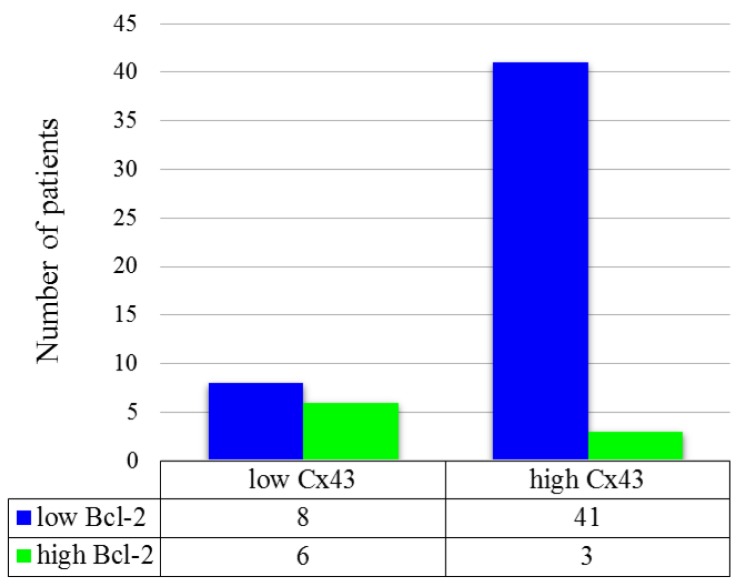
Distribution of patients according to Cx43 and Bcl-2 immunohistochemistry. Groups were compared by Fisher’s exact test (*p* = 0.013).

Correlation between Cx43 and Bcl-2 protein expression was also investigated. Low Cx43 expression (Cx43^L^) / low Bcl-2 expression (Bcl-2^L^) cases were found to be 13.8% (8/58), Cx43^H^/Bcl-2^L^ 70.7% (41/58), Cx43^L^/Bcl-2^H^ 10.3% (6/58), Cx43^H^/Bcl-2^H^ 5.2% (3/58). Typical Cx43^H^/Bcl-2^L^ (A) and Cx43^L^/Bcl-2^H^ (B) tissue samples are depicted in Figure 7. Low Cx43 expression was associated with high Bcl-2 expression (*p* = 0.013). This finding implies that Cx43 negative tumors overexpress Bcl-2 more often than Cx43 positive ones. (Figure 8) The combined status of Cx43 and Bcl-2 did not correlate with DSS (*p* > 0.05), tumor localization (*p* = 0.904), tumor size (*p* = 0.687), stage (*p* = 0.924), lymph node metastasis (*p* = 0.43) or response to neoadjuvant chemotherapy (*p* > 0.05).

## 3. Discussion

Taxanes, especially docetaxel, are used in combination with cisplatin and 5-fluorouracil as a neoadjuvant chemotherapy in the treatment of HNSCC patients. In this study, Cx43 and Bcl-2 expression as a potential predictive molecular marker of taxane drug sensitivity was investigated in HNSCC cell lines and tumor cells.

Bcl-2 family proteins are key regulators of apoptosis. Bcl-2 increases the total cell number by preventing cell death rather than by increasing cell division rate [16]. HNSCCs often overexpress the anti-apoptotic Bcl-2 protein, which proved to be associated with chemoresistance [18]. Bcl-2 is also a possible therapeutic target in combination with chemotherapy [19].

Connexins are well-characterized gap junction proteins, however, previous studies revealed their possible tumor suppressing role and interference with intracellular signaling pathways, for example, the Src-related pathway [20]. Connexins were shown to regulate tissue homeostasis through coordinating cellular events, such as cell growth, migration, apoptosis or signaling. These roles are independent from their gap junctional communication, although their exact mechanism is waiting for clarification [21,22]. Loss of function mutations of these gap junction channels have been described in many disorders including various cancers [23].

In our previous study, we found that Cx43 could be regarded as a prognostic factor in head and neck cancers, and the reduction of its expression level is associated with a significantly poorer prognosis [14]. This was confirmed by Puzzo et al. in laryngeal cancers [24]. However, other studies found inverse association between Cx43 expression and overall survival suggesting, that Cx43 has different role in certain tumor types (esophageal, oropharyngeal) or localization (cytoplasm and nucleus vs. membrane) [25,26,27]. Dubina et al. also found frame-shift mutations at the carboxyl-terminal region of Cx43 in human colon adenocarcinomas, which affects its phosphorylation, localization and function of the protein and also its staining with different antibodies [28].

The potential of Cx43 as a predictive marker for chemotherapy in head and neck cancers has not been reported in the literature. However, it has been observed that transfection with Cx43 decreased cell growth in several cancer cell lines such as lung, breast or prostate [29,30,31].

We employed three head and neck cancer cell lines of different localization (Detroit562 pharynx, FaDu hypopharynx and SCC25 tongue) to create an in vitro model of head and neck cancers. We used western blot and fluorescent microscopy to investigate protein level and localization of Cx43 and Bcl-2 in each cell line and immunohistochemistry for the same reason in formalin-fixed, paraffin-embedded (FFPE) tumor samples. We found that the investigated cell lines and tissue samples have different Cx43 and Bcl-2 expression. No significant association was found between Bcl-2 or Cx43 expression and patients clinicopathological data, such as stage, grade, localization or disease specific survival. However, interestingly, most of the patients with high Cx43 expression had worse outcome than those with low Cx43 expression. Although due to the low number of patients, these data have to be managed carefully, our result is consistent with the previous founding of Brockmeyer et al. [25].

Considering the relationship between Cx43 and Bcl-2 protein levels, it seems that high level of Cx43 is usually associated with weak expression of Bcl-2. The best example of this phenomenon is SCC25 cell line with the highest Cx43 and lowest Bcl-2 level which is the most sensitive to paclitaxel at the lowest IC_50_ value and the highest fraction of apoptotic cells. At the same time, low level of Cx43 was associated with increased Bcl-2 protein level and relatively lower paclitaxel sensitivity in the remaining two cell lines. The observed phenomenon, that elevated basal Bcl-2 protein level can decrease paclitaxel sensitivity despite higher Cx43 protein as seen in Detroit 562 cell line, can highlight the importance of Bcl-2 protein besides Cx43. Our results are in line with previous findings in glioblastoma cell lines where high expression level of Cx43 was accompanied by low Bcl-2 expression and high sensitivity to therapies containing taxane agents [12]. Similar effects were observed in transfected ovarian [32] and prostate cancer cell lines [33] in vitro and in mouse models. Huang et al. hypothesized that this mechanism could be independent of the gap junctional intercellular communication of Cx43, because in their cell model, Cx43 was localized mainly in the cytoplasm and the nucleus. They analyzed several apoptosis related genes (Bcl-2, Bax-1, Bac-1, Mcl-1) and found significant changes only in the protein level of Bcl-2 [34]. Zhang et al. observed that the enforced expression of Cx43 increased the protein level of the CDK inhibitor p27 in the osteosarcoma U2OS cells, they found no significant changes in the protein level of cyclin A, D1, E, CDK2, CDK4, CDK6, p15, p18 or p21 [35]. The exact mechanism by which Cx43 regulates gene expression is complex and still remains to be elucidated. In the SCC25 cell line, Cx43 is also localized in the nucleus, like the transfected Cx43 in the experiments of Huang et al. They considered that Cx43 directly binds to cis elements in the promoter region and regulates gene expression [12]. Another possible mechanism could affect through signal transduction, because Cx43 exhibits SH2 and SH3 binding sites, and inhibits the activity of c-Src. Wang et al. observed that the upregulation of Cx43 expression only sensitized colorectal cancer cells to paclitaxel when they were cultured at high density and cells were in contact with each other. This may indicate the importance of Cx43 gap junctional function in the mechanism of chemosensitization [11]. Cx43 binds to tubulin and is also involved in the stabilization of microtubules which can also have an impact on the effect of paclitaxel. Nevertheless, in our research we did not aim to investigate the direct interaction between Cx43 and microtubules [36].

In our study using tumor cell lines, we found an inverse association between Cx43 and Bcl-2 protein expression in the FFPE HNSCC tissue samples. High protein expression of Cx43 and low expression of Bcl-2 occurred frequently together which phenomenon proved to be significant. No significant correlation was verified between the effect of neoadjuvant TPF chemotherapy and the protein level of Cx43 or Bcl-2 but the low number of cases have to be considered (only 18 patients received TPF therapy). To verify the revealed inverse association between protein expression of Cx43 and Bcl-2 we used the mRNA expression data of head and neck tumors from TCGA (The Cancer Genome Atlas Program.) The database GEPIA (Gene Expression Profiling Interactive Analysis) allows us to perform pairwise gene correlation analysis of TCGA expression data. We analyzed the gene expression of *GJA1* and *BCL-2* in HNSCCs and found a significant inverse correlation (*p* = 2.4e−06, R = −0.21), which can confirm our result [37]. We could not isolate mRNA of appropriate quality from our FFPE HNSCC tissue samples.

In the present work, we used RNA interference and plasmid transfection to demonstrate the effect of Cx43 protein level on Bcl-2 expression. We found that significant reduction of Cx43 protein resulted a significantly elevated Bcl-2 protein expression in SCC25 cell line, whereas in FaDu cell line significant elevation of Cx43 protein resulted in a significantly decreased Bcl-2 protein expression. In case of RNA interference, we did not reveal any significant change in Bcl-2 protein level of FaDu and Detroit 562 which might be due to their low initial Cx43 protein expression and to the consequently small change in Cx43 protein levels in response to RNA interference. Huang et al. [12] found similar effects when transfecting Cx43 into glioblastoma cell lines. The decreased Cx43 expression in SCC25 cell lines lead to a lower paclitaxel sensitivity, decreased fraction of apoptotic cells while the increased Cx43 expression lead to a higher paclitaxel sensitivity in FaDu cell line. *GJA1* gene transfection resulted in increased Cx43 protein expression in all three cell lines, but the difference was significant only in FaDu cells. Their use for further experiments was also supported by their most efficient and best tolerance for transfection. In transfected Detroit 562 cells, elevated Cx43 expression decreased or almost disappeared after two days. The lack of significant change in Bcl-2 protein level in SCC25 cells might be due to the high initial Cx43 protein levels and which showed only minor increase in response to plasmid transfection. Though *GJA1* transfection did not induce significant apoptosis, it could sensitize FaDu cells to paclitaxel treatment transfection by itself cased some cell death, and since it was more efficient with the smaller control plasmid compared to the construct this led to a non-significant difference in cell death between these experiments. This disturbing effect did not change much when the transfection agents were reduced.

In summary, these data suggest that the expression of Cx43 and Bcl-2 are inversely correlated in head and neck cancer cell lines and tissue samples as well. High level of Cx43 and low level of Bcl-2 predicted a good response to paclitaxel treatment in the investigated cell lines. The specific role of Cx43 in this phenomenon was proved by using RNA interference and *GJA1* gene transfection.

## 4. Materials and Methods 

### 4.1. Cell Culturing and Inhibitors

Head and neck squamous cell carcinoma cell lines Detroit 562 (CCL-138™), FaDu (HTB-43™) and SCC25 (CRL-1628™) were obtained from American Type Culture Collection (ATCC). Detroit 562 cells were cultured in EMEM (Lonza) supplemented with 10% (V/V) fetal bovine serum (FBS, GIBCO), 0.1% (V/V) sodium pyruvate (Lonza) and 1% (V/V) antibiotic mix (MycoZap Plus-CL, Lonza). FaDu cells were maintained DMEM (Lonza) supplemented with 10% (V/V) fetal bovine serum (FBS, GIBCO), 0.1% added sodium pyruvate (Lonza) and 1% antibiotic mix (MycoZap Plus-CL, Lonza). SCC25 cells were cultured DMEM:F12 (Lonza) supplemented with 10% (V/V) fetal bovine serum (FBS, GIBCO), 400 ng/mL hydrocortisone (STEMCELL) and 1% antibiotic mix (MycoZap Plus-CL, Lonza) respectively in humidified atmosphere at 37 °C and 5% CO_2_. Cells were checked for mycoplasma (MycoAlert™ PLUS Mycoplasma Detection Kit, Cat. No. LT07-705, Lonza, Basel, Switzerland). Paclitaxel (Cat. No. S1150) was purchased from Selleckchem (Houston, TX, USA).

### 4.2. Cell Viability Assay

Cell viability assay was carried out as mentioned previously [38]. Briefly, HNSCC cells were seeded into 96 well plates at a density of 4 × 10^3^ cells/well. 

Cell lines were left overnight to attach, then treated with decreasing concentrations of paclitaxel in duplicates. Following this, 72 h after treatment, medium was removed and 50 μL PBS containing 1 mg/mL 3-(4,5-dimethylthiaziazol-2-yl)-2,5-diphenyl-2H-tetrazolium bromide (MTT) was added to each well and cells were incubated for 1 h at 37 °C. After the incubation MTT solution was removed and tetrazolium crystals were dissolved in isopropanol containing 10% (V/V) Triton X-100 and 1% (V/V) 0.1 N HCl. Absorbance was measured at 570 nm and 690 nm with a Synergy multimode reader (BioTek, Budapest, Hungary). The 690 nm data was subtracted from the 570 nm for each well. Absolute IC_50_ values were calculated by non-linear regression using Graph Pad Prism 5 software (GraphPad Software, San Diego, CA, USA). Each experiment was repeated at least three times.

### 4.3. Trypan Blue Exclusion Test

The proliferation of GJA1 siRNA + paclitaxel and non-targeting siRNA + paclitaxel treated Detroit 562, FaDu and SCC25 cells as well as Cx43 plasmid + paclitaxel and control plasmid + paclitaxel treated FaDu cells was determined by the direct counting of cells after 0.4% Trypan Blue solution (Sigma-Aldrich, St. Louis, MO, USA) staining in 1:1 ratio. Cell number was counted with hemocytometer. Absolute IC_50_ values were calculated by non-linear regression using Graph Pad Prism 5 software. Each experiment was repeated at least three times.

### 4.4. Quantitative Real-Time PCR (qPCR)

Total RNA was purified from the cells by using RNeasy Plus Mini Kit (Qiagen, Hilden, Germany) following the manufacturer’s instruction. cDNA was produced by reverse transcription of 1 μg DNA free RNA samples using SuperScript III First-Strand Synthesis System for RT-PCR Kit (Thermo Fisher Scientific, Waltham, MA, USA).

Quantitative real-time PCR assay was performed in 20 μL final volume containing 5 μL cDNA, 1× Prime Time Gene Expression Master Mix (Cat. No. 1055770, IDT, Coralville, IA, USA), 1× Prime Time qPCR assay (BCL2 (Cat. No. Hs.PT.56a.654557.g), GJA1 (Cat. No. Hs.PT.56a.38338544) and TUBA4A (Cat. No. Hs.PT.58.4392157.g); IDT)) using QuantStudio 12K Flex Software v1.2.2. Denaturation at 95 °C, 3 min was followed by 40 cycles (95 °C, 5 s and 60 °C, 30 s). Reactions were performed in triplicate using RNase-free water as negative control. CT-values were set in the exponential range of the amplification plots using the QuantStudio Detection Software. Relative expression levels were expressed as 2−ΔΔCT where ΔΔCT values correspond to the difference between the CT-values of the target and the TUBA4A internal control genes.

### 4.5. Western Blot Analysis

Cells were grown until 90% confluence in 6 well plates and incubated for 48 h in medium. After incubation, cells were washed with ice-cold PBS and lysed in lysis buffer (50 mM Tris (pH 7.4), 150 mM NaCl, 1% (V/V) NP-40, 2 mM EDTA, 2 mM EGTA, 1 mM dithiothreitol, phosphatase inhibitor cocktail (Merck, Kenilworth, NJ, USA) and protease inhibitor cocktail (Calbiochem)) for 30 min on ice. Lysates were centrifuged with 13,000× *g* at 4 °C for 15 min. Then, 10 μg protein samples were subjected to SDS-PAGE and electrotransferred to polyvinylidene-difluoride (PVDF) membranes. Membranes were incubated with the diluted primary antibodies at 4 °C overnight, and with horse radish peroxidase (HRP) conjugated secondary antibodies for 1 h at room temperature. Bcl-2 (clone 124, Cat. No. 15071, dilution 1:1000), Connexin 43 (Cat. No. 3512, dilution 1:1000), GFP (clone 4B10, Cat. No. 2955, dilution 1:1000) monoclonal antibodies were purchased from Cell Signaling Technology (Danvers, MA, USA) and α-tubulin (clone DM1A, Cat. No. T9026, dilution 1:40,000) monoclonal antibody was purchased from Merck Millipore (Burlington, MA, USA). Anti-mouse IgG (Cat. No. 7076, dilution 1:8000), Anti-rabbit IgG (Cat. No. 7054, dilution 1:2000) secondary antibodies were purchased from Cell Signaling Technology. Bands were visualized by Enhanced Chemiluminescence (ECL) detection system (Perkin Elmer, Waltham, MA, USA) and quantified by ImageJ v1.48 software. Every experiment was carried out at least three times. The original western blots found in the Supplementary Materials (Figures S4–S10).

### 4.6. Immunofluorescence Imaging of Cells

Cells were seeded into Ibidi μ-Slide 8 Well microscopic slide at the density of 2 × 10^4^ cells/well. After incubation, cells were washed with PBS and fixed in 4% paraformaldehyde solution (Bio-Optica) for 10 min. Then cells were washed with PBS and cell membrane was permeabilized with PBS containing 0.1% (V/V) Triton-X 100 for 15 min. After permeabilization, cells were washed three times with PBS for 5 min and blocking solution (PBS containing 10% (V/V) inactivated FBS) was added to each well and the plate was incubated for 1 h at room temperature. Blocking solution was discarded and diluted Bcl-2 (124) or Connexin 43 primary antibody in PBS containing 10% (V/V) FBS was added to the wells. Connexin 43 (Cat. No. 3512, dilution 1:100) monoclonal antibody was purchased from Cell Signaling Technology. Bcl-2 Monoclonal Antibody (clone 100/D5, Cat. No. MA5-11757, dilution 1:100) was purchased from Thermo Fisher Scientific. Plate was incubated overnight at 4 °C. Next, the primary antibody solution was discarded, and cells were washed three times with PBS for 5 min. Alexa Fluor 488 conjugated anti-rabbit IgG (Cat. No. A11001, Jackson Immuno Research, Cambridgeshire, United Kingdom) was diluted in PBS containing 10% (V/V) FBS (dilution 1:500), added to each well and incubated for 1 h at room temperature in the dark. Cells were washed three times with PBS for 5 min and were incubated with 10 µM Draq5™ in PBS for 10 min at room temperature in the dark. The wells were washed three times with PBS for 5 min and a few drops of mounting media (Cat. No. F4680-25ML, Merck Millipore) were added. Cells were analyzed using confocal laser microscope (Zeiss Confocal LSM 710, Carl Zeiss AG, Oberkochen, Germany) under 63× oil-immersion objective. Images were created and exported by Zen lite 2.5 software.

### 4.7. Flow Cytometry Analysis

Thus, 3 × 10^4^ of Detroit 562, FaDu and SCC25 cells were seeded into each well of a 24 well plate and let to attach for 48 h. Then culture medium was changed to complete medium containing paclitaxel at the indicated concentrations. After 48 h, supernatants were collected into polypropylene test tubes. Cell cultures were washed with 150 µL PBS/well that was also pipetted into the respective test tube. Then cells were trypsinized with 135 µL trypsin-EDTA (GIBCO, 15 min, 37 °C), suspended with 150 µL complete medium and 500 µL PBS and pipetted into the respective test tubes.

For apoptosis detection, trypsinized cell suspensions were centrifuged (250× *g*, 4 min, room temperature) and supernatants were removed. Pellets were washed once with 1 mL PBS, centrifuged (250× *g*, 4 min, room temperature) and incubated with 100 µL Binding Buffer supplemented with 2 µL Annexin V-FITC conjugate (20 min, room temperature, dark) as recommended by the manufacturer (ROCHE, Ref.: 11828681001). After the incubation, 1 mL PBS was pipetted into each tube and cells were centrifuged again as a washing step (250× *g*, 4 min, room temperature). Pellets were suspended in 300 µL PBS supplemented with 3 µL propidium iodide (10 ng/mL final concentration). Compensation was adjusted and the proportion of fluorescent cell populations was detected with a FACSCalibur flow cytometer using CellQuest Pro software (BD Biosciences, San Jose, CA, USA). Sample evaluation was performed with CellQuest Pro and Excel (Microsoft) software. Treatment groups were replicated at least three times.

### 4.8. RNA Interference

In total, 2 × 10^5^ of Detroit 562, FaDu and SCC25 cells were seeded into each well of a 6 well plate and let to attach for 48 h. Gap junction protein alpha 1 (*GJA1*) short interfering RNAs (ON-TARGETplus Human GJA1 (2697) siRNA–SMARTpool, Cat. No. L-011042-00-0005) were purchased from Dharmacon (Table 1). Ambion Non-targeting control #1 siRNA (Silencer® Select Negative Control #1 siRNA, Cat. No. 4390843) was ordered from Thermo Fisher Scientific. Transfection was carried out using Lipofectamine RNAiMAX (Cat. No. 13778075, Thermo Fisher Scientific) and OPTI-MEM media (Cat. No. 31985070, GIBCO) at the indicated concentrations following the manufacturers’ protocols. After 24 h, medium was changed to cell culture medium. Cells were used for other experiments (trypan blue exclusion test, western blot analysis and flow cytometry analysis) after additional 24 h of incubation.

### 4.9. Overexpression of Cx43

Cx43 was overexpressed in HNSCC cell lines using a pIRES2-Cx43 vector coding for wild-type Cx43 (wtCx43), kindly gifted by Professor David Laurence Becker (Lee Kong Chian School of Medicine, Nanyang Technological University, Singapore) [39]. A GFP-producing plasmid was used as a control. 10^5^ of Detroit 562, FaDu and SCC25 cells were seeded into each well of a 12 well plate and let to attach for 48 h. Transfection into the HNSCC cells was performed using Lipofectamine® LTX & PLUS™ Reagent (Cat. No. 15338100, Thermo Fisher Scientific), according to the manufacturer’s instructions. Cells were used for experiments (trypan blue exclusion test, western blot analysis and flow cytometry analysis) described in the proper sections.

### 4.10. Patients

Altogether 58 therapy naive patients were consecutively enrolled who were diagnosed with squamous cell carcinoma of the oropharynx, hypopharynx, and larynx at the Department of Oto-Rhino-Laryngology and Head and Neck Surgery, Semmelweis University between 2012 and 2014. All subjects gave their informed consent for inclusion before they participated in the study. The study was conducted in accordance with the Declaration of Helsinki, and the protocol was approved by the Semmelweis University’s Regional, Institutional Scientific and Research Ethics Committee (ethical license No: 105/2014). The most important characteristics of our cohort are shown in Table 2.

### 4.11. Tissue Microarray (TMA) and Immunohistochemistry

TMA blocks containing 2 mm diameter cores of formalin-fixed, paraffin-embedded (FFPE) tissue samples were created using the TMA Master tool (3DHISTECH Kft, Budapest, Hungary). Tissue sections (4 μm) were cut on adhesion slides and were stained with hematoxylin and eosin, Bcl-2 and Cx43. Antibodies used for immunohistochemistry are the same as those used for western blot.

BenchMark ULTRA IHC/ISH (Ventana Medical Systems, Oro Valley, AZ, USA) semi-automated device was used for immunohistochemical staining with the application of U ultraView DAB v1.02.0018 kit. The protocol of staining method was carried out as described previously [4].

Briefly, sections were incubated at 72 °C for 4 min. We used EZ Prep Solution (Ventana) three times to remove paraffin. Cell conditioning solution pH 9 (Ventana) was used for heat-induced epitope retrieval at 95 °C for 8 min followed by a heating at 97 °C for 76 min. Endogenous peroxidase activity was inhibited with one drop UV INHIBITOR (Ventana), which was applied at 36 °C for 4 min. Primary monoclonal antibody against Bcl-2 and Cx43 was applied at 36 °C for 120 min in a dilution of 1:100 respectively. After incubation with UV HRP UNIV MULT secondary antibody solution (Ventana) at 36 °C for 8 min, peroxidase activity was visualized with diaminobenzidine (DAB) chromogen (Ventana). Nuclear counterstaining was done with hematoxylin II (Ventana). All washing steps were performed with diluted Reaction Buffer Concentrate (Ventana).

Bcl-2 categories were: 1: <10% positive tumor cells; 2: 11 to 30% positive tumor cells, 3: 31 to 60% positive tumor cells; and 4: >60% positive tumor cells [40,41]. Cx43 staining was evaluated as follows: 1: <5% positive tumor cells; 2: 6 to 20% positive tumor cells, 3: 21 to 60% positive tumor cells; and 4: >60% positive tumor cells [14]. For statistical analysis, scores were dichotomized along different threshold values. The most reproducible threshold for all assessors was set up when scores 1 and 2 were considered low protein expression, whereas scores 3 and 4 were taken high protein expression.

### 4.12. Statistical Analysis

Statistica 13 (TIBCO Software Inc., Palo Alto, CA, USA) software was used to carry out the statistical analysis, measured values are indicated as mean ± standard deviation (SD). Student’s *t*-test was used to compare groups. Two-sided test was selected. *p* < 0.05 value was considered as statistically significant and all measurements were performed at least 3 times. When we examined the protein and mRNA expression of HNSCC cell lines, Cx43 and Bcl-2 protein and mRNA expression of the cells were compared to each other (Tables S2–S7). To determinate the IC_50_ value of paclitaxel on cell viability of the studied cell line, we compared the IC_50_ points to each other (Tables S8 and S9). The paclitaxel-induced apoptosis on HNSCC cell lines was examined in each case and in all concentration by the comparison of the treated cell fractions to the control fractions (Tables S10–S13). When we investigated the effects of *GJA1* siRNA or Cx43 plasmid on the protein expression of HNSCC cell lines, the expression pattern of all proteins in *GJA1* siRNA treated samples were compared to protein expression of the non-targeting siRNA treated cell samples, this method was applied in each cell line (Tables S14 and S15). The expression pattern of all proteins in Cx43 plasmid treated samples were compared to protein expression of the control plasmid treated cell samples, this method was applied in each cell line (Tables S16 and S17). Analyzing cell viability in the *GJA1* siRNA or Cx43 plasmid and paclitaxel treated cell samples, the IC_50_ values of the cell lines were compared to each other (Tables S18 and S25). When examining the effects of *GJA1* siRNA on the paclitaxel-induced apoptosis of HNSCC cell lines in non-targeting siRNA and *GJA1* siRNA treated samples, the cell fractions -in each concentration- were compared to the negative control fractions. The cell fractions of *GJA1* siRNA treated samples were also compared to the cell fractions of non-targeting siRNA treated samples (Tables S19–S24).

For patient data, statistical analysis was performed using IBM SPSS Statistics for Mac version 20.0.0 (SPSS Inc., Chicago, IL, USA). The Pearson χ^2^ tests and the Fisher’s exact tests were used to test correlations between discrete variables. In case of survival analysis, Kaplan-Meier estimation with log-rank test as well as univariate and multivariate regression were applied. All tests were two-sided and *p*-values <0.05 were considered statistically significant. Tumor localization, tumor size, stage, lymph node metastasis, response to neoadjuvant chemotherapy and the biomarkers listed above were used in the analysis (Table S26).

## 5. Conclusions

Based on these findings, we hypothesize that Cx43 expression has a control on Bcl-2 protein levels. Elevated Cx43 and the concomitant low Bcl-2 levels result in an increased paclitaxel sensitivity. Since taxanes are key components of induction chemotherapy for HNSCCs, testing for these biomarkers in head and neck cancer patients might allow improved treatment planning and outcome prediction. Systemic retrospective analyses evaluating the possible correlation between therapeutic response and the above molecular alterations can further validate our results.

## Figures and Tables

**Table 1:** Gap junction protein alpha 1 (*GJA1*) short interfering RNA target sequences.

siRNA No.	Target Sequence
J-011042-05	CAGUCUGCCUUUCGUUGUA
J-011042-06	UGACAAGGUUCAAGCCUAC
J-011042-07	GUACAUCUAUGGAUUCAGC
J-011042-08	GAACCUACAUCAUCAGUAU

**Table 2:** Patient characteristics at time of diagnosis.

Variable	No. of Patients
Total no. of patients	58
Sex	
Male	52
Female	6
Age (year)	
Mean	58.45 (41–77)
Localization	
Oropharynx	20
Larynx	20
Hypopharynx	17
Oral cavity	1
TNM ^1^ stage	
I	6
II	9
III	13
IV A	23
IV B	4
IV C	3

^1^ TNM: tumor, node and metastasis, UICC TNM 7th edition.

## Supplementary Materials

The following are available online at https://www.mdpi.com/2072-6694/11/12/1848/s1, Figure S1: Changes in the paclitaxel-induced apoptosis of Detroit 562 after knocking down Cx43, Figure S2: Changes in the paclitaxel-induced apoptosis of FaDu after knocking down Cx43, Figure S3: Changes in the effect of paclitaxel on cell viability after knocking down Cx43, Figure S4: Expression of Cx43 and Bcl-2 in HNSCC cell lines and human skin epidermoid carcinoma cell line, A431, Figure S5: Changes in the levels of Cx43 and Bcl-2 after *GJA1* siRNA knockdown in HNSCC cell lines, Figure S6: Changes in the levels of Cx43 and Bcl-2 after Cx43 plasmid transfection in HNSCC cell lines, Figure S7: Changes in the paclitaxel-induced apoptosis of SCC25 after knocking down Cx43, Figure S8: Changes in the paclitaxel-induced apoptosis of Detroit 562 after knocking down Cx43, Figure S9: Changes in the paclitaxel-induced apoptosis of FaDu after knocking down Cx43, Figure S10: Changes in the effect of paclitaxel on cell viability of FaDu after transfection Cx43, Table S1: Changes in the effect of paclitaxel on cell viability after knocking down Cx43, Table S2: Expression of Cx43 and Bcl-2 in HNSCC cell lines, Table S3: Expression of Cx43 and Bcl-2 in HNSCC cell lines, Table S4: Expression of Cx43 and Bcl-2 in HNSCC cell lines, Table S5: Quantitative PCR analysis of Cx43 and Bcl-2 mRNA expression in HNSCC cell lines, Table S6: Quantitative PCR analysis of Cx43 and Bcl-2 mRNA expression in HNSCC cell lines, Table S7: Quantitative PCR analysis of Cx43 and Bcl-2 mRNA expression in HNSCC cell lines, Table S8: Effect of paclitaxel on cell viability, Table S9: Effect of paclitaxel on cell viability, Table S10. Paclitaxel-induced apoptosis of Detroit 562, FaDu and SCC25 cells, Table S11: Paclitaxel-induced apoptosis of Detroit 562 cells, Table S12: Paclitaxel-induced apoptosis of FaDu cells, Table S13. Paclitaxel-induced apoptosis of SCC25 cells, Table S14: Changes in the levels of Cx43 and Bcl-2 after *GJA1* siRNA knockdown in HNSCC cell lines, Table S15: Changes in the levels of Cx43 and Bcl-2 after *GJA1* siRNA knockdown in HNSCC cell lines, Table S16: Changes in the levels of Cx43 and Bcl-2 after Cx43 plasmid transfection in HNSCC cell lines, Table S17: Changes in the levels of Cx43 and Bcl-2 after Cx43 plasmid transfection in HNSCC cell lines, Table S18: Changes in the effect of paclitaxel on cell viability after knocking down Cx43, Table S19: Changes in the paclitaxel-induced apoptosis of SCC25 after knocking down Cx43, Table S20: Changes in the paclitaxel-induced apoptosis of SCC25 after knocking down Cx43, Table S21: Changes in the paclitaxel-induced apoptosis of Detroit 562 after knocking down Cx43, Table S22: Changes in the paclitaxel-induced apoptosis of Detroit 562 after knocking down Cx43, Table S23: Changes in the paclitaxel-induced apoptosis of FaDu after knocking down Cx43, Table S24: Changes in the paclitaxel-induced apoptosis of FaDu after knocking down Cx43, Table S25. Changes in the effect of paclitaxel on cell viability after transfection Cx43, Table S26: Data tables summarizing the results of Cx43 and Bcl-2 immunohistochemical analysis.
