# Supplementary material for: The Potential Impact of Connexin 43 Expression on Bcl-2 Protein Level and Taxane Sensitivity in Head and Neck Cancers–In Vitro Studies
Source: Cancers (Basel). 2019 Nov 22;11(12):1848. doi: 10.3390/cancers11121848 (PMC6966683; doi:10.3390/cancers11121848)
Supplement: Supplementary file 1 [file cancers-11-01848-s001.pdf]

Supplementary Materials: The Potential Impact of Connexin 43 Expression on Bcl-2 Protein Level and Taxane Sensitivity in Head and Neck Cancers–in Vitro Studies

Bianka Gurbi, Diána Brauswetter, Attila Varga, Pál Gyulavári, Kinga Pénzes, József Murányi, Veronika Zámbó, Ede Birtalan, Tibor Krenács, David Laurence Becker, Miklós Csala, István Vályi-Nagy, István Peták and Kornél Dános

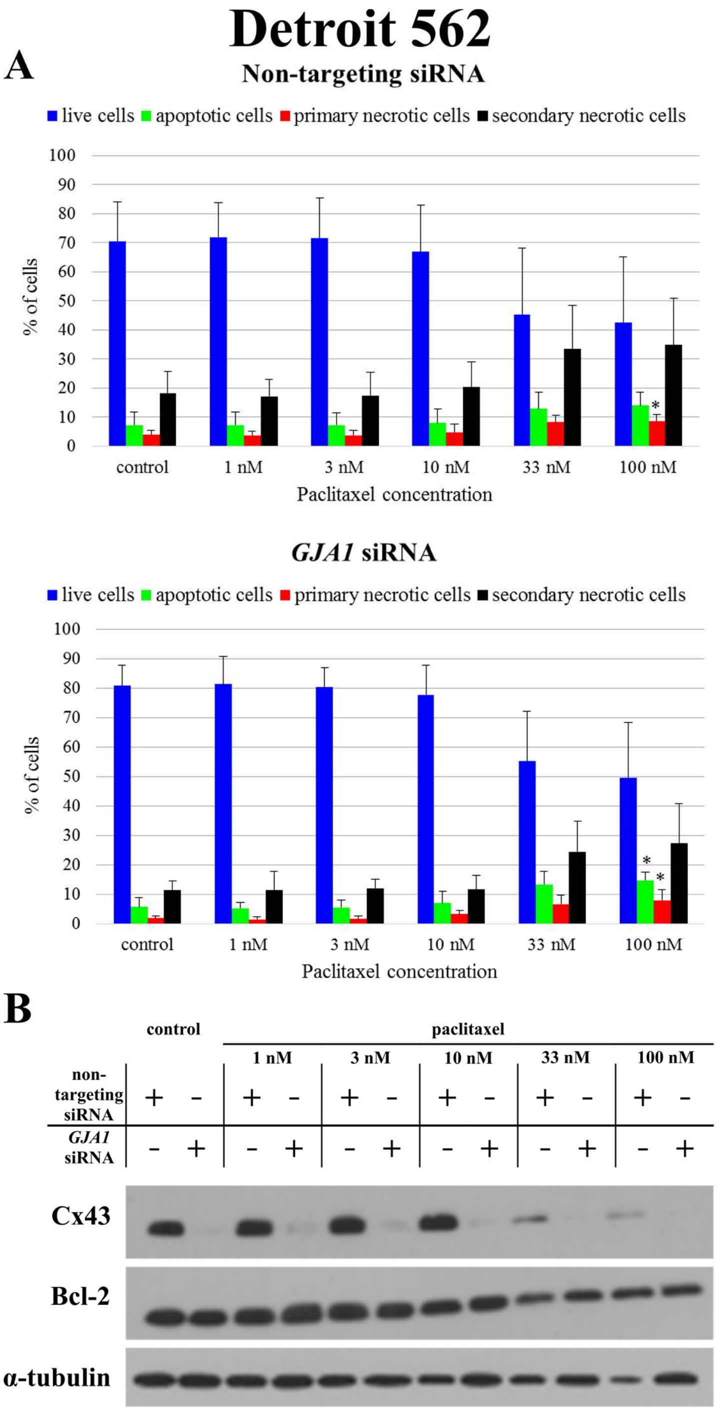

Figure S1. Changes in the paclitaxel-induced apoptosis of Detroit 562 after knocking down Cx43. (**A**) Annexin V-FLUOS/PI (Ann/PI)-stained HNSCC cells were analyzed by FACS after 48 h of treatment

with paclitaxel at different concentrations. Live cells are presented by the Ann-/PI- fraction, apoptotic cells by the Ann+/PI- fraction, secondary necrotic cells by the Ann+/PI+ fraction and primary necrotic cells are detected in the Ann-/PI+ fraction. Data are presented as mean ± SD. Statistical analysis was performed by Student's t-test, the cell fractions in all concentration were compared to negative control fractions in non-targeting siRNA or *GJA1* siRNA treated samples. The cell fractions in *GJA1* siRNA treated samples were also compared to cell fractions in non-targeting siRNA treated samples. *p < 0.05 (**B**) Cells were subjected to western blot analysis with antibodies against Cx43, Bcl-2 and the loading control, α-tubulin.

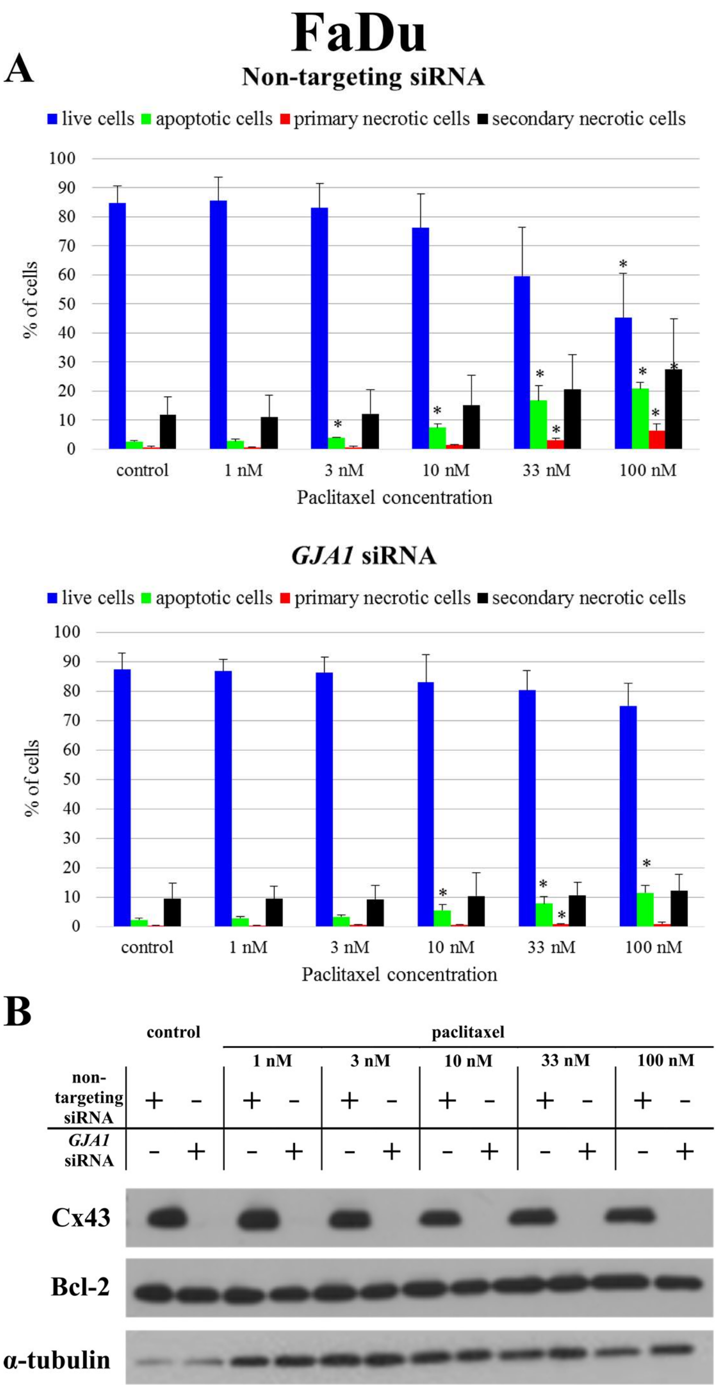

Figure S2. Changes in the paclitaxel-induced apoptosis of FaDu after knocking down Cx43. (**A**) Annexin V-FLUOS/PI (Ann/PI)-stained HNSCC cells were analyzed by FACS after 48 h of treatment with paclitaxel at different concentrations. Live cells are presented by the Ann-/PI- fraction, apoptotic cells by the Ann+/PI- fraction, secondary necrotic cells by the Ann+/PI+ fraction and primary necrotic

cells are detected in the Ann-/PI+ fraction. Data are presented as mean ± SD. Statistical analysis was performed by Student's t-test, the cell fractions in all concentration were compared to negative control fractions in non-targeting siRNA or *GJA1* siRNA treated samples. The cell fractions in *GJA1* siRNA treated samples were also compared to cell fractions in non-targeting siRNA treated samples. $*p < 0.05$ (**B**) Cells were subjected to western blot analysis with antibodies against Cx43, Bcl-2 and the loading control, α-tubulin.

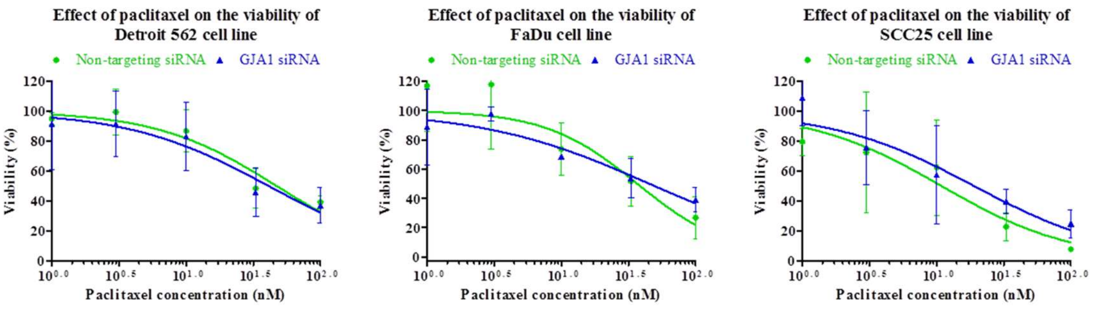

Figure S3. Changes in the effect of paclitaxel on cell viability after knocking down Cx43. HNSCC cell lines were analyzed by trypan blue exclusion test after 48 h of treatment with paclitaxel at different concentrations. IC_{50} curves of paclitaxel on Detroit 562, FaDu and SCC25 cell lines. The results represent the mean of three independent experiments with SD.

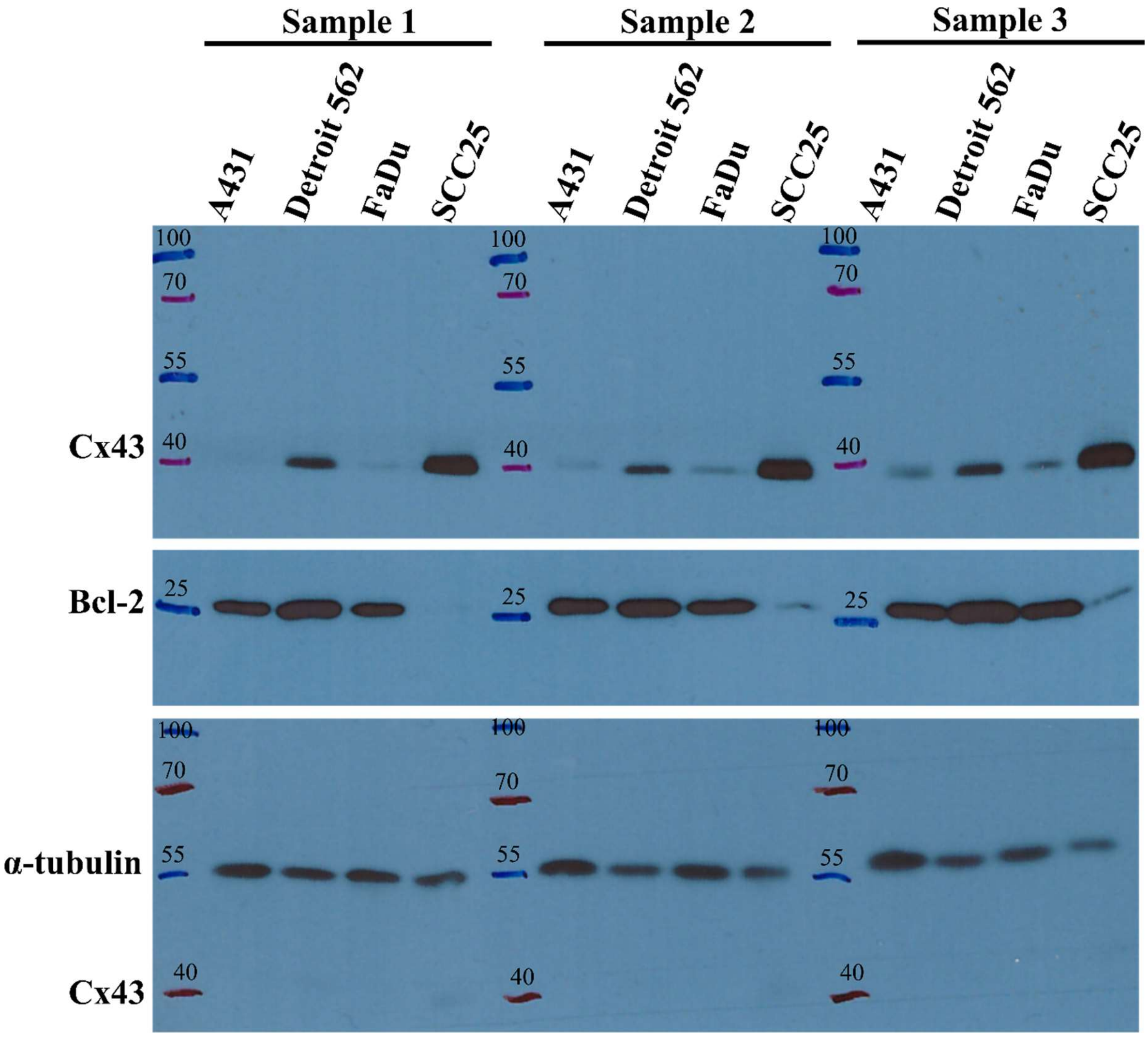

Figure S4. Expression of Cx43 and Bcl-2 in HNSCC cell lines and human skin epidermoid carcinoma cell line, A431. We were not used A431 in this article. Cells were subjected to western blot analysis with antibodies against Cx43, Bcl-2 and the loading control, α-tubulin. (Figure 1A).

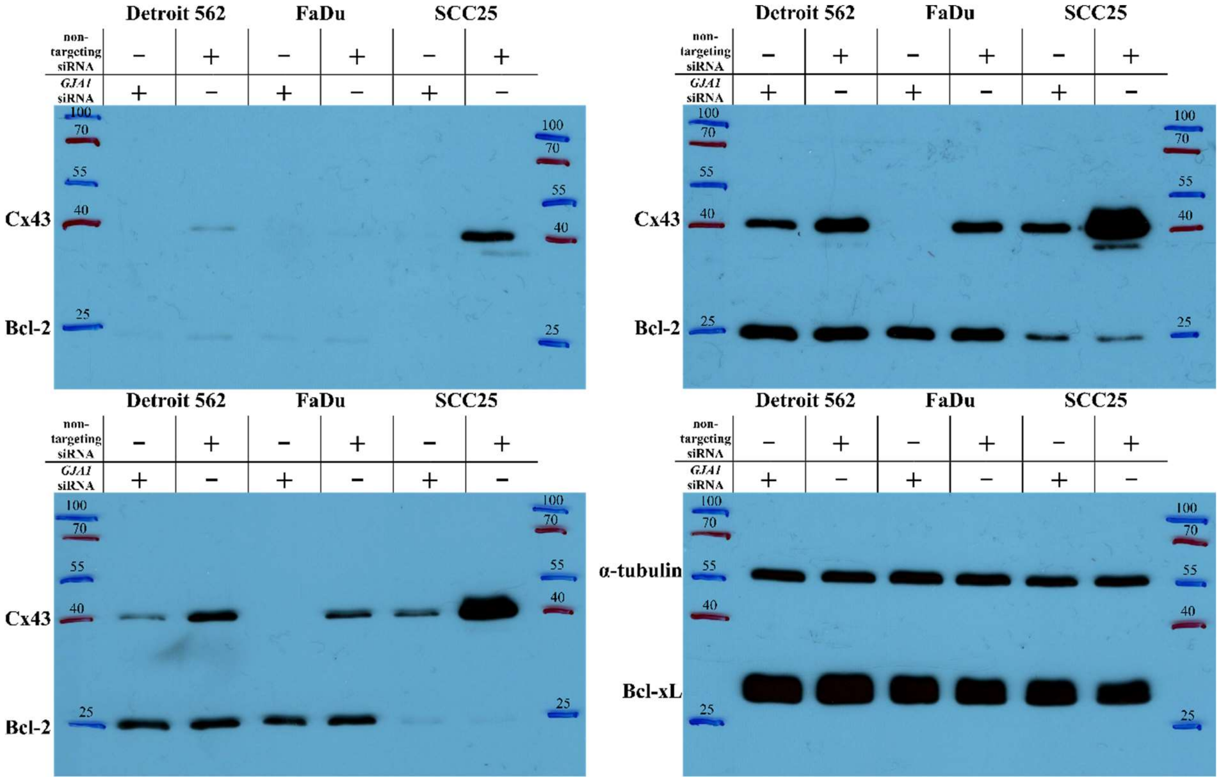

Figure S5. Changes in the levels of Cx43 and Bcl-2 after *GJA1* siRNA knockdown in HNSCC cell lines. Cells were subjected to western blot analysis with antibodies against Cx43, Bcl-2, Bcl-xL and the loading control, α-tubulin. We have not used Bcl-xL in this article. (Figure 4A).

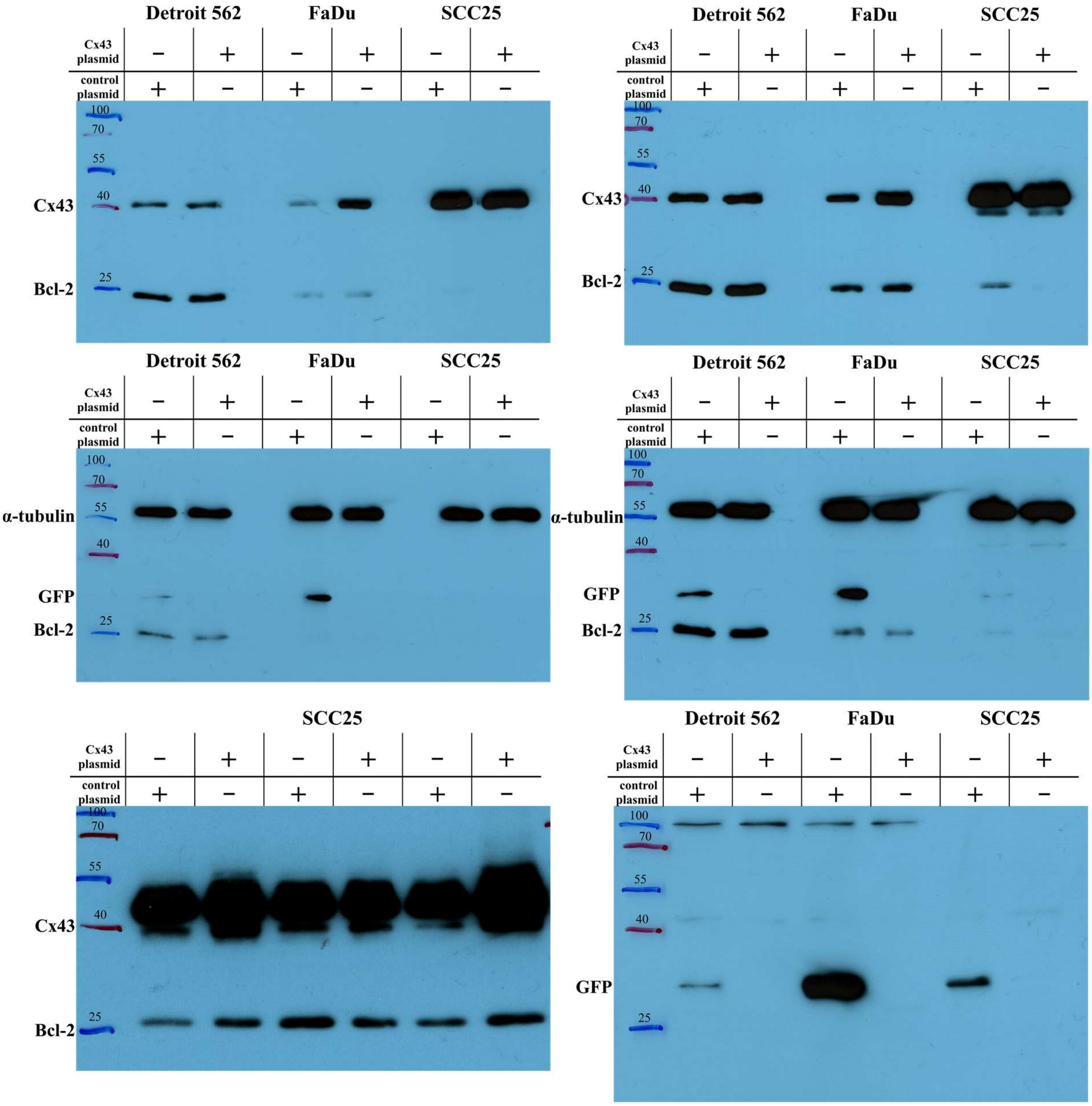

Figure S6. Changes in the levels of Cx43 and Bcl-2 after Cx43 plasmid transfection in HNSCC cell lines. Cells were subjected to western blot analysis with antibodies against Cx43, Bcl-2, GFP and the loading control, α-tubulin. (Figure 4B).

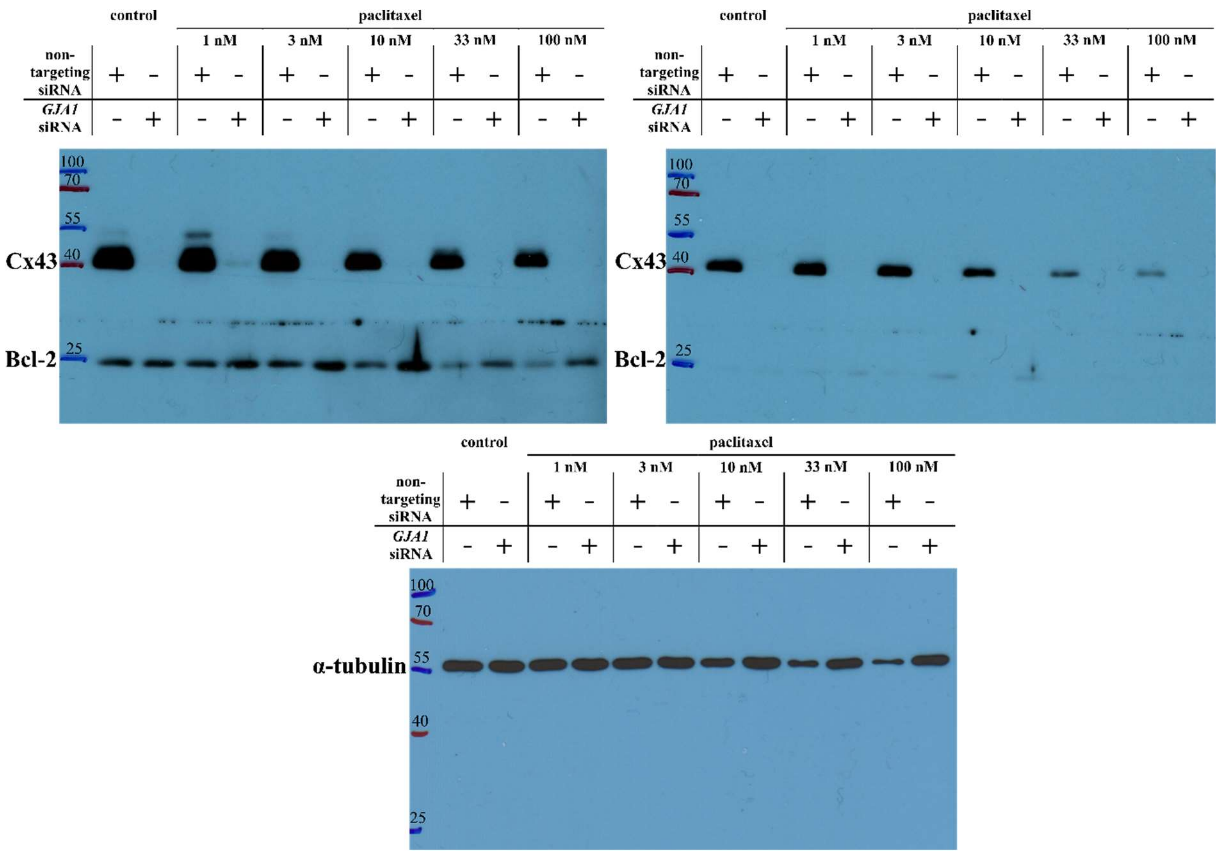

Figure S7. Changes in the paclitaxel-induced apoptosis of SCC25 after knocking down Cx43. Cells were subjected to a western blot analysis with antibodies against Cx43, Bcl-2 and the loading control, α-tubulin. (Figure 5B).

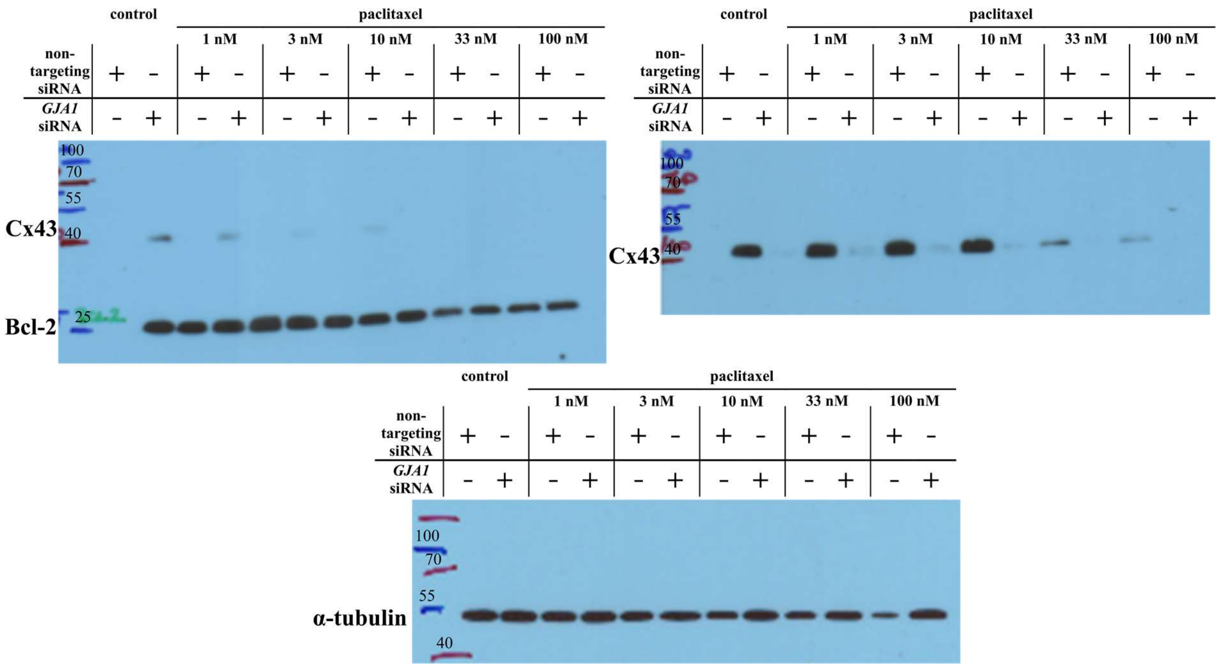

Figure S8. Changes in the paclitaxel-induced apoptosis of Detroit 562 after knocking down Cx43. Cells were subjected to a western blot analysis with antibodies against Cx43, Bcl-2 and the loading control, α-tubulin. (Figure S1).

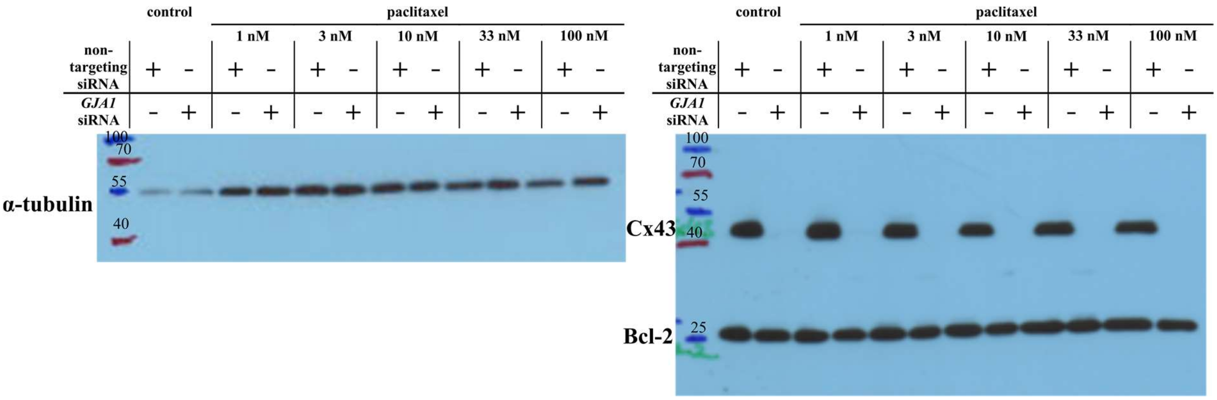

Figure S9. Changes in the paclitaxel-induced apoptosis of FaDu after knocking down Cx43. Cells were subjected to a western blot analysis with antibodies against Cx43, Bcl-2 and the loading control, α-tubulin. (Figure S2).

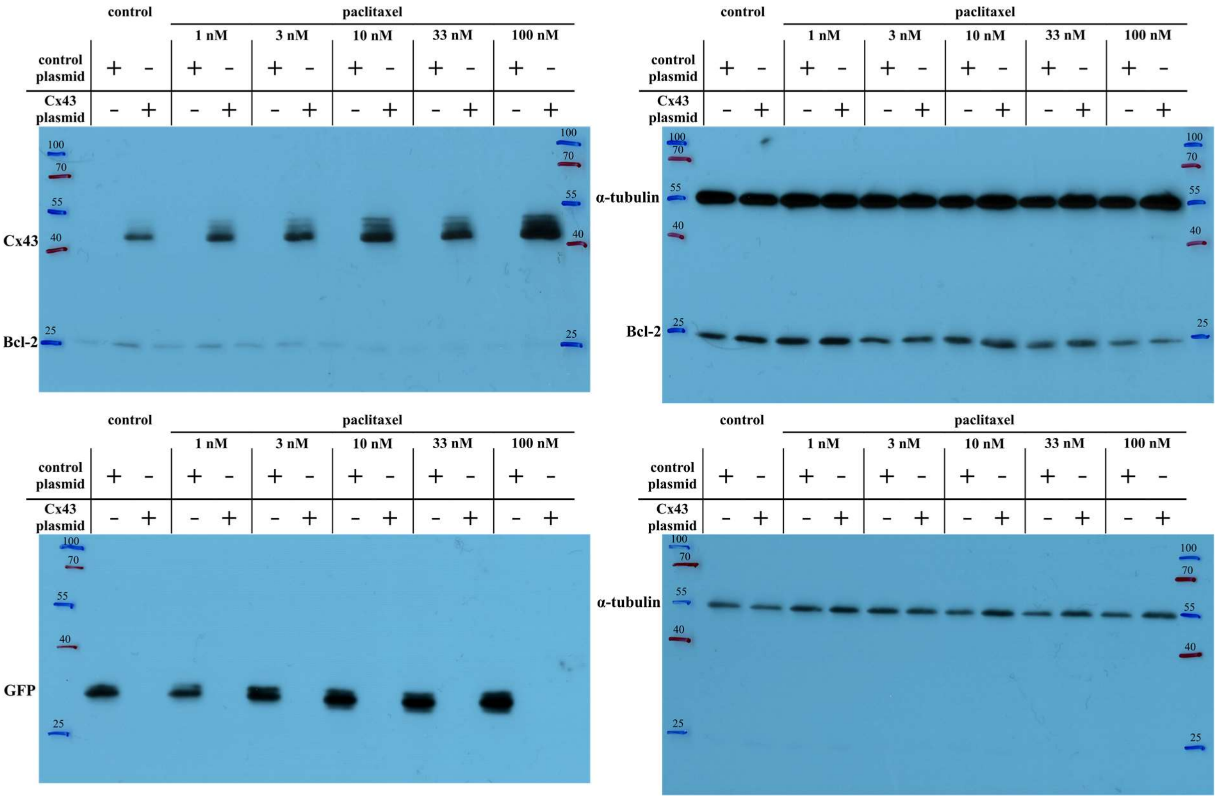

Figure S10. Changes in the effect of paclitaxel on cell viability of FaDu after transfection Cx43. FaDu cells were subjected to a western blot analysis with antibodies against Cx43, Bcl-2, GFP and the loading control, α-tubulin. (Figure 6C).

Table S1. Changes in the effect of paclitaxel on cell viability after knocking down Cx43. HNSCC cell lines were analyzed by trypan blue exclusion test after 48 h of treatment with paclitaxel at different concentrations. IC_{50} concentrations of paclitaxel measured on Detroit 562, FaDu and SCC25 cell lines. IC_{50} values are the mean of three different measurements.

	**Detroit 562**		**FaDu**		**SCC25**	
Non-targeting siRNA	+	-	+	-	+	-
GJA1 siRNA	-	+	-	+	-	+

paclitaxel	47.04 nM	41.01 nM	36.73 nM	45.11 nM	10.84 nM	18.93 nM

Table S2. Expression of Cx43 and Bcl-2 in HNSCC cell lines. Densitometry analysis was performed using three independent experiments. The expressions of all proteins were normalized to the expression of α-tubulin. (Figure 1B).

Cell line	Densitometry intensity of α-tubulin				
	Sample 1	Sample 2	Sample 3	Mean	SD
Detroit 562	9493.397	22227	30829.075	20849.66	10734.27
FaDu	14972.418	28803	33806.246	25860.68	9755.657
SCC25	8620.933	15800	22190.953	15537.18	6788.816
Cell line	Densitometry intensity of Cx43				
	Sample 1	Sample 2	Sample 3	Mean	SD
Detroit 562	9949.619	11556	18387.453	13297.83	4480.364
FaDu	4781.426	5814	9385.533	6660.246	2415.953
SCC25	21749.953	28788	33362.541	27966.81	5849.677
Cell line	Densitometry intensity ratio of Cx43				
	Sample 1	Sample 2	Sample 3	Mean	SD
Detroit 562	1.05	0.52	0.60	0.72	0.29
FaDu	0.32	0.20	0.28	0.27	0.06
SCC25	2.52	1.82	1.50	1.95	0.52
Cell line	Densitometry intensity of Bcl-2				
	Sample 1	Sample 2	Sample 3	Mean	SD
Detroit 562	17638.912	17397.569	22557.276	19197.92	2911.79
FaDu	11103.083	14597.983	16935.296	14212.12	2935.19
SCC25	690.456	1744.406	2769.497	1734.79	1039.55
Cell line	Densitometry intensity ratio of Bcl-2				
	Sample 1	Sample 2	Sample 3	Mean	SD
Detroit 562	1.86	0.78	0.73	1.12	0.64
FaDu	0.74	0.51	0.50	0.58	0.14
SCC25	0.08	0.11	0.12	0.11	0.02

Table S3. Expression of Cx43 and Bcl-2 in HNSCC cell lines. Densitometry analysis was performed using three independent experiments. The expressions of all proteins were normalized to the expression of α-tubulin. Statistical analysis was performed by Student's t-test, the Cx43 expression of the cell lines were compared to each other. Red color indicate if $p < 0.05$. (Figure 1B).

Group 1 vs. Group 2	T-test for Independent Samples (Cx43. Bcl2 exp) Note: Variables were treated as independent samples										
	Mean Group1	Mean Group2	t-value	df	p	N 1	N 2	Std.Dev Group1	Std.Dev Group2	F-ratio Variances	p Variance
Detroit 562 Cx43/tubulin vs. FaDu Cx43/tubulin	0.721476	0.266273	2.70426	4	0.053856	3	3	0.28540	0.059570	22.95436	0.083492
Detroit 562 Cx43/tubulin vs. SCC25 Cx43/tubulin	0.721476	1.949472	-3.57751	4	0.023219	3	3	0.28540	0.521552	3.33950	0.460883
FaDu Cx43/tubulin vs. SCC25 Cx43/tubulin	0.266273	1.949472	-5.55372	4	0.005144	3	3	0.05957	0.521552	76.65604	0.025755

Table S4. Expression of Cx43 and Bcl-2 in HNSCC cell lines. Densitometry analysis was performed using three independent experiments. The expressions of all proteins were normalized to the expression of α-tubulin. Statistical analysis was performed by Student's t-test, the Bcl-2 expression of the cell lines were compared to each other. Red color indicate if $p < 0.05$. (Figure 1B).

Group 1 vs. Group 2	T-test for Independent Samples (Cx43. Bcl2 exp) Note: Variables were treated as independent samples										
	Mean Group 1	Mean Group 2	t-value	df	p	N 1	N 2	Std.Dev. Group 1	Std.Dev. Group 2	F-ratio Variance	p Variance
Detroit 562 Bcl2/tubulin vs. FaDu Bcl2/tubulin	1.124149	0.583112	1.44014	4	0.223251	3	3	0.636063	0.137259	21.4742	0.088991
Detroit 562 Bcl2/tubulin vs. SCC25 Bcl2/tubulin	1.124149	0.105101	2.77317	4	0.050168	3	3	0.636063	0.022824	776.6460	0.002572
FaDu Bcl2/tubulin vs. SCC25 Bcl2/tubulin	0.583112	0.105101	5.95024	4	0.004003	3	3	0.137259	0.022824	36.1665	0.053812

Table S5. Quantitative PCR analysis of Cx43 and Bcl-2 mRNA expression in HNSCC cell lines. Quantitative PCR analysis was performed using three independent experiments. The expressions of all mRNAs were normalized to the expression of α-tubulin. (Figure 1B).

Cell line	Ct of α-tubulin				
	Sample 1	Sample 2	Sample 3	Mean	SD
Detroit 562	25.5667	24.7533	26.1200	25.4800	0.6874
FaDu	27.9167	28.6100	28.8467	28.4578	0.4833
SCC25	25.2867	25.2800	25.3933	25.3200	0.0636
Cell line	Ct of Cx43				
	Sample 1	Sample 2	Sample 3	Mean	SD
Detroit 562	28.5867	28.3467	28.9600	28.6311	0.3091
FaDu	31.3833	31.2000	31.3267	31.3033	0.0939
SCC25	26.4733	25.8433	25.5467	25.9544	0.4732
Cell line	$2^{-\delta\delta Ct}$ of Cx43				
	Sample 1	Sample 2	Sample 3	Mean	SD
Detroit 562	1.00	0.67	1.13	0.93	0.24
FaDu	0.73	1.35	1.45	1.18	0.39
SCC25	3.56	5.49	7.29	5.45	1.87
Cell line	Ct of Bcl-2				
	Sample 1	Sample 2	Sample 3	Mean	SD
Detroit 562	28.8967	29.0400	29.4967	29.1444	0.3133
FaDu	32.2200	32.7267	32.6367	32.5278	0.1848
SCC25	34.1600	33.7967	34.0367	33.9978	0.2703
Cell line	$2^{-\delta\delta Ct}$ of Bcl-2				
	Sample 1	Sample 2	Sample 3	Mean	SD
Detroit 562	1.00	0.52	0.97	0.83	0.27
FaDu	0.51	0.58	0.73	0.61	0.11
SCC25	0.02	0.03	0.03	0.02	0.00

Table S6. Quantitative PCR analysis of Cx43 and Bcl-2 mRNA expression in HNSCC cell lines. Quantitative PCR analysis was performed using three independent experiments. The expressions of all mRNAs were normalized to the expression of α-tubulin. Statistical analysis was performed by Student's t-test, the Cx43 mRNA expression of the cell lines were compared to each other. Red color indicate if $p < 0.05$. (Figure 1B).

	T-test for Independent Samples (Spreadsheet1) Note: Variables were treated as independent samples										
Group 1 vs. Group 2	Mean Group1	Mean Group2	t-value	df	p	N 1	N 2	Std.Dev Group1	Std.Dev Group2	F-ratio Variances	p Variance
Detroit 562 Cx43/tubulin vs. FaDu Cx43/tubulin	0.83	0.606667	1.327318	4	0.255094	3	3	0.26889	0.112398	5.722955	0.297488
Detroit 562 Cx43/tubulin vs. SCC25 Cx43/tubulin	0.83	0.026667	5.173532	4	0.006636	3	3	0.26889	0.005774	2169	0.000922
FaDu Cx43/tubulin vs. SCC25 Cx43/tubulin	0.6066667	0.026667	8.926012	4	0.000871	3	3	0.1124	0.005774	379	0.005263

Table S7. Quantitative PCR analysis of Cx43 and Bcl-2 mRNA expression in HNSCC cell lines. Quantitative PCR analysis was performed using three independent experiments. The expressions of all mRNAs were normalized to the expression of α-tubulin. Statistical analysis was performed by Student's t-test, the Bcl-2 mRNA expression of the cell lines were compared to each other. Red color indicate if $p < 0.05$. (Figure 1B).

	T-test for Independent Samples (Spreadsheet1) Note: Variables were treated as independent samples										
Group 1 vs. Group 2	Mean Group 1	Mean Group 2	t-value	df	p	N 1	N 2	Std.Dev. Group 1	Std.Dev. Group 2	F-ratio Variance	p Variance
Detroit 562 Bcl2/tubulin vs. FaDu Bcl2/tubulin	0.93333333	1.176667	-0.923311	4	0.4081	3	3	0.237136	0.390043	2.705394	0.539754
Detroit 562 Bcl2/tubulin vs. SCC25 Bcl2/tubulin	0.93333333	5.446667	-4.157288	4	0.014177	3	3	0.237136	1.865378	61.87848	0.031807
FaDu Bcl2/tubulin vs. SCC25 Bcl2/tubulin	1.17666667	5.446667	-3.880874	4	0.017828	3	3	0.390043	1.865378	22.87226	0.083779

Table S8. Effect of paclitaxel on cell viability. HNSCC cell lines were analyzed in parallel by MTT after 72 h of treatment with paclitaxel at different concentrations. (Figure 2A).

Concentration (nM)	Effect of paclitaxel on Detroit 562 cell viability (%)				
	Sample 1	Sample 2	Sample 3	Mean	SD
100.00	10.97397	17.44524	16.26879	14.89599946	3.447132
33.33	11.82995	19.23695	16.93683	16.00124435	3.791092
11.11	13.67327	24.68054	20.85667	19.73682617	5.588427
3.70	32.6012	36.0533	30.11101	32.9218389	2.984093
1.23	76.37317	81.49781	68.76903	75.54667124	6.40451
0.41	98.64927	99.36199	116.6716	104.8942815	10.20566
0.14	93.33649	106.569	128.8044	109.5699646	17.92338
0.05	103.9467	104.5035	136.3199	114.9233522	18.53202
0.02	107.449	110.042	135.9564	117.815795	15.76361
0.01	113.148	105.0265	134.915	117.6964845	15.45471
**Concentration (nM)**	**Effect of paclitaxel on FaDu cell viability (%)**				
	Sample 1	Sample 2	Sample 3	Mean	SD
100.00	26.97407	18.69288	17.36308	21.01000832	5.20765
33.33	21.90802	18.58977	17.91108	19.46962489	2.138807
11.11	26.42613	26.10844	22.50121	25.01192614	2.180138
3.70	34.58415	38.98146	34.92637	36.16399514	2.445991
1.23	44.62818	46.50157	39.06974	43.39983209	3.865186
0.41	59.59638	58.12052	64.75204	60.82297985	3.481764
0.14	75.50636	80.74625	79.59072	78.61444257	2.752988
0.05	107.3973	100.6244	98.78542	102.2690208	4.535369
0.02	106.8664	123.8793	99.95448	110.2334131	12.31267
0.01	112.6663	131.623	102.8749	115.7214018	14.61549
**Concentration (nM)**	**Effect of paclitaxel on SCC25 cell viability (%)**				
	Sample 1	Sample 2	Sample 3	Mean	SD
100.00	7.531455	9.819512	7.07535	8.142105351	1.470468
33.33	7.336523	8.296825	6.449693	7.361013998	0.92381
11.11	8.924331	8.047916	7.475839	8.149362276	0.729555
3.70	11.71008	10.30137	11.23868	11.08337893	0.717081
1.23	25.79833	20.11295	18.74177	21.55101838	3.741631
0.41	39.48254	28.35175	35.93697	34.59041981	5.68626
0.14	68.25093	55.27339	57.31832	60.28087928	6.977587

0.05	85.3624	80.34327	74.89183	80.19916739	5.236769
0.02	94.06699	95.1515	85.4544	91.55762868	5.313292
0.01	104.0121	106.7755	93.04794	101.2784911	7.260549

Table S9. Effect of paclitaxel on cell viability. HNSCC cell lines were analyzed in parallel by MTT after 72 h of treatment with paclitaxel at different concentrations. Statistical analysis was performed by Student's t-test, the IC_{50} concentrations of the cell lines were compared to each other. Red color indicate if $p < 0.05$. (Figure 2B).

Group 1 vs. Group 2	T-test for Independent Samples (Paclitaxel_IC50) Note: Variables were treated as independent samples										
	Mean Group 1	Mean Group 2	t-value	df	p	N 1	N 2	Std.Dev. Group 1	Std.Dev. Group 2	F-ratio Variances	p Variances
Detroit 562 IC50 vs. FaDu IC50	2.920667	1.513000	3.7537	4	0.019883	3	3	0.616249	0.205263	9.01342	0.199732
Detroit 562 IC50 vs. SCC25 IC50	2.920667	0.238200	7.4872	4	0.001702	3	3	0.616249	0.072920	71.42055	0.027616
FaDu IC50 vs. SCC25 IC50	1.513000	0.238200	10.1364	4	0.000533	3	3	0.205263	0.072920	7.92380	0.224120

Table S10. Paclitaxel-induced apoptosis of Detroit 562, FaDu and SCC25 cells. Annexin V-FLUOS/PI (Ann/PI)-stained HNSCC cells were analyzed by FACS after 48 h of treatment with paclitaxel at different concentrations. Live cells are presented by the Ann-/PI- fraction (LL), apoptotic cells by the Ann+/PI- fraction (LR), secondary necrotic cells by the Ann+/PI+ fraction (UR) and primary necrotic cells are detected in the Ann-/PI+ fraction (UL). (Figure 3).

Concentration (nM)		Paclitaxel-induced apoptosis of Detroit 562 cell (%)				
		Sample 1	Sample 2	Sample 3	Mean	SD
Control	UL	3.14	0.5	2.86	2.2	1.450149
	UR	3.6	3.2	7.72	4.8	2.502159
	LL	81.22	93.39	87.35	87.3	6.085055
	LR	12.03	2.91	2.07	5.7	5.523912
1	UL	2.36	0.31	2.58	1.8	1.251919
	UR	4.88	2.29	8.93	5.4	3.346645
	LL	80.55	94.86	84.19	86.5	7.437233
	LR	12.21	2.54	4.3	6.4	5.150641
3	UL	4.78	0.26	6.94	4.0	3.408773
	UR	5.36	2.41	16.3	8.0	7.317994
	LL	74.43	94.43	70.94	79.9	12.67517
	LR	15.43	2.9	5.82	8.1	6.555906
10	UL	12.23	0.81	5.9	6.3	5.721209
	UR	14.22	11.29	21.12	15.5	5.046844
	LL	51.36	76.25	62.87	63.5	12.4567
	LR	22.19	11.65	10.11	14.7	6.575074

33	UL	9.16	0.89	7.03	5.7	4.293976
	UR	12.73	12.01	31.64	18.8	11.13136
	LL	52.86	74.33	51.25	59.5	12.88565
	LR	25.25	12.77	10.07	16.0	8.098074
100	UL	8.93	1.54	5.15	5.2	3.695326
	UR	9.35	13.56	31.65	18.2	11.84808
	LL	51.21	71.28	52.2	58.2	11.31247
	LR	30.5	13.62	11	18.4	10.58339

Concentration (nM)		Paclitaxel-induced apoptosis of FaDu cell (%)				
		Sample 1	Sample 2	Sample 3	Mean	SD
Control	UL	1.46	1.44	0.52	1.1	0.537029
	UR	1.65	3.21	1.43	2.1	0.970429
	LL	93.44	88.89	96.83	93.1	3.984098
	LR	3.46	6.46	1.23	3.7	2.62443
1	UL	1.36	3.19	0.24	1.6	1.489172
	UR	1.77	3.45	2.36	2.5	0.852311
	LL	93.53	85.76	95.67	91.7	5.214732
	LR	3.34	7.6	1.73	4.2	3.033057
3	UL	1.84	2.1	0.61	1.5	0.795885
	UR	2.71	4.29	2.25	3.1	1.070016
	LL	89.84	85.34	95.42	90.2	5.049634
	LR	5.62	8.27	1.72	5.2	3.294819

Table S10. *Continuation*

10	UL	3.51	2.17	1.49	2.4	1.027813
	UR	4.43	10.06	4.3	6.3	3.288652
	LL	84.86	74.97	90.37	83.4	7.803121
	LR	7.2	12.8	3.83	7.9	4.530964
33	UL	4.72	1.86	1.87	2.8	1.648343
	UR	6.64	10.75	9.92	9.1	2.1733
	LL	79.26	70.3	78.37	76.0	4.936237
	LR	9.38	17.08	9.84	12.1	4.318935
100	UL	5.39	2.26	2.25	3.3	1.81
	UR	11.26	13.17	10.53	11.7	1.363244
	LL	71.58	64.85	74.9	70.4	5.120511
	LR	11.77	19.72	12.33	14.6	4.43712

Concentration (nM)		Paclitaxel-induced apoptosis of SCC25 cell (%)				
		Sample 1	Sample 2	Sample 3	Mean	SD
Control	UL	2.18	0.37	0.35	0.97	1.050825
	UR	1.08	22.19	2.01	8.43	11.92846
	LL	94.41	74.71	95.46	88.19	11.68871
	LR	2.32	2.73	2.18	2.41	0.285832
1	UL	2.28	0.39	0.66	1.11	1.022204
	UR	1.93	18.92	3.61	8.15	9.361967
	LL	91.62	78.13	93.47	87.74	8.373751
	LR	4.18	2.56	2.26	3.00	1.03286
3	UL	2.4	0.35	1.36	1.37	1.025037
	UR	4.63	19.9	7.27	10.60	8.161489
	LL	83.66	74.68	87.09	81.81	6.408502
	LR	9.31	5.07	4.28	6.22	2.705014
10	UL	4.15	0.61	4.12	2.96	2.035215
	UR	13.09	26.89	18.08	19.35	6.987563
	LL	59.94	59.54	68.28	62.59	4.934626
	LR	22.82	12.96	9.52	15.10	6.903419
33	UL	4.97	0.4	4.26	3.21	2.459289
	UR	12.06	34.6	37.6	28.09	13.96032
	LL	61.62	35.36	46.77	47.92	13.1675
	LR	21.34	29.64	11.37	20.78	9.147712
100	UL	5.52	0.28	5.56	3.79	3.036928
	UR	15.29	38.17	54.76	36.07	19.81836
	LL	55.73	25.97	26.24	35.98	17.10453
	LR	23.47	35.58	13.44	24.16	11.08627

Table S11. Paclitaxel-induced apoptosis of Detroit 562 cells. Annexin V-FLUOS/PI (Ann/PI)-stained HNSCC cells were analyzed by FACS after 48 h of treatment with paclitaxel at different concentrations. Live cells are presented by the Ann-/PI- fraction (LL), apoptotic cells by the Ann+/PI- fraction (LR), secondary necrotic cells by the Ann+/PI+ fraction (UR) and primary necrotic cells are detected in the Ann-/PI+ fraction (UL). Statistical analysis was performed by Student's t-test, the cell fractions in all concentration were compared to control fractions in each cell line.
Red color indicate if $p < 0.05$. (Figure 3).

	T-test for Independent Samples (Paclitaxel_FACS_Detroit562) Note: Variables were treated as independent samples										
Group 1 vs. Group 2	Mean Group 1	Mean Group 2	t-value	df	p	Valid N Group 1	Valid N Group 2	Std.Dev. Group 1	Std.Dev. Group 2	F-ratio Variances	p Variances
Control UL vs. 1 nM UL	2.166667	1.750000	0.37671	4	0.725523	3	3	1.450149	1.251919	1.34176	0.854060
Control UL vs. 3 nM UL	2.166667	3.993333	-0.85408	4	0.441191	3	3	1.450149	3.408773	5.52549	0.306490
Control UL vs. 10 nM UL	2.166667	6.313333	-1.21689	4	0.290534	3	3	1.450149	5.721209	15.56504	0.120736
Control UL vs. 33 nM UL	2.166667	5.693333	-1.34776	4	0.249009	3	3	1.450149	4.293976	8.76786	0.204753
Control UL vs. 100 nM UL	2.166667	5.206667	-1.32641	4	0.255366	3	3	1.450149	3.695326	6.49352	0.266897
1 nM UL vs. 3 nM UL	1.750000	3.993333	-1.06999	4	0.344892	3	3	1.251919	3.408773	7.41385	0.237703
1 nM UL vs. 10 nM UL	1.750000	6.313333	-1.34958	4	0.248474	3	3	1.251919	5.721209	20.88447	0.091389
1 nM UL vs. 33 nM UL	1.750000	5.693333	-1.52704	4	0.201463	3	3	1.251919	4.293976	11.76433	0.156687
1 nM UL vs. 100 nM UL	1.750000	5.206667	-1.53452	4	0.199691	3	3	1.251919	3.695326	8.71271	0.205916
3 nM UL vs. 10 nM UL	3.993333	6.313333	-0.60338	4	0.578799	3	3	3.408773	5.721209	2.81695	0.523978
3 nM UL vs. 33 nM UL	3.993333	5.693333	-0.53707	4	0.619703	3	3	3.408773	4.293976	1.58680	0.773155
3 nM UL vs. 100 nM UL	3.993333	5.206667	-0.41802	4	0.697400	3	3	3.408773	3.695326	1.17519	0.919458
10 nM UL vs. 33 nM UL	6.313333	5.693333	0.15012	4	0.887934	3	3	5.721209	4.293976	1.77524	0.720659
10 nM UL vs. 100 nM UL	6.313333	5.206667	0.28143	4	0.792337	3	3	5.721209	3.695326	2.39701	0.588753
33 nM UL vs. 100 nM UL	5.693333	5.206667	0.14879	4	0.888917	3	3	4.293976	3.695326	1.35025	0.850974

Table S11. *Continuation.*

	T-test for Independent Samples (Paclitaxel_FACS_Detroit562) Note: Variables were treated as independent samples										
Group 1 vs. Group 2	Mean Group 1	Mean Group 2	t-value	df	p	Valid N Group 1	Valid N Group 2	Std.Dev. Group 1	Std.Dev. Group 2	F-ratio Variances	p Variances
Control UR vs. 100 nM UR	4.84000	18.18667	-1.90902	4	0.128891	3	3	2.50216	11.84808	22.42158	0.085391

Control UR vs. 33 nM UR	4.84000	18.79333	-2.11829	4	0.101537	3	3	2.50216	11.13136	19.79096	0.096196
Control UR vs. 10 nM UR	4.84000	15.54333	-3.29105	4	0.030186	3	3	2.50216	5.04684	4.06827	0.394612
Control UR vs. 3 nM UR	4.84000	8.02333	-0.71292	4	0.515277	3	3	2.50216	7.31799	8.55370	0.209343
Control UR vs. 1 nM UR	4.84000	5.36667	-0.21831	4	0.837877	3	3	2.50216	3.34665	1.78891	0.717125
1 nM UR vs. 100 nM UR	5.36667	18.18667	-1.80357	4	0.145633	3	3	3.34665	11.84808	12.53363	0.147780
1 nM UR vs. 33 nM UR	5.36667	18.79333	-2.00073	4	0.116019	3	3	3.34665	11.13136	11.06311	0.165795
1 nM UR vs. 10 nM UR	5.36667	15.54333	-2.91076	4	0.043645	3	3	3.34665	5.04684	2.27416	0.610844
1 nM UR vs. 3 nM UR	5.36667	8.02333	-0.57183	4	0.598037	3	3	3.34665	7.31799	4.78151	0.345931
3 nM UR vs. 100 nM UR	8.02333	18.18667	-1.26408	4	0.274846	3	3	7.31799	11.84808	2.62127	0.552292
3 nM UR vs. 33 nM UR	8.02333	18.79333	-1.40032	4	0.234013	3	3	7.31799	11.13136	2.31373	0.603550
3 nM UR vs. 10 nM UR	8.02333	15.54333	-1.46521	4	0.216731	3	3	7.31799	5.04684	2.10254	0.644633
10 nM UR vs. 100 nM UR	15.54333	18.18667	-0.35551	4	0.740159	3	3	5.04684	11.84808	5.51133	0.307157
10 nM UR vs. 33 nM UR	15.54333	18.79333	-0.46058	4	0.669030	3	3	5.04684	11.13136	4.86471	0.341023
33 nM UR vs. 100 nM UR	18.79333	18.18667	0.06464	4	0.951565	3	3	11.13136	11.84808	1.13292	0.937681

Table S11. *Continuation.*

Group 1 vs. Group 2	T-test for Independent Samples (Paclitaxel_FACS_Detroit562) Note: Variables were treated as independent samples										
	Mean Group 1	Mean Group 2	t-value	df	p	Valid N Group 1	Valid N Group 2	Std.Dev. Group 1	Std.Dev. Group 2	F-ratio Variances	p Variances
Control LL vs. 1 nM LL	87.32000	86.53333	0.14179	4	0.894098	3	3	6.08506	7.43723	1.493804	0.801988
Control LL vs. 3 nM LL	87.32000	79.93333	0.90995	4	0.414320	3	3	6.08506	12.67517	4.338891	0.374610
Control LL vs. 10 nM LL	87.32000	63.49333	2.97680	4	0.040868	3	3	6.08506	12.45670	4.190609	0.385311
Control LL vs. 33 nM LL	87.32000	59.48000	3.38384	4	0.027687	3	3	6.08506	12.88565	4.484184	0.364685
Control LL vs. 100 nM LL	87.32000	58.23000	3.92250	4	0.017211	3	3	6.08506	11.31247	3.456094	0.448824
1 nM LL vs. 3 nM LL	86.53333	79.93333	0.77787	4	0.480090	3	3	7.43723	12.67517	2.904592	0.512217
1 nM LL vs. 10 nM LL	86.53333	63.49333	2.75065	4	0.051340	3	3	7.43723	12.45670	2.805326	0.525579
1 nM LL vs. 33 nM LL	86.53333	59.48000	3.14948	4	0.034531	3	3	7.43723	12.88565	3.001855	0.499768
1 nM LL vs. 100 nM LL	86.53333	58.23000	3.62106	4	0.022336	3	3	7.43723	11.31247	2.313619	0.603570
3 nM LL vs. 10 nM LL	79.93333	63.49333	1.60227	4	0.184355	3	3	12.67517	12.45670	1.035385	0.982615
3 nM LL vs. 33 nM LL	79.93333	59.48000	1.95997	4	0.121558	3	3	12.67517	12.88565	1.033486	0.983533
3 nM LL vs. 100 nM LL	79.93333	58.23000	2.21266	4	0.091355	3	3	12.67517	11.31247	1.255432	0.886748

10 nM LL vs. 33 nM LL	63.49333	59.48000	0.38786	4	0.717878	3	3	12.45670	12.88565	1.070055	0.966158
10 nM LL vs. 100 nM LL	63.49333	58.23000	0.54178	4	0.616740	3	3	12.45670	11.31247	1.212527	0.903944
33 nM LL vs. 100 nM LL	59.48000	58.23000	0.12627	4	0.905613	3	3	12.88565	11.31247	1.297472	0.870522

Table S11. *Continuation.*

	T-test for Independent Samples (Paclitaxel_FACS_Detroit562) Note: Variables were treated as independent samples										
Group 1 vs. Group 2	Mean Group 1	Mean Group 2	t-value	df	p	Valid N Group 1	Valid N Group 2	Std.Dev. Group 1	Std.Dev. Group 2	F-ratio Variances	p Variances
Control LR vs. 1 nM LR	5.67000	6.35000	-0.15594	4	0.883631	3	3	5.52391	5.15064	1.150194	0.930149
Control LR vs. 3 nM LR	5.67000	8.05000	-0.48085	4	0.655739	3	3	5.52391	6.55591	1.408549	0.830375
Control LR vs. 10 nM LR	5.67000	14.65000	-1.81122	4	0.144343	3	3	5.52391	6.57507	1.416798	0.827541
Control LR vs. 33 nM LR	5.67000	16.03000	-1.83053	4	0.141141	3	3	5.52391	8.09807	2.149166	0.635089
Control LR vs. 100 nM LR	5.67000	18.37333	-1.84305	4	0.139105	3	3	5.52391	10.58339	3.670761	0.428196
1 nM LR vs. 3 nM LR	6.35000	8.05000	-0.35317	4	0.741784	3	3	5.15064	6.55591	1.620104	0.763328
1 nM LR vs. 10 nM LR	6.35000	14.65000	-1.72121	4	0.160323	3	3	5.15064	6.57507	1.629592	0.760574
1 nM LR vs. 33 nM LR	6.35000	16.03000	-1.74698	4	0.155564	3	3	5.15064	8.09807	2.471957	0.576044
1 nM LR vs. 100 nM LR	6.35000	18.37333	-1.76930	4	0.151563	3	3	5.15064	10.58339	4.222086	0.382989
3 nM LR vs. 10 nM LR	8.05000	14.65000	-1.23118	4	0.285695	3	3	6.55591	6.57507	1.005856	0.997080
3 nM LR vs. 33 nM LR	8.05000	16.03000	-1.32657	4	0.255318	3	3	6.55591	8.09807	1.525802	0.791828
3 nM LR vs. 100 nM LR	8.05000	18.37333	-1.43625	4	0.224278	3	3	6.55591	10.58339	2.606058	0.554622
10 nM LR vs. 33 nM LR	14.65000	16.03000	-0.22914	4	0.829998	3	3	6.57507	8.09807	1.516918	0.794623
10 nM LR vs. 100 nM LR	14.65000	18.37333	-0.51760	4	0.632048	3	3	6.57507	10.58339	2.590886	0.556966
33 nM LR vs. 100 nM LR	16.03000	18.37333	-0.30457	4	0.775881	3	3	8.09807	10.58339	1.707993	0.738554

Table S12. Paclitaxel-induced apoptosis of FaDu cells. Annexin V-FLUOS/PI (Ann/PI)-stained HNSCC cells were analyzed by FACS after 48 h of treatment with paclitaxel at different concentrations. Live cells are presented by the Ann-/PI- fraction (LL), apoptotic cells by the Ann+/PI- fraction (LR), secondary necrotic cells by the Ann+/PI+ fraction (UR) and primary necrotic cells are detected in the Ann-/PI+ fraction (UL). Statistical analysis was performed by Student's t-test, the cell fractions in all concentration were compared to control fractions in each cell line.
Red color indicate if $p < 0.05$. (Figure 3).

	T-test for Independent Samples (Paclitaxel_FACS_FaDu) Note: Variables were treated as independent samples

Group 1 vs. Group 2	Mean Group 1	Mean Group 2	t-value	df	p	Valid N Group 1	Valid N Group 2	Std.Dev. Group 1	Std.Dev. Group 2	F-ratio Variances	p Variances
Control UL vs. 1 nM UL	1.140000	1.596667	-0.49965	4	0.643555	3	3	0.537029	1.489172	7.68944	0.230165
Control UL vs. 3 nM UL	1.140000	1.516667	-0.67950	4	0.534107	3	3	0.537029	0.795885	2.19637	0.625710
Control UL vs. 10 nM UL	1.140000	2.390000	-1.86699	4	0.135302	3	3	0.537029	1.027813	3.66297	0.428911
Control UL vs. 33 nM UL	1.140000	2.816667	-1.67515	4	0.169215	3	3	0.537029	1.648343	9.42106	0.191919
Control UL vs. 100 nM UL	1.140000	3.300000	-1.98160	4	0.118585	3	3	0.537029	1.810000	11.35957	0.161818
1 nM UL vs. 3 nM UL	1.596667	1.516667	0.08206	4	0.938539	3	3	1.489172	0.795885	3.50097	0.444348
1 nM UL vs. 10 nM UL	1.596667	2.390000	-0.75941	4	0.489901	3	3	1.489172	1.027813	2.09924	0.645320
1 nM UL vs. 33 nM UL	1.596667	2.816667	-0.95124	4	0.395347	3	3	1.489172	1.648343	1.22520	0.898798
1 nM UL vs. 100 nM UL	1.596667	3.300000	-1.25871	4	0.276588	3	3	1.489172	1.810000	1.47730	0.807332
3 nM UL vs. 10 nM UL	1.516667	2.390000	-1.16364	4	0.309250	3	3	0.795885	1.027813	1.66774	0.749699
3 nM UL vs. 33 nM UL	1.516667	2.816667	-1.23013	4	0.286047	3	3	0.795885	1.648343	4.28938	0.378116
3 nM UL vs. 100 nM UL	1.516667	3.300000	-1.56218	4	0.193277	3	3	0.795885	1.810000	5.17197	0.324045
10 nM UL vs. 33 nM UL	2.390000	2.816667	-0.38044	4	0.722962	3	3	1.027813	1.648343	2.57197	0.559914
10 nM UL vs. 100 nM UL	2.390000	3.300000	-0.75724	4	0.491064	3	3	1.027813	1.810000	3.10119	0.487663
33 nM UL vs. 100 nM UL	2.816667	3.300000	-0.34196	4	0.749589	3	3	1.648343	1.810000	1.20576	0.906715

Table S12. *Continuation.*

Group 1 vs. Group 2	T-test for Independent Samples (Paclitaxel_FACS_FaDu) Note: Variables were treated as independent samples										
	Mean Group 1	Mean Group 2	t-value	df	p	Valid N Group 1	Valid N Group 2	Std.Dev. Group 1	Std.Dev. Group 2	F-ratio Variances	p Variances
Control UR vs. 1 nM UR	2.09667	2.52667	-0.57665	4	0.595075	3	3	0.970429	0.852311	1.29638	0.870936
Control UR vs. 3 nM UR	2.09667	3.08333	-1.18305	4	0.302299	3	3	0.970429	1.070016	1.21577	0.902620
Control UR vs. 10 nM UR	2.09667	6.26333	-2.10476	4	0.103098	3	3	0.970429	3.288652	11.48439	0.160200
Control UR vs. 33 nM UR	2.09667	9.10333	-5.09886	4	0.006988	3	3	0.970429	2.173300	5.01547	0.332476
Control UR vs. 100 nM UR	2.09667	11.65333	-9.89180	4	0.000586	3	3	0.970429	1.363244	1.97342	0.672627
1 nM UR vs. 3 nM UR	2.52667	3.08333	-0.70482	4	0.519799	3	3	0.852311	1.070016	1.57610	0.776367
1 nM UR vs. 10 nM UR	2.52667	6.26333	-1.90507	4	0.129480	3	3	0.852311	3.288652	14.88813	0.125880
1 nM UR vs. 33 nM UR	2.52667	9.10333	-4.87957	4	0.008163	3	3	0.852311	2.173300	6.50195	0.266597

1 nM UR vs. 100 nM UR	2.52667	11.65333	-9.83227	4	0.000600	3	3	0.852311	1.363244	2.55830	0.562066
3 nM UR vs. 10 nM UR	3.08333	6.26333	-1.59265	4	0.186458	3	3	1.070016	3.288652	9.44617	0.191458
3 nM UR vs. 33 nM UR	3.08333	9.10333	-4.30433	4	0.012602	3	3	1.070016	2.173300	4.12533	0.390218
3 nM UR vs. 100 nM UR	3.08333	11.65333	-8.56520	4	0.001020	3	3	1.070016	1.363244	1.62318	0.762433
10 nM UR vs. 33 nM UR	6.26333	9.10333	-1.24789	4	0.280136	3	3	3.288652	2.173300	2.28979	0.607941
10 nM UR vs. 100 nM UR	6.26333	11.65333	-2.62240	4	0.058652	3	3	3.288652	1.363244	5.81954	0.293275
33 nM UR vs. 100 nM UR	9.10333	11.65333	-1.72160	4	0.160249	3	3	2.173300	1.363244	2.54151	0.564730

Table S12. *Continuation.*

Group 1 vs. Group 2	T-test for Independent Samples (Paclitaxel_FACS_FaDu) Note: Variables were treated as independent samples										
	Mean Group 1	Mean Group 2	t-value	df	p	Valid N Group 1	Valid N Group 2	Std.Dev. Group 1	Std.Dev. Group 2	F-ratio Variances	p Variances
Control LL vs. 1 nM LL	93.05333	91.65333	0.36950	4	0.730482	3	3	3.984098	5.214732	1.713184	0.737141
Control LL vs. 3 nM LL	93.05333	90.20000	0.76835	4	0.485128	3	3	3.984098	5.049634	1.606423	0.767335
Control LL vs. 10 nM LL	93.05333	83.40000	1.90838	4	0.128986	3	3	3.984098	7.803121	3.835984	0.413566
Control LL vs. 33 nM LL	93.05333	75.97667	4.66270	4	0.009570	3	3	3.984098	4.936237	1.535084	0.788929
Control LL vs. 100 nM LL	93.05333	70.44333	6.03612	4	0.003798	3	3	3.984098	5.120511	1.651835	0.754195
1 nM LL vs. 3 nM LL	91.65333	90.20000	0.34678	4	0.746233	3	3	5.214732	5.049634	1.066459	0.967839
1 nM LL vs. 10 nM LL	91.65333	83.40000	1.52316	4	0.202386	3	3	5.214732	7.803121	2.239096	0.617456
1 nM LL vs. 33 nM LL	91.65333	75.97667	3.78145	4	0.019411	3	3	5.214732	4.936237	1.116020	0.945171
1 nM LL vs. 100 nM LL	91.65333	70.44333	5.02664	4	0.007351	3	3	5.214732	5.120511	1.037140	0.981769
3 nM LL vs. 10 nM LL	90.20000	83.40000	1.26720	4	0.273838	3	3	5.049634	7.803121	2.387905	0.590335
3 nM LL vs. 33 nM LL	90.20000	75.97667	3.48870	4	0.025153	3	3	5.049634	4.936237	1.046472	0.977291
3 nM LL vs. 100 nM LL	90.20000	70.44333	4.75830	4	0.008916	3	3	5.049634	5.120511	1.028269	0.986062
10 nM LL vs. 33 nM LL	83.40000	75.97667	1.39251	4	0.236182	3	3	7.803121	4.936237	2.498876	0.571612
10 nM LL vs. 100 nM LL	83.40000	70.44333	2.40450	4	0.073995	3	3	7.803121	5.120511	2.322256	0.602001
33 nM LL vs. 100 nM LL	75.97667	70.44333	1.34751	4	0.249083	3	3	4.936237	5.120511	1.076055	0.963365

Table S12. *Continuation.*

Group 1 vs. Group 2	T-test for Independent Samples (Paclitaxel_FACS_FaDu) Note: Variables were treated as independent samples										
	Mean Group 1	Mean Group 2	t-value	df	p	Valid N Group 1	Valid N Group 2	Std.Dev. Group 1	Std.Dev. Group 2	F-ratio Variances	p Variances
Control LR vs. 1 nM LR	3.71667	4.22333	-0.21880	4	0.837517	3	3	2.624430	3.033057	1.335645	0.856294
Control LR vs. 3 nM LR	3.71667	5.20333	-0.61130	4	0.574035	3	3	2.624430	3.294819	1.576134	0.776357
Control LR vs. 10 nM LR	3.71667	7.94333	-1.39813	4	0.234620	3	3	2.624430	4.530964	2.980651	0.502430
Control LR vs. 33 nM LR	3.71667	12.10000	-2.87316	4	0.045324	3	3	2.624430	4.318935	2.708216	0.539343
Control LR vs. 100 nM LR	3.71667	14.60667	-3.65887	4	0.021602	3	3	2.624430	4.437120	2.858461	0.518341
1 nM LR vs. 3 nM LR	4.22333	5.20333	-0.37903	4	0.723927	3	3	3.033057	3.294819	1.180055	0.917408
1 nM LR vs. 10 nM LR	4.22333	7.94333	-1.18172	4	0.302773	3	3	3.033057	4.530964	2.231619	0.618885
1 nM LR vs. 33 nM LR	4.22333	12.10000	-2.58506	4	0.061001	3	3	3.033057	4.318935	2.027647	0.660579
1 nM LR vs. 100 nM LR	4.22333	14.60667	-3.34613	4	0.028672	3	3	3.033057	4.437120	2.140135	0.636915
3 nM LR vs. 10 nM LR	5.20333	7.94333	-0.84712	4	0.444636	3	3	3.294819	4.530964	1.891115	0.691775
3 nM LR vs. 33 nM LR	5.20333	12.10000	-2.19899	4	0.092758	3	3	3.294819	4.318935	1.718265	0.735763
3 nM LR vs. 100 nM LR	5.20333	14.60667	-2.94700	4	0.042095	3	3	3.294819	4.437120	1.813590	0.710836
10 nM LR vs. 33 nM LR	7.94333	12.10000	-1.15016	4	0.314163	3	3	4.530964	4.318935	1.100596	0.952111
10 nM LR vs. 100 nM LR	7.94333	14.60667	-1.81988	4	0.142896	3	3	4.530964	4.437120	1.042747	0.979074
33 nM LR vs. 100 nM LR	12.10000	14.60667	-0.70117	4	0.521842	3	3	4.318935	4.437120	1.055478	0.973010

Table S13. Paclitaxel-induced apoptosis of SCC25 cells. Annexin V-FLUOS/PI (Ann/PI)-stained HNSCC cells were analyzed by FACS after 48 h of treatment with paclitaxel at different concentrations. Live cells are presented by the Ann-/PI- fraction (LL), apoptotic cells by the Ann+/PI- fraction (LR), secondary necrotic cells by the Ann+/PI+ fraction (UR) and primary necrotic cells are detected in the Ann-/PI+ fraction (UL). Statistical analysis was performed by Student's t-test, the cell fractions in all concentration were compared to control fractions in each cell line.
Red color indicate if $p < 0.05$. (Figure 3).

Group 1 vs. Group 2	T-test for Independent Samples (Paclitaxel_FACS_SCC25) Note: Variables were treated as independent samples										
	Mean Group 1	Mean Group 2	t-value	df	p	Valid N Group 1	Valid N Group 2	Std.Dev. Group 1	Std.Dev. Group 2	F-ratio Variances	p Variances
Control UL vs. 1 nM UL	0.966667	1.110000	-0.16935	4	0.873743	3	3	1.050825	1.022204	1.056784	0.972392
Control UL vs. 3 nM UL	0.966667	1.370000	-0.47589	4	0.658978	3	3	1.050825	1.025037	1.050950	0.975158
Control UL vs. 10 nM UL	0.966667	2.960000	-1.50734	4	0.206203	3	3	1.050825	2.035215	3.751109	0.420954

Control UL vs. 33 nM UL	0.966667	3.210000	-1.45288	4	0.219912	3	3	1.050825	2.459289	5.477194	0.308776
Control UL vs. 100 nM UL	0.966667	3.786667	-1.51991	4	0.203164	3	3	1.050825	3.036928	8.352341	0.213850
1 nM UL vs. 3 nM UL	1.110000	1.370000	-0.31109	4	0.771273	3	3	1.022204	1.025037	1.005551	0.997232
1 nM UL vs. 10 nM UL	1.110000	2.960000	-1.40694	4	0.232189	3	3	1.022204	2.035215	3.964111	0.402892
1 nM UL vs. 33 nM UL	1.110000	3.210000	-1.36573	4	0.243778	3	3	1.022204	2.459289	5.788209	0.294629
1 nM UL vs. 100 nM UL	1.110000	3.786667	-1.44682	4	0.221493	3	3	1.022204	3.036928	8.826618	0.203529
3 nM UL vs. 10 nM UL	1.370000	2.960000	-1.20853	4	0.293400	3	3	1.025037	2.035215	3.942229	0.404676
3 nM UL vs. 33 nM UL	1.370000	3.210000	-1.19615	4	0.297693	3	3	1.025037	2.459289	5.756258	0.296022
3 nM UL vs. 100 nM UL	1.370000	3.786667	-1.30592	4	0.261619	3	3	1.025037	3.036928	8.777894	0.204543
10 nM UL vs. 33 nM UL	2.960000	3.210000	-0.13565	4	0.898653	3	3	2.035215	2.459289	1.460153	0.812958
10 nM UL vs. 100 nM UL	2.960000	3.786667	-0.39166	4	0.715281	3	3	2.035215	3.036928	2.226632	0.619841
33 nM UL vs. 100 nM UL	3.210000	3.786667	-0.25559	4	0.810869	3	3	2.459289	3.036928	1.524931	0.792101

Table S13. *Continuation.*

	T-test for Independent Samples (Paclitaxel_FACS_SCC25) Note: Variables were treated as independent samples										
Group 1 vs. Group 2	Mean Group 1	Mean Group 2	t-value	df	p	Valid N Group 1	Valid N Group 2	Std.Dev. Group 1	Std.Dev. Group 2	F-ratio Variances	p Variances
Control UR vs. 1 nM UR	8.42667	8.15333	0.03122	4	0.976589	3	3	11.92846	9.36197	1.623434	0.762359
Control UR vs. 3 nM UR	8.42667	10.60000	-0.26045	4	0.807377	3	3	11.92846	8.16149	2.136142	0.637726
Control UR vs. 10 nM UR	8.42667	19.35333	-1.36899	4	0.242839	3	3	11.92846	6.98756	2.914188	0.510962
Control UR vs. 33 nM UR	8.42667	28.08667	-1.85445	4	0.137281	3	3	11.92846	13.96032	1.369688	0.843993
Control UR vs. 100 nM UR	8.42667	36.07333	-2.07016	4	0.107211	3	3	11.92846	19.81836	2.760363	0.531863
1 nM UR vs. 3 nM UR	8.15333	10.60000	-0.34120	4	0.750120	3	3	9.36197	8.16149	1.315817	0.863626
1 nM UR vs. 10 nM UR	8.15333	19.35333	-1.66057	4	0.172138	3	3	9.36197	6.98756	1.795076	0.715544
1 nM UR vs. 33 nM UR	8.15333	28.08667	-2.05401	4	0.109192	3	3	9.36197	13.96032	2.223599	0.620425
1 nM UR vs. 100 nM UR	8.15333	36.07333	-2.20632	4	0.092002	3	3	9.36197	19.81836	4.481269	0.364879
3 nM UR vs. 10 nM UR	10.60000	19.35333	-1.41112	4	0.231043	3	3	8.16149	6.98756	1.364229	0.845942
3 nM UR vs. 33 nM UR	10.60000	28.08667	-1.87297	4	0.134368	3	3	8.16149	13.96032	2.925849	0.509444
3 nM UR vs. 100 nM UR	10.60000	36.07333	-2.05855	4	0.108631	3	3	8.16149	19.81836	5.896529	0.290001

10 nM UR vs. 33 nM UR	19.35333	28.08667	-0.96894	4	0.387442	3	3	6.98756	13.96032	3.991529	0.400679
10 nM UR vs. 100 nM UR	19.35333	36.07333	-1.37812	4	0.240236	3	3	6.98756	19.81836	8.044218	0.221136
33 nM UR vs. 100 nM UR	28.08667	36.07333	-0.57064	4	0.598770	3	3	13.96032	19.81836	2.015322	0.663279

Table S13. *Continuation.*

Group 1 vs. Group 2	T-test for Independent Samples (Paclitaxel_FACS_SCC25) Note: Variables were treated as independent samples										
	Mean Group 1	Mean Group 2	t-value	df	p	Valid N Group 1	Valid N Group 2	Std.Dev. Group 1	Std.Dev. Group 2	F-ratio Variances	p Variances
Control LL vs. 1 nM LL	88.19333	87.74000	0.05461	4	0.959069	3	3	11.68871	8.37375	1.94847	0.678319
Control LL vs. 3 nM LL	88.19333	81.81000	0.82941	4	0.453499	3	3	11.68871	6.40850	3.32675	0.462241
Control LL vs. 10 nM LL	88.19333	62.58667	3.49569	4	0.024994	3	3	11.68871	4.93463	5.61079	0.302536
Control LL vs. 33 nM LL	88.19333	47.91667	3.96212	4	0.016648	3	3	11.68871	13.16750	1.26904	0.881432
Control LL vs. 100 nM LL	88.19333	35.98000	4.36533	4	0.012012	3	3	11.68871	17.10453	2.14136	0.636667
1 nM LL vs. 3 nM LL	87.74000	81.81000	0.97406	4	0.385183	3	3	8.37375	6.40850	1.70737	0.738725
1 nM LL vs. 10 nM LL	87.74000	62.58667	4.48238	4	0.010970	3	3	8.37375	4.93463	2.87960	0.515518
1 nM LL vs. 33 nM LL	87.74000	47.91667	4.42024	4	0.011509	3	3	8.37375	13.16750	2.47267	0.575925
1 nM LL vs. 100 nM LL	87.74000	35.98000	4.70750	4	0.009257	3	3	8.37375	17.10453	4.17237	0.386670
3 nM LL vs. 10 nM LL	81.81000	62.58667	4.11657	4	0.014653	3	3	6.40850	4.93463	1.68657	0.744443
3 nM LL vs. 33 nM LL	81.81000	47.91667	4.00876	4	0.016013	3	3	6.40850	13.16750	4.22176	0.383013
3 nM LL vs. 100 nM LL	81.81000	35.98000	4.34586	4	0.012197	3	3	6.40850	17.10453	7.12376	0.246191
10 nM LL vs. 33 nM LL	62.58667	47.91667	1.80697	4	0.145058	3	3	4.93463	13.16750	7.12030	0.246296
10 nM LL vs. 100 nM LL	62.58667	35.98000	2.58869	4	0.060768	3	3	4.93463	17.10453	12.01473	0.153672
33 nM LL vs. 100 nM LL	47.91667	35.98000	0.95780	4	0.392403	3	3	13.16750	17.10453	1.68739	0.744216

Table 13. *Continuation.*

Group 1 vs. Group 2	T-test for Independent Samples (Paclitaxel_FACS_SCC25) Note: Variables were treated as independent samples										
	Mean Group 1	Mean Group 2	t-value	df	p	Valid N Group 1	Valid N Group 2	Std.Dev. Group 1	Std.Dev. Group 2	F-ratio Variances	p Variances
Control LR vs. 1 nM LR	2.41000	3.00000	-0.95356	4	0.394305	3	3	0.28583	1.03286	13.058	0.142273
Control LR vs. 3 nM LR	2.41000	6.22000	-2.42608	4	0.072286	3	3	0.28583	2.70501	89.561	0.022085

Control LR vs. 10 nM LR	2.41000	15.10000	-3.18116	4	0.033498	3	3	0.28583	6.90342	583.319	0.003423
Control LR vs. 33 nM LR	2.41000	20.78333	-3.47716	4	0.025418	3	3	0.28583	9.14771	1024.243	0.001951
Control LR vs. 100 nM LR	2.41000	24.16333	-3.39748	4	0.027341	3	3	0.28583	11.08627	1504.350	0.001329
1 nM LR vs. 3 nM LR	3.00000	6.22000	-1.92616	4	0.126372	3	3	1.03286	2.70501	6.859	0.254488
1 nM LR vs. 10 nM LR	3.00000	15.10000	-3.00244	4	0.039846	3	3	1.03286	6.90342	44.673	0.043790
1 nM LR vs. 33 nM LR	3.00000	20.78333	-3.34588	4	0.028678	3	3	1.03286	9.14771	78.441	0.025176
1 nM LR vs. 100 nM LR	3.00000	24.16333	-3.29217	4	0.030154	3	3	1.03286	11.08627	115.209	0.017210
3 nM LR vs. 10 nM LR	6.22000	15.10000	-2.07441	4	0.106696	3	3	2.70501	6.90342	6.513	0.266201
3 nM LR vs. 33 nM LR	6.22000	20.78333	-2.64427	4	0.057324	3	3	2.70501	9.14771	11.436	0.160819
3 nM LR vs. 100 nM LR	6.22000	24.16333	-2.72346	4	0.052798	3	3	2.70501	11.08627	16.797	0.112378
10 nM LR vs. 33 nM LR	15.10000	20.78333	-0.85895	4	0.438794	3	3	6.90342	9.14771	1.756	0.725719
10 nM LR vs. 100 nM LR	15.10000	24.16333	-1.20201	4	0.295655	3	3	6.90342	11.08627	2.579	0.558823
33 nM LR vs. 100 nM LR	20.78333	24.16333	-0.40731	4	0.704634	3	3	9.14771	11.08627	1.469	0.810129

Table S14. Changes in the levels of Cx43 and Bcl-2 after *GJA1* siRNA knockdown in HNSCC cell lines. Densitometry analysis was performed using three independent experiments. The expression of all proteins were compared to expression of the non-targeting siRNA treated negative controls, after normalization to α-tubulin. (Figure 4C).

Cell line	non-targ. siRNA	*GJA1* siRNA	Densitometry intensity of Cx43				
			Sample1	Sample2	Sample3	Mean	SD
Detroit 562	-	+	2033.376	14413.075	24682.217	13709.56	11340.80
	+	-	27350.489	36386.229	46415.735	36717.48	9536.94
FaDu	-	+	238.536	1096.506	484.243	606.43	441.84
	+	-	18180.175	17670.468	23580.338	19810.33	3274.86
SCC25	-	+	225.95	153.243	271.657	216.95	59.72
	+	-	46966.037	52617.38	39347.702	46310.37	6659.09

Cell line	non-targ. siRNA	*GJA1* siRNA	Densitometry intensity ratio of Cx43				
			Sample1	Sample2	Sample3	Mean	SD
Detroit 562	-	+	0.07	0.40	0.53	0.33	0.23
	+	-	1.00	1.00	1.00	1.00	0.00
FaDu	-	+	0.01	0.06	0.02	0.03	0.03
	+	-	1.00	1.00	1.00	1.00	0.00
SCC25	-	+	0.005	0.003	0.007	0.005	0.002
	+	-	1.00	1.00	1.00	1.00	0.00

Cell line	non-targ. siRNA	*GJA1* siRNA	Densitometry intensity of Bcl-2				
			Sample1	Sample2	Sample3	Mean	SD
Detroit 562	-	+	39833.158	26670.439	54567.765	40357.12	13956.04
	+	-	47683.087	32929.752	55872.451	45495.10	11626.79
FaDu	-	+	46459.451	20580.246	39330.894	35456.86	13367.48
	+	-	47085.693	22408.539	41324.48	36939.57	12909.73
SCC25	-	+	36417.706	3267.276	38347.643	26010.88	19720.16
	+	-	19374.037	1843.255	22078.865	14432.05	10985.78

Cell line	non-targ. siRNA	*GJA1* siRNA	Densitometry intensity ratio of Bcl-2				
			Sample1	Sample2	Sample3	Mean	SD
Detroit 562	-	+	0.84	0.81	0.98	0.87	0.09
	+	-	1.00	1.00	1.00	1.00	0.00
FaDu	-	+	0.99	0.92	0.95	0.95	0.03
	+	-	1.00	1.00	1.00	1.00	0.00
SCC25	-	+	1.88	1.77	1.74	1.80	0.07

	+	-	1.00	1.00	1.00	1.00	0.00

Cell line	non-targ. siRNA	*GJA1* siRNA	Densitometry intensity of α-tubulin				
			Sample1	Sample2	Sample3	Mean	SD
Detroit 562	-	+	38601.622	81183.655	42655.288	54146.86	23502.12
	+	-	35977.116	45325.2	45023.409	42108.58	5312.14
FaDu	-	+	57659.966	63378.676	68391.2	63143.28	5369.49
	+	-	59575.35	62569.856	65162.886	62436.03	2796.17
SCC25	-	+	44299.773	53958.815	45026.48	47761.69	5379.15
	+	-	47016.238	52371.915	46102.066	48496.74	3386.98

Table S14. *Continuation.*

Cell line	non-targ. siRNA	*GJA1* siRNA	Densitometry intensity ratio of α-tubulin				
			Sample1	Sample2	Sample3	Mean	SD
Detroit 562	-	+	1.07	1.79	0.95	1.27	0.46
	+	-	1.00	1.00	1.00	1.00	0.00
FaDu	-	+	0.97	1.01	1.05	1.01	0.04
	+	-	1.00	1.00	1.00	1.00	0.00
SCC25	-	+	0.94	1.03	0.98	0.98	0.04
	+	-	1.00	1.00	1.00	1.00	0.00

Cell line	non-targ. siRNA	*GJA1* siRNA	Densitometry intensity ratio of Cx43 after normalization to α-tubulin				
			Sample1	Sample2	Sample3	Mean	SD
Detroit 562	-	+	0.07	0.22	0.56	0.28	0.25
	+	-	1.00	1.00	1.00	1.00	0.00
FaDu	-	+	0.01	0.06	0.02	0.03	0.03
	+	-	1.00	1.00	1.00	1.00	0.00
SCC25	-	+	0.01	0.003	0.01	0.01	0.002
	+	-	1.00	1.00	1.00	1.00	0.00

Cell line	non-targ. siRNA	*GJA1* siRNA	Densitometry intensity ratio of Bcl-2 after normalization to α-tubulin				
			Sample1	Sample2	Sample3	Mean	SD
Detroit 562	-	+	0.78	0.45	1.03	0.75	0.29
	+	-	1.00	1.00	1.00	1.00	0.00
FaDu	-	+	1.02	0.91	0.91	0.94	0.07
	+	-	1.00	1.00	1.00	1.00	0.00
SCC25	-	+	1.99	1.72	1.78	1.83	0.14

+	-	1.00	1.00	1.00	1.00	0.00

Table S15. Changes in the levels of Cx43 and Bcl-2 after *GJA1* siRNA knockdown in HNSCC cell lines. Statistical analysis was performed by Student's t-test, in each cell line the expression of all proteins in *GJA1* siRNA treated samples were compared to protein expression in non-targeting siRNA treated samples. Red color indicate if $p < 0.05$. (Figure 4C).

	T-test for Independent Samples (Cx43. Bcl2 siRNA) Note: Variables were treated as independent samples									
Group 1 vs. Group 2	Mean Group 1	Mean Group 2	t-value	df	p	N 1	N 2	Std.Dev. Group 1	Std.Dev. Group 2	F-ratio Variances	p Variances
Detroit 562 siCx43 Cx43/tubulin vs. Detroit 562 siControl Cx43/tubulin	0.283333	1.000000	-4.94416	4	0.007794	3	3	0.251064	0.00	0.00	1.000000

	T-test for Independent Samples (Cx43. Bcl2 siRNA) Note: Variables were treated as independent samples										
Group 1 vs. Group 2	Mean Group 1	Mean Group 2	t-value	df	p	N 1	N 2	Std.Dev. Group 1	Std.Dev. Group 2	F-ratio Variances	p Variances
Detroit 562 siCx43 Bcl2/tubulin vs. Detroit 562 siControl Bcl2/tubulin	0.753333	1.000000	-1.46859	4	0.215867	3	3	0.290918	0.00	0.00	1.000000

	T-test for Independent Samples (Cx43. Bcl2 siRNA) Note: Variables were treated as independent samples										
Group 1 vs. Group 2	Mean Group 1	Mean Group 2	t-value	df	p	N 1	N 2	Std.Dev. Group 1	Std.Dev. Group 2	F-ratio Variances	p Variances
FaDu siCx43 Cx43/tubulin vs. FaDu siControl Cx43/tubulin	0.030000	1.000000	-63.5014	4	0.000000	3	3	0.026458	0.00	0.00	1.000000

	T-test for Independent Samples (Cx43. Bcl2 siRNA) Note: Variables were treated as independent samples										
Group 1 vs. Group 2	Mean Group 1	Mean Group 2	t-value	df	p	N 1	N 2	Std.Dev. Group 1	Std.Dev. Group 2	F-ratio Variances	p Variances
FaDu siCx43 Bcl2/tubulin vs. FaDu siControl Bcl2/tubulin	0.946667	1.000000	-1.45455	4	0.219480	3	3	0.063509	0.00	0.00	1.000000

	T-test for Independent Samples (Cx43. Bcl2 siRNA) Note: Variables were treated as independent samples										
Group 1 vs. Group 2	Mean Group 1	Mean Group 2	t-value	df	p	N 1	N 2	Std.Dev. Group 1	Std.Dev. Group 2	F-ratio Variances	p Variances
SCC25 siCx43 Cx43/tubulin vs. SCC25 siControl Cx43/tubulin	0.007667	1.000000	-425.286	4	0.000000	3	3	0.004041	0.00	0.00	1.000000

	T-test for Independent Samples (Cx43. Bcl2 siRNA) Note: Variables were treated as independent samples										
Group 1 vs. Group 2	Mean Group 1	Mean Group 2	t-value	df	p	N 1	N 2	Std.Dev. Group 1	Std.Dev. Group 2	F-ratio Variances	p Variances
SCC25 siCx43 Bcl2/tubulin vs. SCC25 siControl Bcl2/tubulin	1.830000	1.000000	10.14006	4	0.000533	3	3	0.141774	0.00	0.00	1.000000

Table S16. Changes in the levels of Cx43 and Bcl-2 after Cx43 plasmid transfection in HNSCC cell lines. Densitometry analysis was performed using three independent experiments. The expression of all proteins were compared to expression of the the control plasmid treated controls, after normalization to α-tubulin. (Figure 4C).

Cell line	control plasmid	Cx43 plasmid	Densitometry intensity of Cx43				
			Sample1	Sample2	Sample3	Mean	SD

Detroit 562	+	-	12927.246	17727.773	443.192	10366.07	8922.38
	-	+	15706.459	40611.016	1401.355	19239.61	19842.17
FaDu	+	-	7167.296	3659.983	1599.335	4142.20	2815.13
	-	+	46062.057	85854.182	28102.643	53339.63	29555.58
SCC25	+	-	83349.141	21344.338	15950.116	40214.53	37452.91
	-	+	89663.534	44354.551	33706.794	55908.29	29713.73

Cell line	control plasmid	Cx43 plasmid	Densitometry intensity ratio of Cx43				
			Sample1	Sample2	Sample3	Mean	SD
Detroit 562	+	-	1.00	1.00	1.00	1.00	0.00
	-	+	1.21	2.29	3.16	2.22	0.98
FaDu	+	-	1.00	1.00	1.00	1.00	0.00
	-	+	6.43	23.46	17.57	15.82	8.65
SCC25	+	-	1.00	1.00	1.00	1.00	0.00
	-	+	1.08	2.08	2.11	1.76	0.59

Cell line	control plasmid	Cx43 plasmid	Densitometry intensity of Bcl-2				
			Sample1	Sample2	Sample3	Mean	SD
Detroit 562	+	-	26180.673	63103.208	27522.995	38935.63	20940.50
	-	+	17938.681	58450.673	34458.288	36949.21	20370.54
FaDu	+	-	34953.765	12093.518	17825.752	21624.35	11894.10
	-	+	18042.602	7312.075	12910.288	12754.99	5366.95
SCC25	+	-	11264.903	36610.459	7610.953	18495.44	15794.09
	-	+	11998.568	22999.581	11714.731	15570.96	6434.94

Cell line	control plasmid	Cx43 plasmid	Densitometry intensity ratio of Bcl-2				
			Sample1	Sample2	Sample3	Mean	SD
Detroit 562	+	-	1.00	1.00	1.00	1.00	0.00
	-	+	0.69	0.93	1.25	0.95	0.28
FaDu	+	-	1.00	1.00	1.00	1.00	0.00
	-	+	0.52	0.60	0.72	0.62	0.10
SCC25	+	-	1.00	1.00	1.00	1.00	0.00
	-	+	1.07	0.63	1.54	1.08	0.46

Cell line	control plasmid	Cx43 plasmid	Densitometry intensity of α-tubulin				
			Sample1	Sample2	Sample3	Mean	SD
Detroit 562	+	-	42183.43	45450.995	25165.51	37599.98	10891.80
	-	+	42463.38	52334.602	32299.137	42365.71	10018.09
FaDu	+	-	55866.522	39057.673	32829.359	42584.52	11916.66

	-	+	52392.551	39973.492	43844.108	45403.38	6354.66
SCC25	+	-	72646.836	40715.401	45867.016	53076.42	17143.10
	-	+	76718.22	32498.602	67022.543	58746.46	23242.50

Table S16. *Continuation.*

Cell line	control plasmid	Cx43 plasmid	Densitometry intensity ratio of α-tubulin				
			Sample1	Sample2	Sample3	Mean	SD
Detroit 562	+	-	1.00	1.00	1.00	1.00	0.00
	-	+	1.01	1.15	1.28	1.15	0.14
FaDu	+	-	1.00	1.00	1.00	1.00	0.00
	-	+	0.94	1.02	1.34	1.10	0.21
SCC25	+	-	1.00	1.00	1.00	1.00	0.00
	-	+	1.06	0.80	1.46	1.11	0.33
**Cell line**	**control plasmid**	**Cx43 plasmid**	**Densitometry intensity ratio of Cx43 after normalization to α-tubulin**				
			Sample1	Sample2	Sample3	Mean	SD
Detroit 562	+	-	1.00	1.00	1.00	1.00	0.00
	-	+	1.21	1.99	2.46	1.89	0.63
FaDu	+	-	1.00	1.00	1.00	1.00	0.00
	-	+	6.85	22.92	13.16	14.31	8.10
SCC25	+	-	1.00	1.000	1.00	1.00	0.00
	-	+	1.02	2.60	1.45	1.69	0.82
**Cell line**	**control plasmid**	**Cx43 plasmid**	**Densitometry intensity ratio of Bcl-2 after normalization to α-tubulin**				
			Sample1	Sample2	Sample3	Mean	SD
Detroit 562	+	-	1.00	1.00	1.00	1.00	0.00
	-	+	0.68	0.80	0.98	0.82	0.15
FaDu	+	-	1.00	1.00	1.00	1.00	0.00
	-	+	0.55	0.59	0.54	0.56	0.03
SCC25	+	-	1.00	1.00	1.00	1.00	0.00
	-	+	1.01	0.79	1.05	0.95	0.14

Table S17. Changes in the levels of Cx43 and Bcl-2 after Cx43 plasmid transfection in HNSCC cell lines. Densitometry analysis was performed using three independent experiments. The expression of all proteins were compared to expression of the the control plasmid treated controls, after normalization to α-tubulin. Red color indicate if $p < 0.05$. (Figure 4C).

	T-test for Independent Samples (plasmid)

Group 1 vs. Group 2	Note: Variables were treated as independent samples										
	Mean Group 1	Mean Group 2	t-value	df	p	N 1	N 2	Std.Dev. Group 1	Std.Dev. Group 2	F-ratio Variances	p Variances
Detroit 562 ctrl plasmid Cx43 vs Detroit 562 Cx43 plasmid Cx43	1	1.886667	-2.432396	4	0.071795	3	3	0	0.631374	0	1

Group 1 vs. Group 2	T-test for Independent Samples (plasmid) Note: Variables were treated as independent samples										
	Mean Group 1	Mean Group 2	t-value	df	p	N 1	N 2	Std.Dev. Group 1	Std.Dev. Group 2	F-ratio Variances	p Variances
Detroit 562 ctrl plasmid Cx43 Detroit 562 Cx43 plasmid Bcl2	1	0.82	2.064742	4	0.107871	3	3	0	0.150997	0	1

Group 1 vs. Group 2	T-test for Independent Samples (plasmid) Note: Variables were treated as independent samples										
	Mean Group 1	Mean Group 2	t-value	df	p	N 1	N 2	Std.Dev. Group 1	Std.Dev. Group 2	F-ratio Variances	p Variances
FaDu ctrl plasmid Cx43 vs. FaDu Cx43 plasmid Cx43	1	14.31	-2.847358	4	0.04652	3	3	0	8.09649	0	1

Group 1 vs. Group 2	T-test for Independent Samples (plasmid) Note: Variables were treated as independent samples										
	Mean Group 1	Mean Group 2	t-value	df	p	N 1	N 2	Std.Dev. Group 1	Std.Dev. Group 2	F-ratio Variances	p Variances
FaDu ctrl plasmid Cx43 vs. FaDu Cx43 plasmid Bcl2	1	0.56	28.80476	4	0.000009	3	3	0	0.02645751	0	1

Group 1 vs. Group 2	T-test for Independent Samples (plasmid) Note: Variables were treated as independent samples										
	Mean Group 1	Mean Group 2	t-value	df	p	N 1	N 2	Std.Dev. Group 1	Std.Dev. Group 2	F-ratio Variances	p Variances
SCC25 ctrl plasmid Cx43 vs. SCC25 Cx43 plasmid Cx43	1	1.69	-1.46302	4	0.217293	3	3	0	0.81688433	0	1

Group 1 vs. Group 2	T-test for Independent Samples (plasmid) Note: Variables were treated as independent samples										
	Mean Group 1	Mean Group 2	t-value	df	p	N 1	N 2	Std.Dev. Group 1	Std.Dev. Group 2	F-ratio Variances	p Variances
SCC25 ctrl plasmid Cx43 vs. SCC25 Cx43 plasmid Bcl2	1	0.95	0.61859	4	0.569673	3	3	0	0.14	0	1

Table S18. Changes in the effect of paclitaxel on cell viability after knocking down Cx43. HNSCC cell lines were analyzed by trypan blue exclusion test after 48 h of treatment with paclitaxel at different concentrations. Statistical analysis was performed by Student's t-test, the IC_{50} values of the cell lines were compared to each other. Red color indicate if $p < 0.05$. (Figure S3, Table S1).

Group 1 vs. Group 2	T-test for Independent Samples (Paclitaxel+siRNS_IC50) Note: Variables were treated as independent samples										
	Mean Group 1	Mean Group 2	t-value	df	p	N 1	N 2	Std.Dev. Group 1	Std.Dev. Group 2	F-ratio Variances	p Variances
Detroit 562 siControl vs. Detroit 562 siCx43	48.99333	42.76200	0.27665	4	0.795754	3	3	17.39978	34.91770	4.027206	0.397835
Detroit 562 siControl vs. FaDu siControl	48.99333	40.53333	0.44562	4	0.678924	3	3	17.39978	27.90144	2.571377	0.560008
Detroit 562 siControl vs. FaDu siCx43	48.99333	50.42333	-0.07347	4	0.944957	3	3	17.39978	28.87289	2.753551	0.532829
Detroit 562 siControl vs. SCC25 siControl	48.99333	11.97400	2.97495	4	0.040943	3	3	17.39978	12.71944	1.871333	0.696540
Detroit 562 siControl vs. SCC25 siCx43	48.99333	21.67967	1.98074	4	0.118702	3	3	17.39978	16.36191	1.130888	0.938576
Detroit 562 siCx43 vs. FaDu siControl	42.76200	40.53333	0.08636	4	0.935327	3	3	34.91770	27.90144	1.566167	0.779372
Detroit 562 siCx43 vs. FaDu siCx43	42.76200	50.42333	-0.29287	4	0.784183	3	3	34.91770	28.87289	1.462550	0.812166
Detroit 562 siCx43 vs. SCC25 siControl	42.76200	11.97400	1.43496	4	0.224621	3	3	34.91770	12.71944	7.536245	0.234295
Detroit 562 siCx43 vs. SCC25 siCx43	42.76200	21.67967	0.94696	4	0.397282	3	3	34.91770	16.36191	4.554318	0.360080
FaDu siControl vs. FaDu siCx43	40.53333	50.42333	-0.42663	4	0.691605	3	3	27.90144	28.87289	1.070847	0.965789
FaDu siControl vs. SCC25 siControl	40.53333	11.97400	1.61317	4	0.182003	3	3	27.90144	12.71944	4.811904	0.344121
FaDu siControl vs. SCC25 siCx43	40.53333	21.67967	1.00960	4	0.369800	3	3	27.90144	16.36191	2.907939	0.511779
FaDu siCx43 vs. SCC25 siControl	50.42333	11.97400	2.11079	4	0.102399	3	3	28.87289	12.71944	5.152812	0.325055
FaDu siCx43 vs. SCC25 siCx43	50.42333	21.67967	1.50016	4	0.207959	3	3	28.87289	16.36191	3.113957	0.486150
SCC25 siControl vs. SCC25 siCx43	11.97400	21.67967	-0.81116	4	0.462780	3	3	12.71944	16.36191	1.654747	0.753367

Table S19. Changes in the paclitaxel-induced apoptosis of SCC25 after knocking down Cx43. Annexin V-FLUOS/PI (Ann/PI)-stained HNSCC cells were analyzed by FACS after 48 h of treatment with paclitaxel at different concentrations. Live cells are presented by the Ann-/PI- fraction (LL), apoptotic cells by the Ann+/PI- fraction (LR), secondary necrotic cells by the Ann+/PI+ fraction (UR) and primary necrotic cells are detected in the Ann-/PI+ fraction (UL). (Figure 5).

Concentration (nM)		Paclitaxel-induced apoptosis of non-targeting siRNA treated SCC25 cell (%)				
		Sample 1	Sample 2	Sample 3	Mean	SD
Control	UL	0.36	0.93	1.61	1.0	0.625806
	UR	5.71	8.58	11.66	8.7	2.975618
	LL	86.94	83.85	82.28	84.4	2.370956
	LR	6.98	6.64	4.45	6.0	1.373111

1	UL	0.92	0.61		0.8	0.219203
	UR	5.01	7.24		6.1	1.576848
	LL	85.73	85.3		85.5	0.304056
	LR	8.34	6.85		7.6	1.053589
3	UL	1.04	0.6	1.62	1.1	0.511599
	UR	7.98	11.02	17.94	12.3	5.104403
	LL	79.28	76.97	76.24	77.5	1.586957
	LR	11.7	11.41	4.2	9.1	4.248886
10	UL	1.1	0.93	1.52	1.2	0.303699
	UR	11.78	16.07	11.74	13.2	2.48846
	LL	64.83	54.99	78.3	66.0	11.70201
	LR	22.29	28.01	8.44	19.6	10.06252
33	UL	0.69	0.67	1.96	1.1	0.739076
	UR	21.22	19.85	27.64	22.9	4.158874
	LL	22.03	27.21	30.69	26.6	4.357721
	LR	56.07	52.27	39.71	49.4	8.561962
100	UL	0.77	0.47	1.69	1.0	0.635715
	UR	22.12	19.68	24.54	22.1	2.430007
	LL	20.42	23.1	27.76	23.8	3.714243
	LR	56.69	56.75	46.01	53.2	6.183494

Concentration (nM)		Paclitaxel-induced apoptosis of *GJA1* siRNA treated SCC25 cell (%)				
		Sample 1	Sample 2	Sample 3	Mean	SD
Control	UL	1.02	0.64	1.54	1.07	0.451811
	UR	4.52	5.48	6.79	5.60	1.139488
	LL	89.22	90.3	88.71	89.41	0.81185
	LR	5.24	3.58	2.96	3.93	1.17887
1	UL	0.98	0.82	2.03	1.28	0.657292
	UR	4.6	9.28	6.31	6.73	2.368101
	LL	88.72	85.81	89.31	87.95	1.873775
	LR	5.7	4.09	2.35	4.05	1.67542
3	UL	0.97	1.06	1.79	1.27	0.449704
	UR	5.7	8.87	7.1	7.22	1.588595
	LL	85.82	84.15	88.16	86.04	2.014307
	LR	7.5	5.92	2.95	5.46	2.310115

Table S19. continuation

10	UL	1.23	1.18	1.52	1.31	0.183576
	UR	8.69	12.73	10.17	10.53	2.043918
	LL	77.44	74.62	83.85	78.64	4.72993
	LR	12.64	11.47	4.46	9.52	4.423826
33	UL	1.27	0.75	4.28	2.10	1.905754
	UR	21.63	22.2	16.57	20.13	3.09907
	LL	40.95	44.97	67.14	51.02	14.10429
	LR	36.15	32.08	12.01	26.75	12.92355
100	UL	1.01	0.57	2.8	1.46	1.181144
	UR	21.87	23.97	23.14	22.99	1.057655
	LL	38.25	41.38	49.19	42.94	5.634368
	LR	38.87	34.08	24.87	32.61	7.115338

Table S20. Changes in the paclitaxel-induced apoptosis of SCC25 after knocking down Cx43. Annexin V-FLUOS/PI (Ann/PI)-stained HNSCC cells were analyzed by FACS after 48 h of treatment with paclitaxel at different concentrations. Live cells are presented by the Ann-/PI- fraction (LL), apoptotic cells by the Ann+/PI- fraction (LR), secondary necrotic cells by the Ann+/PI+ fraction (UR) and primary necrotic cells are detected in the Ann-/PI+ fraction (UL). Statistical analysis was performed by Student's t-test, the cell fractions in all concentration were compared to negative control fractions in non-targeting siRNA or *GJA1* siRNA treated samples. The cell fractions in *GJA1* siRNA treated samples were also compared to cell fractions in non-targeting siRNA treated samples.
Red color indicate if $p < 0.05$. (Figure 5).

	T-test for Independent Samples (Paclitaxel_FACS_SCC25_non-targeting siRNA) Note: Variables were treated as independent samples										
Group 1 vs. Group 2	Mean Group 1	Mean Group 2	t-value	df	p	Valid N Group 1	Valid N Group 2	Std.Dev. Group 1	Std.Dev. Group 2	F-ratio Variances	p Variances
Control UL vs. 1 nM UL	0.966667	0.765000	0.41966	3	0.702972	3	2	0.625806	0.219203	8.15054	0.480832
Control UL vs. 3 nM UL	0.966667	1.086667	-0.25714	4	0.809759	3	3	0.625806	0.511599	1.49631	0.801184
Control UL vs. 10 nM UL	0.966667	1.183333	-0.53950	4	0.618173	3	3	0.625806	0.303699	4.24611	0.381234
Control UL vs. 33 nM UL	0.966667	1.106667	-0.25039	4	0.814620	3	3	0.625806	0.739076	1.39476	0.835158
Control UL vs. 100 nM UL	0.966667	0.976667	-0.01942	4	0.985439	3	3	0.625806	0.635715	1.03192	0.984292
1 nM UL vs. 3 nM UL	0.765000	1.086667	-0.80731	3	0.478558	2	3	0.219203	0.511599	5.44710	0.579912
1 nM UL vs. 10 nM UL	0.765000	1.183333	-1.64606	3	0.198303	2	3	0.219203	0.303699	1.91953	0.909180
1 nM UL vs. 33 nM UL	0.765000	1.106667	-0.60702	3	0.586698	2	3	0.219203	0.739076	11.36802	0.410512
1 nM UL vs. 100 nM UL	0.765000	0.976667	-0.43400	3	0.693608	2	3	0.219203	0.635715	8.41068	0.473761
3 nM UL vs. 10 nM UL	1.086667	1.183333	-0.28142	4	0.792346	3	3	0.511599	0.303699	2.83773	0.521141

3 nM UL vs. 33 nM UL	1.086667	1.106667	-0.03854	4	0.971105	3	3	0.511599	0.739076	2.08698	0.647882
3 nM UL vs. 100 nM UL	1.086667	0.976667	0.23349	4	0.826847	3	3	0.511599	0.635715	1.54407	0.786143
10 nM UL vs. 33 nM UL	1.183333	1.106667	0.16619	4	0.876071	3	3	0.303699	0.739076	5.92230	0.288921
10 nM UL vs. 100 nM UL	1.183333	0.976667	0.50808	4	0.638136	3	3	0.303699	0.635715	4.38164	0.371634
33 nM UL vs. 100 nM UL	1.106667	0.976667	0.23097	4	0.828670	3	3	0.739076	0.635715	1.35162	0.850479

Table S20. continuation

Group 1 vs. Group 2	T-test for Independent Samples (Paclitaxel_FACS_SCC25_non-targeting siRNA) Note: Variables were treated as independent samples										
	Mean Group 1	Mean Group 2	t-value	df	p	Valid N Group 1	Valid N Group 2	Std.Dev. Group 1	Std.Dev. Group 2	F-ratio Variances	p Variances
Control UR vs. 1 nM UR	8.65000	6.12500	1.06608	3	0.364570	3	2	2.975618	1.576848	3.56102	0.701774
Control UR vs. 3 nM UR	8.65000	12.31333	-1.07391	4	0.343331	3	3	2.975618	5.104403	2.94263	0.507276
Control UR vs. 10 nM UR	8.65000	13.19667	-2.03017	4	0.112191	3	3	2.975618	2.488460	1.42986	0.823093
Control UR vs. 33 nM UR	8.65000	22.90333	-4.82766	4	0.008476	3	3	2.975618	4.158874	1.95343	0.677179
Control UR vs. 100 nM UR	8.65000	22.11333	-6.06990	4	0.003721	3	3	2.975618	2.430007	1.49948	0.800168
1 nM UR vs. 3 nM UR	6.12500	12.31333	-1.58907	3	0.210259	2	3	1.576848	5.104403	10.47877	0.426814
1 nM UR vs. 10 nM UR	6.12500	13.19667	-3.47935	3	0.040074	2	3	1.576848	2.488460	2.49047	0.817796
1 nM UR vs. 33 nM UR	6.12500	22.90333	-5.22801	3	0.013615	2	3	1.576848	4.158874	6.95620	0.517912
1 nM UR vs. 100 nM UR	6.12500	22.11333	-8.02310	3	0.004043	2	3	1.576848	2.430007	2.37484	0.834080
3 nM UR vs. 10 nM UR	12.31333	13.19667	-0.26943	4	0.800930	3	3	5.104403	2.488460	4.20754	0.384058
3 nM UR vs. 33 nM UR	12.31333	22.90333	-2.78584	4	0.049522	3	3	5.104403	4.158874	1.50639	0.797959
3 nM UR vs. 100 nM UR	12.31333	22.11333	-3.00251	4	0.039843	3	3	5.104403	2.430007	4.41240	0.369522
10 nM UR vs. 33 nM UR	13.19667	22.90333	-3.46898	4	0.025607	3	3	2.488460	4.158874	2.79312	0.527270
10 nM UR vs. 100 nM UR	13.19667	22.11333	-4.44036	4	0.011331	3	3	2.488460	2.430007	1.04869	0.976234
33 nM UR vs. 100 nM UR	22.90333	22.11333	0.28407	4	0.790452	3	3	4.158874	2.430007	2.92912	0.509020

Table S20. *continuation*

Group 1 vs. Group 2	T-test for Independent Samples (Paclitaxel_FACS_SCC25_non-targeting siRNA) Note: Variables were treated as independent samples										
	Mean Group 1	Mean Group 2	t-value	df	p	Valid N Group 1	Valid N Group 2	Std.Dev. Group 1	Std.Dev. Group 2	F-ratio Variances	p Variances
Control LL vs. 1 nM LL	84.35667	85.51500	-0.6528	3	0.560459	3	2	2.37096	0.30406	60.805	0.180620

Control LL vs. 3 nM LL	84.35667	77.49667	4.1646	4	0.014092	3	3	2.37096	1.58696	2.232	0.618790
Control LL vs. 10 nM LL	84.35667	66.04000	2.6571	4	0.056560	3	3	2.37096	11.70201	24.360	0.078865
Control LL vs. 33 nM LL	84.35667	26.64333	20.1498	4	0.000036	3	3	2.37096	4.35772	3.378	0.456820
Control LL vs. 100 nM LL	84.35667	23.76000	23.8187	4	0.000018	3	3	2.37096	3.71424	2.454	0.579021
1 nM LL vs. 3 nM LL	85.51500	77.49667	6.7175	3	0.006734	2	3	0.30406	1.58696	27.241	0.268506
1 nM LL vs. 10 nM LL	85.51500	66.04000	2.2324	3	0.111743	2	3	0.30406	11.70201	1481.202	0.036740
1 nM LL vs. 33 nM LL	85.51500	26.64333	18.1032	3	0.000368	2	3	0.30406	4.35772	205.405	0.098556
1 nM LL vs. 100 nM LL	85.51500	23.76000	22.2696	3	0.000198	2	3	0.30406	3.71424	149.222	0.115577
3 nM LL vs. 10 nM LL	77.49667	66.04000	1.6804	4	0.168185	3	3	1.58696	11.70201	54.374	0.036118
3 nM LL vs. 33 nM LL	77.49667	26.64333	18.9923	4	0.000045	3	3	1.58696	4.35772	7.540	0.234184
3 nM LL vs. 100 nM LL	77.49667	23.76000	23.0436	4	0.000021	3	3	1.58696	3.71424	5.478	0.308744
10 nM LL vs. 33 nM LL	66.04000	26.64333	5.4646	4	0.005454	3	3	11.70201	4.35772	7.211	0.243572
10 nM LL vs. 100 nM LL	66.04000	23.76000	5.9647	4	0.003967	3	3	11.70201	3.71424	9.926	0.183047
33 nM LL vs. 100 nM LL	26.64333	23.76000	0.8722	4	0.432326	3	3	4.35772	3.71424	1.377	0.841571

***Table S20.** continuation*

Group 1 vs. Group 2	T-test for Independent Samples (Paclitaxel_FACS_SCC25_non-targeting siRNA) Note: Variables were treated as independent samples										
	Mean Group 1	Mean Group 2	t-value	df	p	Valid N Group 1	Valid N Group 2	Std.Dev. Group 1	Std.Dev. Group 2	F-ratio Variances	p Variances
Control LR vs. 1 nM LR	6.02333	7.59500	-1.3498	3	0.269904	3	2	1.37311	1.05359	1.69851	0.953785
Control LR vs. 3 nM LR	6.02333	9.10333	-1.1947	4	0.298193	3	3	1.37311	4.24889	9.57500	0.189125
Control LR vs. 10 nM LR	6.02333	19.58000	-2.3121	4	0.081846	3	3	1.37311	10.06252	53.70346	0.036561
Control LR vs. 33 nM LR	6.02333	49.35000	-8.6542	4	0.000981	3	3	1.37311	8.56196	38.88082	0.050149
Control LR vs. 100 nM LR	6.02333	53.15000	-12.8867	4	0.000209	3	3	1.37311	6.18349	20.27948	0.093987
1 nM LR vs. 3 nM LR	7.59500	9.10333	-0.4691	3	0.670972	2	3	1.05359	4.24889	16.26326	0.345411
1 nM LR vs. 10 nM LR	7.59500	19.58000	-1.5936	3	0.209279	2	3	1.05359	10.06252	91.21598	0.147670
1 nM LR vs. 33 nM LR	7.59500	49.35000	-6.5183	3	0.007336	2	3	1.05359	8.56196	66.03955	0.173370
1 nM LR vs. 100 nM LR	7.59500	53.15000	-9.8132	3	0.002249	2	3	1.05359	6.18349	34.44493	0.239234
3 nM LR vs. 10 nM LR	9.10333	19.58000	-1.6613	4	0.171988	3	3	4.24889	10.06252	5.60871	0.302631

3 nM LR vs. 33 nM LR	9.10333	49.35000	-7.2931	4	0.001879	3	3	4.24889	8.56196	4.06066	0.395205
3 nM LR vs. 100 nM LR	9.10333	53.15000	-10.1687	4	0.000527	3	3	4.24889	6.18349	2.11796	0.641445
10 nM LR vs. 33 nM LR	19.58000	49.35000	-3.9027	4	0.017501	3	3	10.06252	8.56196	1.38123	0.839901
10 nM LR vs. 100 nM LR	19.58000	53.15000	-4.9231	4	0.007912	3	3	10.06252	6.18349	2.64817	0.548220
33 nM LR vs. 100 nM LR	49.35000	53.15000	-0.6232	4	0.566930	3	3	8.56196	6.18349	1.91725	0.685577

Table S20. continuation

Group 1 vs. Group 2	T-test for Independent Samples (Paclitaxel_FACS_SCC25_GJA1 siRNA) Note: Variables were treated as independent samples										
	Mean Group 1	Mean Group 2	t-value	df	p	Valid N Group 1	Valid N Group 2	Std.Dev. Group 1	Std.Dev. Group 2	F-ratio Variances	p Variances
Control UL vs. 1 nM UL	1.066667	1.276667	-0.456031	4	0.672029	3	3	0.451811	0.657292	2.1164	0.641761
Control UL vs. 3 nM UL	1.066667	1.273333	-0.561529	4	0.604407	3	3	0.451811	0.449704	1.0094	0.995324
Control UL vs. 10 nM UL	1.066667	1.310000	-0.864223	4	0.436211	3	3	0.451811	0.183576	6.0574	0.283392
Control UL vs. 33 nM UL	1.066667	2.100000	-0.913818	4	0.412512	3	3	0.451811	1.905754	17.7918	0.106429
Control UL vs. 100 nM UL	1.066667	1.460000	-0.538723	4	0.618661	3	3	0.451811	1.181144	6.8343	0.255289
1 nM UL vs. 3 nM UL	1.276667	1.273333	0.007249	4	0.994563	3	3	0.657292	0.449704	2.1363	0.637692
1 nM UL vs. 10 nM UL	1.276667	1.310000	-0.084600	4	0.936644	3	3	0.657292	0.183576	12.8200	0.144718
1 nM UL vs. 33 nM UL	1.276667	2.100000	-0.707397	4	0.518357	3	3	0.657292	1.905754	8.4065	0.212618
1 nM UL vs. 100 nM UL	1.276667	1.460000	-0.234918	4	0.825808	3	3	0.657292	1.181144	3.2291	0.472908
3 nM UL vs. 10 nM UL	1.273333	1.310000	-0.130749	4	0.902286	3	3	0.449704	0.183576	6.0010	0.285674
3 nM UL vs. 33 nM UL	1.273333	2.100000	-0.731236	4	0.505166	3	3	0.449704	1.905754	17.9590	0.105491
3 nM UL vs. 100 nM UL	1.273333	1.460000	-0.255817	4	0.810709	3	3	0.449704	1.181144	6.8985	0.253214
10 nM UL vs. 33 nM UL	1.310000	2.100000	-0.714686	4	0.514297	3	3	0.183576	1.905754	107.7715	0.018387
10 nM UL vs. 100 nM UL	1.310000	1.460000	-0.217353	4	0.838570	3	3	0.183576	1.181144	41.3976	0.047172
33 nM UL vs. 100 nM UL	2.100000	1.460000	0.494409	4	0.646940	3	3	1.905754	1.181144	2.6033	0.555043

Table S20. continuation

Group 1 vs. Group 2	T-test for Independent Samples (Paclitaxel_FACS_SCC25_GJA1 siRNA) Note: Variables were treated as independent samples										
	Mean Group 1	Mean Group 2	t-value	df	p	Valid N Group 1	Valid N Group 2	Std.Dev. Group 1	Std.Dev. Group 2	F-ratio Variances	p Variances
Control UR vs. 1 nM UR	5.59667	6.73000	-0.7470	4	0.496605	3	3	1.139488	2.368101	4.318974	0.376012

Control UR vs. 3 nM UR	5.59667	7.22333	-1.4412	4	0.222983	3	3	1.139488	1.588595	1.943599	0.679440
Control UR vs. 10 nM UR	5.59667	10.53000	-3.6515	4	0.021743	3	3	1.139488	2.043918	3.217416	0.474224
Control UR vs. 33 nM UR	5.59667	20.13333	-7.6253	4	0.001588	3	3	1.139488	3.099070	7.396786	0.238186
Control UR vs. 100 nM UR	5.59667	22.99333	-19.3813	4	0.000042	3	3	1.139488	1.057655	1.160732	0.925612
1 nM UR vs. 3 nM UR	6.73000	7.22333	-0.2997	4	0.779369	3	3	2.368101	1.588595	2.222153	0.620703
1 nM UR vs. 10 nM UR	6.73000	10.53000	-2.1040	4	0.103182	3	3	2.368101	2.043918	1.342374	0.853835
1 nM UR vs. 33 nM UR	6.73000	20.13333	-5.9522	4	0.003998	3	3	2.368101	3.099070	1.712626	0.737293
1 nM UR vs. 100 nM UR	6.73000	22.99333	-10.8611	4	0.000408	3	3	2.368101	1.057655	5.013171	0.332603
3 nM UR vs. 10 nM UR	7.22333	10.53000	-2.2125	4	0.091376	3	3	1.588595	2.043918	1.655391	0.753185
3 nM UR vs. 33 nM UR	7.22333	20.13333	-6.4209	4	0.003024	3	3	1.588595	3.099070	3.805717	0.416171
3 nM UR vs. 100 nM UR	7.22333	22.99333	-14.3122	4	0.000138	3	3	1.588595	1.057655	2.255997	0.614251
10 nM UR vs. 33 nM UR	10.53000	20.13333	-4.4805	4	0.010985	3	3	2.043918	3.099070	2.298983	0.606247
10 nM UR vs. 100 nM UR	10.53000	22.99333	-9.3802	4	0.000720	3	3	2.043918	1.057655	3.734557	0.422426
33 nM UR vs. 100 nM UR	20.13333	22.99333	-1.5128	4	0.204887	3	3	3.099070	1.057655	8.585685	0.208644

Table S20. *continuation*

Group 1 vs. Group 2	T-test for Independent Samples (Paclitaxel_FACS_SCC25_GJA1 siRNA) Note: Variables were treated as independent samples										
	Mean Group 1	Mean Group 2	t-value	df	p	Valid N Group 1	Valid N Group 2	Std.Dev. Group 1	Std.Dev. Group 2	F-ratio Variances	p Variances
Control LL vs. 1 nM LL	89.41000	87.94667	1.2412	4	0.282360	3	3	0.81185	1.87378	5.3270	0.316105
Control LL vs. 3 nM LL	89.41000	86.04333	2.6850	4	0.054941	3	3	0.81185	2.01431	6.1560	0.279485
Control LL vs. 10 nM LL	89.41000	78.63667	3.8882	4	0.017717	3	3	0.81185	4.72993	33.9436	0.057235
Control LL vs. 33 nM LL	89.41000	51.02000	4.7066	4	0.009263	3	3	0.81185	14.10429	301.8220	0.006605
Control LL vs. 100 nM LL	89.41000	42.94000	14.1392	4	0.000145	3	3	0.81185	5.63437	48.1658	0.040679
1 nM LL vs. 3 nM LL	87.94667	86.04333	1.1983	4	0.296937	3	3	1.87378	2.01431	1.1556	0.927806
1 nM LL vs. 10 nM LL	87.94667	78.63667	3.1696	4	0.033872	3	3	1.87378	4.72993	6.3720	0.271298
1 nM LL vs. 33 nM LL	87.94667	51.02000	4.4952	4	0.010862	3	3	1.87378	14.10429	56.6588	0.034687
1 nM LL vs. 100 nM LL	87.94667	42.94000	13.1285	4	0.000194	3	3	1.87378	5.63437	9.0418	0.199167
3 nM LL vs. 10 nM LL	86.04333	78.63667	2.4954	4	0.067097	3	3	2.01431	4.72993	5.5139	0.307036

3 nM LL vs. 33 nM LL	86.04333	51.02000	4.2578	4	0.013077	3	3	2.01431	14.10429	49.0288	0.039977
3 nM LL vs. 100 nM LL	86.04333	42.94000	12.4770	4	0.000237	3	3	2.01431	5.63437	7.8242	0.226650
10 nM LL vs. 33 nM LL	78.63667	51.02000	3.2154	4	0.032421	3	3	4.72993	14.10429	8.8919	0.202186
10 nM LL vs. 100 nM LL	78.63667	42.94000	8.4046	4	0.001097	3	3	4.72993	5.63437	1.4190	0.826789
33 nM LL vs. 100 nM LL	51.02000	42.94000	0.9214	4	0.408964	3	3	14.10429	5.63437	6.2663	0.275243

Table S20. continuation

Group 1 vs. Group 2	T-test for Independent Samples (Paclitaxel_FACS_SCC25_GJA1 siRNA) Note: Variables were treated as independent samples										
	Mean Group 1	Mean Group 2	t-value	df	p	Valid N Group 1	Valid N Group 2	Std.Dev. Group 1	Std.Dev. Group 2	F-ratio Variances	p Variances
Control LR vs. 1 nM LR	3.92667	4.04667	-0.10146	4	0.924070	3	3	1.17887	1.67542	2.0198	0.662288
Control LR vs. 3 nM LR	3.92667	5.45667	-1.02179	4	0.364647	3	3	1.17887	2.31012	3.8400	0.413220
Control LR vs. 10 nM LR	3.92667	9.52333	-2.11736	4	0.101644	3	3	1.17887	4.42383	14.0820	0.132608
Control LR vs. 33 nM LR	3.92667	26.74667	-3.04575	4	0.038186	3	3	1.17887	12.92355	120.1801	0.016504
Control LR vs. 100 nM LR	3.92667	32.60667	-6.88754	4	0.002329	3	3	1.17887	7.11534	36.4300	0.053433
1 nM LR vs. 3 nM LR	4.04667	5.45667	-0.85580	4	0.440347	3	3	1.67542	2.31012	1.9012	0.689378
1 nM LR vs. 10 nM LR	4.04667	9.52333	-2.00527	4	0.115420	3	3	1.67542	4.42383	6.9719	0.250883
1 nM LR vs. 33 nM LR	4.04667	26.74667	-3.01707	4	0.039276	3	3	1.67542	12.92355	59.4999	0.033058
1 nM LR vs. 100 nM LR	4.04667	32.60667	-6.76715	4	0.002488	3	3	1.67542	7.11534	18.0361	0.105063
3 nM LR vs. 10 nM LR	5.45667	9.52333	-1.41137	4	0.230976	3	3	2.31012	4.42383	3.6671	0.428527
3 nM LR vs. 33 nM LR	5.45667	26.74667	-2.80882	4	0.048375	3	3	2.31012	12.92355	31.2966	0.061926
3 nM LR vs. 100 nM LR	5.45667	32.60667	-6.28599	4	0.003271	3	3	2.31012	7.11534	9.4869	0.190714
10 nM LR vs. 33 nM LR	9.52333	26.74667	-2.18391	4	0.094331	3	3	4.42383	12.92355	8.5343	0.209769
10 nM LR vs. 100 nM LR	9.52333	32.60667	-4.77195	4	0.008827	3	3	4.42383	7.11534	2.5870	0.557570
33 nM LR vs. 100 nM LR	26.74667	32.60667	-0.68799	4	0.529279	3	3	12.92355	7.11534	3.2989	0.465232

Table S20. continuation

Group 1 vs. Group 2	T-test for Independent Samples (Paclitaxel_FACS_SCC25_non-targeting siRNA + GJA1 siRNA) Note: Variables were treated as independent samples										
	Mean Group 1	Mean Group 2	t-value	df	p	N 1	N 2	Std.Dev. Group 1	Std.Dev. Group 2	F-ratio Variances	p Variances
siControl Control UL vs. siGJA1 Control UL	0.96667	1.06667	-0.224	4	0.833443	3	3	0.62581	0.45181	1.919	0.685279
siControl Control UR vs. siGJA1 Control UR	8.650000	5.596667	1.659753	4	0.172302	3	3	2.975618	1.139488	6.819218	0.255780
siControl Control LL vs. siGJA1 Control LL	84.35667	89.41000	-3.49253	4	0.025065	3	3	2.370956	0.811850	8.528954	0.209887
siControl Control LR vs. siGJA1 Control LR	6.023333	3.926667	2.006658	4	0.115237	3	3	1.373111	1.178870	1.356687	0.848649
siControl 1 nM UL vs. siGJA1 1 nM UL	0.765000	1.276667	-1.01651	3	0.384230	2	3	0.219203	0.657292	8.991328	0.459041
siControl 1 nM UR vs. siGJA1 1 nM UR	6.125000	6.730000	-0.310106	3	0.776774	2	3	1.576848	2.368101	2.255384	0.851969
siControl 1 nM LL vs. siGJA1 1 nM LL	85.51500	87.94667	-1.72975	3	0.182114	2	3	0.304056	1.873775	37.97765	0.227987
siControl 1 nM LR vs. siGJA1 1 nM LR	7.595	4.04666667	2.59631894	3	0.0806319866	2	3	1.0535891	1.67542035	2.52874495	0.812612776
siControl 3 nM UL vs. siGJA1 3 nM UL	1.086667	1.273333	-0.474662	4	0.659782	3	3	0.511599	0.449704	1.294215	0.871758
siControl 3 nM UR vs. siGJA1 3 nM UR	12.31333	7.223333	1.649143	4	0.174464	3	3	5.104403	1.588595	10.32437	0.176610
siControl 3 nM LL vs. siGJA1 3 nM LL	77.49667	86.04333	-5.77273	4	0.004471	3	3	1.586957	2.014307	1.611094	0.765962
siControl 3 nM LR vs. siGJA1 3 nM LR	9.103333	5.456667	1.306005	4	0.261592	3	3	4.248886	2.310115	3.382851	0.456324
siControl 10 nM UL vs. siGJA1 10 nM UL	1.183333	1.310000	-0.618234	4	0.569886	3	3	0.303699	0.183576	2.736894	0.535204
siControl 10 nM UR vs. siGJA1 10 nM UR	13.19667	10.53000	1.434298	4	0.224798	3	3	2.488460	2.043918	1.482294	0.805706
siControl 10 nM LL vs. siGJA1 10 nM LL	66.04000	78.63667	-1.72860	4	0.158942	3	3	11.70201	4.729930	6.120851	0.280865
siControl 10 nM LR vs. siGJA1 10 nM LR	19.58000	9.523333	1.584664	4	0.188220	3	3	10.06252	4.423826	5.173893	0.323945
siControl 33 nM UL vs. siGJA1 33 nM UL	1.106667	2.100000	-0.841714	4	0.447328	3	3	0.739076	1.905754	6.648990	0.261472
siControl 33 nM UR vs. siGJA1 33 nM UR	22.90333	20.13333	0.925040	4	0.407300	3	3	4.158874	3.099070	1.800897	0.714057
siControl 33 nM LL vs. siGJA1 33 nM LL	26.64333	51.02000	-2.86013	4	0.045923	3	3	4.357721	14.10429	10.47571	0.174281
siControl 33 nM LR vs. siGJA1 33 nM LR	49.35000	26.74667	2.525418	4	0.064981	3	3	8.561962	12.92355	2.278333	0.610066

Table S20. continuation

siControl 100 nM UL vs. siGJA1 100 nM UL	0.976666667	1.46	-0.624113577	4	0.566382804	3	3	0.635714821	1.18114351	3.45207852	0.449228375

siControl 100 nM UR vs. siGJA1 100 nM UR	22.1133333	22.9933333	-0.575127912	4	0.596007662	3	3	2.43000686	1.05765464	5.27870318	0.318537116
siControl 100 nM LL vs. siGJA1 100 nM LL	23.76000	42.94000	-4.92271	4	0.007914	3	3	3.714243	5.634368	2.301176	0.605845
siControl 100 nM LR vs. siGJA1 100 nM LR	53.15000	32.60667	3.774588	4	0.019527	3	3	6.183494	7.115338	1.324107	0.860546

Table S21. Changes in the paclitaxel-induced apoptosis of Detroit 562 after knocking down Cx43. Annexin V-FLUOS/PI (Ann/PI)-stained HNSCC cells were analyzed by FACS after 48 h of treatment with paclitaxel at different concentrations. Live cells are presented by the Ann-/PI- fraction (LL), apoptotic cells by the Ann+/PI- fraction (LR), secondary necrotic cells by the Ann+/PI+ fraction (UR) and primary necrotic cells are detected in the Ann-/PI+ fraction (UL). (Figure S1).

Concentration (nM)		Paclitaxel-induced apoptosis of non-targeting siRNA treated Detroit 562 cell (%)				
		Sample 1	Sample 2	Sample 3	Mean	SD
Control	UL	2.27	5.11	4.42	3.9	1.481227
	UR	9.65	23.53	21.54	18.2	7.505405
	LL	85.95	61.36	64.44	70.6	13.39673
	LR	2.13	9.99	9.6	7.2	4.429684
1	UL	1.92	3.92	5.14	3.7	1.625669
	UR	10.31	19.98	21.01	17.1	5.902821
	LL	85.6	65.96	64.5	72.0	11.78326
	LR	2.17	10.14	9.35	7.2	4.39123
3	UL	1.48	4.73	4.74	3.7	1.879282
	UR	8.37	23.57	20.16	17.4	7.975715
	LL	87.51	62.06	65.35	71.6	13.84192
	LR	2.64	9.64	9.75	7.3	4.073577
10	UL	1.89	5.4	7.21	4.8	2.704891
	UR	10.45	25.26	25.36	20.4	8.579571
	LL	85.42	57.81	57.43	66.9	16.05146
	LR	2.25	11.52	10	7.9	4.971683
33	UL	5.71	10.01	9.09	8.3	2.264244
	UR	16.41	42.56	41.89	33.6	14.90806
	LL	71.68	30.71	33.3	45.2	22.94295
	LR	6.2	16.71	15.72	12.9	5.803312
100	UL	6.43	10.79	8.61	8.6	2.18

	UR	16.22	43.17	44.93	34.8	16.09174
	LL	68.53	29.88	29.38	42.6	22.46032
	LR	8.82	16.15	17.08	14.0	4.524404

Concentration (nM)		Paclitaxel-induced apoptosis of *GJA1* siRNA treated Detroit 562 cell (%)				
		Sample 1	Sample 2	Sample 3	Mean	SD
Control	UL	1.14	2.32	2.36	1.94	0.693109
	UR	7.67	13.45	13.19	11.44	3.264618
	LL	89.03	76.51	77.14	80.89	7.053597
	LR	2.16	7.72	7.31	5.73	3.0985
1	UL	0.65	1.90	2.41	1.65	0.905557
	UR	4.97	11.74	17.70	11.47	6.369294
	LL	91.26	80.33	72.84	81.48	9.263381
	LR	3.12	6.03	7.05	5.40	2.039338
3	UL	0.75	1.67	2.61	1.68	0.930018
	UR	9.40	11.65	15.48	12.18	3.074026
	LL	87.29	79.93	74.43	80.55	6.452379
	LR	2.56	6.75	7.48	5.60	2.655039

Table S21. continuation

10	UL	1.92	3.72	4.33	3.32	1.25301
	UR	6.67	12.50	16.13	11.77	4.772445
	LL	88.79	75.33	69.40	77.84	9.935698
	LR	2.61	8.45	10.14	7.07	3.951004
33	UL	3.67	9.91	6.73	6.77	3.120192
	UR	13.15	27.32	33.32	24.60	10.35711
	LL	74.76	46.26	44.53	55.18	16.97594
	LR	8.42	16.51	15.42	13.45	4.390068
100	UL	4.78	11.86	7.40	8.01	3.579628
	UR	11.96	32.33	37.78	27.36	13.60951
	LL	71.41	38.77	39.04	49.74	18.76726
	LR	11.85	17.04	15.78	14.89	2.707046

Table S22. Changes in the paclitaxel-induced apoptosis of Detroit 562 after knocking down Cx43. Annexin V-FLUOS/PI (Ann/PI)-stained HNSCC cells were analyzed by FACS after 48 h of treatment with paclitaxel at different concentrations. Live cells are presented by the Ann-/PI- fraction (LL), apoptotic cells by the Ann+/PI- fraction (LR), secondary necrotic cells by the Ann+/PI+ fraction (UR) and primary necrotic cells are detected in the Ann-/PI+ fraction (UL). Statistical analysis was performed by Student’s t-test, the cell fractions in all concentration were compared to negative control fractions in non-targeting siRNA or *GJA1* siRNA treated samples. The cell fractions in *GJA1* siRNA treated samples were also compared to cell

fractions in non-targeting siRNA treated samples.
Red color indicate if p < 0.05. (Figure S1).

Group 1 vs. Group 2	T-test for Independent Samples (Paclitaxel_FACS_Detroit562_non-targeting siRNA) Note: Variables were treated as independent samples										
	Mean Group 1	Mean Group 2	t-value	df	p	Valid N Group 1	Valid N Group 2	Std.Dev. Group 1	Std.Dev. Group 2	F-ratio Variances	p Variances
Control UL vs. 1 nM UL	3.933333	3.660000	0.21526	4	0.840091	3	3	1.481227	1.625669	1.204540	0.907219
Control UL vs. 3 nM UL	3.933333	3.650000	0.20509	4	0.847516	3	3	1.481227	1.879282	1.609684	0.766376
Control UL vs. 10 nM UL	3.933333	4.833333	-0.50548	4	0.639805	3	3	1.481227	2.704891	3.334696	0.461393
Control UL vs. 33 nM UL	3.933333	8.270000	-2.77611	4	0.050017	3	3	1.481227	2.264244	2.336701	0.599394
Control UL vs. 100 nM UL	3.933333	8.610000	-3.07338	4	0.037170	3	3	1.481227	2.180000	2.166056	0.631701
1 nM UL vs. 3 nM UL	3.660000	3.650000	0.00697	4	0.994772	3	3	1.625669	1.879282	1.336348	0.856037
1 nM UL vs. 10 nM UL	3.660000	4.833333	-0.64398	4	0.554658	3	3	1.625669	2.704891	2.768440	0.530724
1 nM UL vs. 33 nM UL	3.660000	8.270000	-2.86459	4	0.045717	3	3	1.625669	2.264244	1.939912	0.680292
1 nM UL vs. 100 nM UL	3.660000	8.610000	-3.15276	4	0.034423	3	3	1.625669	2.180000	1.798244	0.714734
3 nM UL vs. 10 nM UL	3.650000	4.833333	-0.62229	4	0.567470	3	3	1.879282	2.704891	2.071646	0.651117
3 nM UL vs. 33 nM UL	3.650000	8.270000	-2.71945	4	0.053017	3	3	1.879282	2.264244	1.451652	0.815776
3 nM UL vs. 100 nM UL	3.650000	8.610000	-2.98483	4	0.040545	3	3	1.879282	2.180000	1.345641	0.852645
10 nM UL vs. 33 nM UL	4.833333	8.270000	-1.68745	4	0.166791	3	3	2.704891	2.264244	1.427096	0.824030
10 nM UL vs. 100 nM UL	4.833333	8.610000	-1.88294	4	0.132829	3	3	2.704891	2.180000	1.539524	0.787549
33 nM UL vs. 100 nM UL	8.270000	8.610000	-0.18736	4	0.860498	3	3	2.264244	2.180000	1.078781	0.962102

Table S22. continuation

Group 1 vs. Group 2	T-test for Independent Samples (Paclitaxel_FACS_Detroit562_non-targeting siRNA) Note: Variables were treated as independent samples										
	Mean Group 1	Mean Group 2	t-value	df	p	Valid N Group 1	Valid N Group 2	Std.Dev. Group 1	Std.Dev. Group 2	F-ratio Variances	p Variances
Control UR vs. 1 nM UR	18.24000	17.10000	0.20679	4	0.846274	3	3	7.50540	5.90282	1.616698	0.764322
Control UR vs. 3 nM UR	18.24000	17.36667	0.13812	4	0.896821	3	3	7.50540	7.97572	1.129252	0.939297
Control UR vs. 10 nM UR	18.24000	20.35667	-0.32162	4	0.763847	3	3	7.50540	8.57957	1.306721	0.867032
Control UR vs. 33 nM UR	18.24000	33.62000	-1.59603	4	0.185716	3	3	7.50540	14.90806	3.945428	0.404414
Control UR vs. 100 nM UR	18.24000	34.77333	-1.61278	4	0.182087	3	3	7.50540	16.09174	4.596822	0.357346
1 nM UR vs. 3 nM UR	17.10000	17.36667	-0.04655	4	0.965104	3	3	5.90282	7.97572	1.825660	0.707799

1 nM UR vs. 10 nM UR	17.10000	20.35667	-0.54164	4	0.616823	3	3	5.90282	8.57957	2.112574	0.642555
1 nM UR vs. 33 nM UR	17.10000	33.62000	-1.78453	4	0.148895	3	3	5.90282	14.90806	6.378566	0.271055
1 nM UR vs. 100 nM UR	17.10000	34.77333	-1.78592	4	0.148655	3	3	5.90282	16.09174	7.431674	0.237201
3 nM UR vs. 10 nM UR	17.36667	20.35667	-0.44210	4	0.681268	3	3	7.97572	8.57957	1.157156	0.927147
3 nM UR vs. 33 nM UR	17.36667	33.62000	-1.66504	4	0.171236	3	3	7.97572	14.90806	3.493841	0.445054
3 nM UR vs. 100 nM UR	17.36667	34.77333	-1.67870	4	0.168511	3	3	7.97572	16.09174	4.070677	0.394425
10 nM UR vs. 33 nM UR	20.35667	33.62000	-1.33558	4	0.252616	3	3	8.57957	14.90806	3.019335	0.497595
10 nM UR vs. 100 nM UR	20.35667	34.77333	-1.36929	4	0.242755	3	3	8.57957	16.09174	3.517830	0.442690
33 nM UR vs. 100 nM UR	33.62000	34.77333	-0.09107	4	0.931818	3	3	14.90806	16.09174	1.165101	0.923744

Table S22. *continuation*

Group 1 vs. Group 2	T-test for Independent Samples (Paclitaxel_FACS_Detroit562_non-targeting siRNA) Note: Variables were treated as independent samples										
	Mean Group 1	Mean Group 2	t-value	df	p	Valid N Group 1	Valid N Group 2	Std.Dev. Group 1	Std.Dev. Group 2	F-ratio Variances	p Variances
Control LL vs. 1 nM LL	70.58333	72.02000	-0.13947	4	0.895818	3	3	13.39673	11.78326	1.292608	0.872369
Control LL vs. 3 nM LL	70.58333	71.64000	-0.09501	4	0.928876	3	3	13.39673	13.84192	1.067566	0.967321
Control LL vs. 10 nM LL	70.58333	66.88667	0.30625	4	0.774696	3	3	13.39673	16.05146	1.435593	0.821155
Control LL vs. 33 nM LL	70.58333	45.23000	1.65287	4	0.173701	3	3	13.39673	22.94295	2.932923	0.508528
Control LL vs. 100 nM LL	70.58333	42.59667	1.85355	4	0.137424	3	3	13.39673	22.46032	2.810826	0.524821
1 nM LL vs. 3 nM LL	72.02000	71.64000	0.03621	4	0.972852	3	3	11.78326	13.84192	1.379945	0.840356
1 nM LL vs. 10 nM LL	72.02000	66.88667	0.44652	4	0.678329	3	3	11.78326	16.05146	1.855660	0.700364
1 nM LL vs. 33 nM LL	72.02000	45.23000	1.79908	4	0.146396	3	3	11.78326	22.94295	3.791121	0.417439
1 nM LL vs. 100 nM LL	72.02000	42.59667	2.00929	4	0.114892	3	3	11.78326	22.46032	3.633297	0.431658
3 nM LL vs. 10 nM LL	71.64000	66.88667	0.38843	4	0.717484	3	3	13.84192	16.05146	1.344735	0.852975
3 nM LL vs. 33 nM LL	71.64000	45.23000	1.70716	4	0.162982	3	3	13.84192	22.94295	2.747299	0.533718
3 nM LL vs. 100 nM LL	71.64000	42.59667	1.90670	4	0.129237	3	3	13.84192	22.46032	2.632929	0.550520
10 nM LL vs. 33 nM LL	66.88667	45.23000	1.33963	4	0.251410	3	3	16.05146	22.94295	2.043004	0.657245
10 nM LL vs. 100 nM LL	66.88667	42.59667	1.52397	4	0.202192	3	3	16.05146	22.46032	1.957954	0.676143
33 nM LL vs. 100 nM LL	45.23000	42.59667	0.14206	4	0.893901	3	3	22.94295	22.46032	1.043438	0.978743

Table S22. continuation

Group 1 vs. Group 2	T-test for Independent Samples (Paclitaxel_FACS_Detroit562_non-targeting siRNA) Note: Variables were treated as independent samples										
	Mean Group 1	Mean Group 2	t-value	df	p	Valid N Group 1	Valid N Group 2	Std.Dev. Group 1	Std.Dev. Group 2	F-ratio Variances	p Variances
Control LR vs. 1 nM LR	7.24000	7.22000	0.00555	4	0.995835	3	3	4.429684	4.391230	1.017591	0.991281
Control LR vs. 3 nM LR	7.24000	7.34333	-0.02974	4	0.977699	3	3	4.429684	4.073577	1.182479	0.916389
Control LR vs. 10 nM LR	7.24000	7.92333	-0.17774	4	0.867562	3	3	4.429684	4.971683	1.259683	0.885080
Control LR vs. 33 nM LR	7.24000	12.87667	-1.33726	4	0.252115	3	3	4.429684	5.803312	1.716352	0.736282
Control LR vs. 100 nM LR	7.24000	14.01667	-1.85373	4	0.137395	3	3	4.429684	4.524404	1.043223	0.978845
1 nM LR vs. 3 nM LR	7.22000	7.34333	-0.03566	4	0.973259	3	3	4.391230	4.073577	1.162038	0.925053
1 nM LR vs. 10 nM LR	7.22000	7.92333	-0.18365	4	0.863221	3	3	4.391230	4.971683	1.281842	0.876485
1 nM LR vs. 33 nM LR	7.22000	12.87667	-1.34630	4	0.249439	3	3	4.391230	5.803312	1.746544	0.728188
1 nM LR vs. 100 nM LR	7.22000	14.01667	-1.86711	4	0.135282	3	3	4.391230	4.524404	1.061574	0.970132
3 nM LR vs. 10 nM LR	7.34333	7.92333	-0.15630	4	0.883370	3	3	4.073577	4.971683	1.489549	0.803358
3 nM LR vs. 33 nM LR	7.34333	12.87667	-1.35171	4	0.247851	3	3	4.073577	5.803312	2.029551	0.660164
3 nM LR vs. 100 nM LR	7.34333	14.01667	-1.89857	4	0.130454	3	3	4.073577	4.524404	1.233590	0.895419
10 nM LR vs. 33 nM LR	7.92333	12.87667	-1.12271	4	0.324393	3	3	4.971683	5.803312	1.362527	0.846551
10 nM LR vs. 100 nM LR	7.92333	14.01667	-1.57002	4	0.191498	3	3	4.971683	4.524404	1.207492	0.906006
33 nM LR vs. 100 nM LR	12.87667	14.01667	-0.26833	4	0.801714	3	3	5.803312	4.524404	1.645239	0.756075

Table S22. continuation

Group 1 vs. Group 2	T-test for Independent Samples (Paclitaxel_FACS_Detroit562_GJA1 siRNA) Note: Variables were treated as independent samples										
	Mean Group 1	Mean Group 2	t-value	df	p	Valid N Group 1	Valid N Group 2	Std.Dev. Group 1	Std.Dev. Group 2	F-ratio Variances	p Variances
Control UL vs. 1 nM UL	1.940000	1.653333	0.43541	4	0.685733	3	3	0.693109	0.905557	1.70698	0.738831
Control UL vs. 3 nM UL	1.940000	1.676667	0.39323	4	0.714205	3	3	0.693109	0.930018	1.80044	0.714172
Control UL vs. 10 nM UL	1.940000	3.323333	-1.67326	4	0.169590	3	3	0.693109	1.253010	3.26818	0.468584
Control UL vs. 33 nM UL	1.940000	6.770000	-2.61738	4	0.058961	3	3	0.693109	3.120192	20.26561	0.094049
Control UL vs. 100 nM UL	1.940000	8.013333	-2.88508	4	0.044784	3	3	0.693109	3.579628	26.67305	0.072272
1 nM UL vs. 3 nM UL	1.653333	1.676667	-0.03113	4	0.976654	3	3	0.905557	0.930018	1.05475	0.973353

1 nM UL vs. 10 nM UL	1.653333	3.323333	-1.87099	4	0.134676	3	3	0.905557	1.253010	1.91460	0.686201
1 nM UL vs. 33 nM UL	1.653333	6.770000	-2.72776	4	0.052564	3	3	0.905557	3.120192	11.87220	0.155374
1 nM UL vs. 100 nM UL	1.653333	8.013333	-2.98339	4	0.040603	3	3	0.905557	3.579628	15.62587	0.120294
3 nM UL vs. 10 nM UL	1.676667	3.323333	-1.82776	4	0.141595	3	3	0.930018	1.253010	1.81521	0.710427
3 nM UL vs. 33 nM UL	1.676667	6.770000	-2.70956	4	0.053562	3	3	0.930018	3.120192	11.25590	0.163187
3 nM UL vs. 100 nM UL	1.676667	8.013333	-2.96756	4	0.041244	3	3	0.930018	3.579628	14.81471	0.126465
10 nM UL vs. 33 nM UL	3.323333	6.770000	-1.77547	4	0.150477	3	3	1.253010	3.120192	6.20089	0.277744
10 nM UL vs. 100 nM UL	3.323333	8.013333	-2.14189	4	0.098878	3	3	1.253010	3.579628	8.16144	0.218306
33 nM UL vs. 100 nM UL	6.770000	8.013333	-0.45350	4	0.673700	3	3	3.120192	3.579628	1.31617	0.863493

Table S22. continuation

Group 1 vs. Group 2	T-test for Independent Samples (Paclitaxel_FACS_Detroit562_GJA1 siRNA) Note: Variables were treated as independent samples										
	Mean Group 1	Mean Group 2	t-value	df	p	Valid N Group 1	Valid N Group 2	Std.Dev. Group 1	Std.Dev. Group 2	F-ratio Variances	p Variances
Control UR vs. 1 nM UR	11.43667	11.47000	-0.00807	4	0.993950	3	3	3.26462	6.36929	3.80643	0.416109
Control UR vs. 3 nM UR	11.43667	12.17667	-0.28583	4	0.789196	3	3	3.26462	3.07403	1.12785	0.939918
Control UR vs. 10 nM UR	11.43667	11.76667	-0.09885	4	0.926012	3	3	3.26462	4.77245	2.13706	0.637539
Control UR vs. 33 nM UR	11.43667	24.59667	-2.09898	4	0.103772	3	3	3.26462	10.35711	10.06496	0.180751
Control UR vs. 100 nM UR	11.43667	27.35667	-1.97021	4	0.120141	3	3	3.26462	13.60951	17.37880	0.108821
1 nM UR vs. 3 nM UR	11.47000	12.17667	-0.17307	4	0.871003	3	3	6.36929	3.07403	4.29307	0.377853
1 nM UR vs. 10 nM UR	11.47000	11.76667	-0.06456	4	0.951621	3	3	6.36929	4.77245	1.78115	0.719127
1 nM UR vs. 33 nM UR	11.47000	24.59667	-1.86992	4	0.134844	3	3	6.36929	10.35711	2.64420	0.548817
1 nM UR vs. 100 nM UR	11.47000	27.35667	-1.83124	4	0.141025	3	3	6.36929	13.60951	4.56565	0.359347
3 nM UR vs. 10 nM UR	12.17667	11.76667	0.12510	4	0.906483	3	3	3.07403	4.77245	2.41028	0.586463
3 nM UR vs. 33 nM UR	12.17667	24.59667	-1.99118	4	0.117292	3	3	3.07403	10.35711	11.35172	0.161921
3 nM UR vs. 100 nM UR	12.17667	27.35667	-1.88445	4	0.132598	3	3	3.07403	13.60951	19.60062	0.097084
10 nM UR vs. 33 nM UR	11.76667	24.59667	-1.94867	4	0.123145	3	3	4.77245	10.35711	4.70972	0.350280
10 nM UR vs. 100 nM UR	11.76667	27.35667	-1.87232	4	0.134470	3	3	4.77245	13.60951	8.13210	0.219008
33 nM UR vs. 100 nM UR	24.59667	27.35667	-0.27952	4	0.793703	3	3	10.35711	13.60951	1.72666	0.733497

Table S22. continuation

Group 1 vs. Group 2	T-test for Independent Samples (Paclitaxel_FACS_Detroit562_GJA1 siRNA) Note: Variables were treated as independent samples										
	Mean Group 1	Mean Group 2	t-value	df	p	Valid N Group 1	Valid N Group 2	Std.Dev. Group 1	Std.Dev. Group 2	F-ratio Variances	p Variances
Control LL vs. 1 nM LL	80.89333	81.47667	-0.08678	4	0.935019	3	3	7.05360	9.26338	1.724717	0.734021
Control LL vs. 3 nM LL	80.89333	80.55000	0.06221	4	0.953383	3	3	7.05360	6.45238	1.195037	0.911146
Control LL vs. 10 nM LL	80.89333	77.84000	0.43402	4	0.686656	3	3	7.05360	9.93570	1.984154	0.670207
Control LL vs. 33 nM LL	80.89333	55.18333	2.42240	4	0.072575	3	3	7.05360	16.97594	5.792239	0.294454
Control LL vs. 100 nM LL	80.89333	49.74000	2.69136	4	0.054581	3	3	7.05360	18.76726	7.079136	0.247551
1 nM LL vs. 3 nM LL	81.47667	80.55000	0.14218	4	0.893815	3	3	9.26338	6.45238	2.061101	0.653360
1 nM LL vs. 10 nM LL	81.47667	77.84000	0.46370	4	0.666975	3	3	9.26338	9.93570	1.150423	0.930049
1 nM LL vs. 33 nM LL	81.47667	55.18333	2.35491	4	0.078095	3	3	9.26338	16.97594	3.358371	0.458887
1 nM LL vs. 100 nM LL	81.47667	49.74000	2.62648	4	0.058401	3	3	9.26338	18.76726	4.104521	0.391810
3 nM LL vs. 10 nM LL	80.55000	77.84000	0.39621	4	0.712179	3	3	6.45238	9.93570	2.371139	0.593271
3 nM LL vs. 33 nM LL	80.55000	55.18333	2.41929	4	0.072819	3	3	6.45238	16.97594	6.921943	0.252463
3 nM LL vs. 100 nM LL	80.55000	49.74000	2.68900	4	0.054715	3	3	6.45238	18.76726	8.459833	0.211420
10 nM LL vs. 33 nM LL	77.84000	55.18333	1.99506	4	0.116773	3	3	9.93570	16.97594	2.919248	0.510302
10 nM LL vs. 100 nM LL	77.84000	49.74000	2.29199	4	0.083673	3	3	9.93570	18.76726	3.567835	0.437844
33 nM LL vs. 100 nM LL	55.18333	49.74000	0.37257	4	0.728372	3	3	16.97594	18.76726	1.222176	0.900019

Table S22. continuation

Group 1 vs. Group 2	T-test for Independent Samples (Paclitaxel_FACS_Detroit562_GJA1 siRNA) Note: Variables were treated as independent samples										
	Mean Group 1	Mean Group 2	t-value	df	p	Valid N Group 1	Valid N Group 2	Std.Dev. Group 1	Std.Dev. Group 2	F-ratio Variances	p Variances
Control LR vs. 1 nM LR	5.73000	5.40000	0.15409	4	0.885001	3	3	3.098500	2.039338	2.308471	0.604509
Control LR vs. 3 nM LR	5.73000	5.59667	0.05660	4	0.957581	3	3	3.098500	2.655039	1.361950	0.846758
Control LR vs. 10 nM LR	5.73000	7.06667	-0.46109	4	0.668690	3	3	3.098500	3.951004	1.625968	0.761624
Control LR vs. 33 nM LR	5.73000	13.45000	-2.48845	4	0.067596	3	3	3.098500	4.390068	2.007427	0.665020
Control LR vs. 100 nM LR	5.73000	14.89000	-3.85605	4	0.018208	3	3	3.098500	2.707046	1.310121	0.865755
1 nM LR vs. 3 nM LR	5.40000	5.59667	-0.10175	4	0.923853	3	3	2.039338	2.655039	1.694975	0.742122

1 nM LR vs. 10 nM LR	5.40000	7.06667	-0.64925	4	0.551572	3	3	2.039338	3.951004	3.753501	0.420743
1 nM LR vs. 33 nM LR	5.40000	13.45000	-2.88042	4	0.044994	3	3	2.039338	4.390068	4.634086	0.354982
1 nM LR vs. 100 nM LR	5.40000	14.89000	-4.84980	4	0.008341	3	3	2.039338	2.707046	1.762028	0.724106
3 nM LR vs. 10 nM LR	5.59667	7.06667	-0.53487	4	0.621087	3	3	2.655039	3.951004	2.214487	0.622183
3 nM LR vs. 33 nM LR	5.59667	13.45000	-2.65128	4	0.056906	3	3	2.655039	4.390068	2.734014	0.535617
3 nM LR vs. 100 nM LR	5.59667	14.89000	-4.24515	4	0.013209	3	3	2.655039	2.707046	1.039560	0.980604
10 nM LR vs. 33 nM LR	7.06667	13.45000	-1.87198	4	0.134523	3	3	3.951004	4.390068	1.234604	0.895013
10 nM LR vs. 100 nM LR	7.06667	14.89000	-2.82924	4	0.047382	3	3	3.951004	2.707046	2.130216	0.638934
33 nM LR vs. 100 nM LR	13.45000	14.89000	-0.48359	4	0.653958	3	3	4.390068	2.707046	2.629972	0.550968

Table S22. continuation

Group 1 vs. Group 2	T-test for Independent Samples (Paclitaxel_FACS_Detroit562_non-targeting siRNA + GJA1 siRNA) Note: Variables were treated as independent samples										
	Mean Group 1	Mean Group 2	t-value	df	p	N 1	N 2	Std.Dev. Group 1	Std.Dev. Group 2	F-ratio Variances	p Variances
siControl Control UL vs. siGJA1 Control UL	3.933333	1.940000	2.111177	4	0.102355	3	3	1.481227	0.693109	4.567097	0.359254
siControl Control UR vs. siGJA1 Control UR	18.24000	11.43667	1.439730	4	0.223358	3	3	7.505405	3.264618	5.285467	0.318194
siControl Control LL vs. siGJA1 Control LL	70.58333	80.89333	-1.17947	4	0.303570	3	3	13.39673	7.053597	3.607252	0.434098
siControl Control LR vs. siGJA1 Control LR	7.240000	5.730000	0.483812	4	0.653813	3	3	4.429684	3.098500	2.043820	0.657069
siControl 1 nM UL vs. siGJA1 1 nM UL	3.660000	1.653333	1.867756	4	0.135182	3	3	1.625669	0.905557	3.222796	0.473620
siControl 1 nM UR vs. siGJA1 1 nM UR	17.10000	11.47000	1.122926	4	0.324310	3	3	5.902821	6.369294	1.164296	0.924088
siControl 1 nM LL vs. siGJA1 1 nM LL	72.02000	81.47667	-1.09280	4	0.335884	3	3	11.78326	9.263381	1.618049	0.763928
siControl 1 nM LR vs. siGJA1 1 nM LR	7.220000	5.400000	0.651083	4	0.550503	3	3	4.391230	2.039338	4.636539	0.354828
siControl 3 nM UL vs. siGJA1 3 nM UL	3.650000	1.676667	1.630050	4	0.178424	3	3	1.879282	0.930018	4.083205	0.393453
siControl 3 nM UR vs. siGJA1 3 nM UR	17.36667	12.17667	1.051679	4	0.352283	3	3	7.975715	3.074026	6.731693	0.258676
siControl 3 nM LL vs. siGJA1 3 nM LL	71.64000	80.55000	-1.01052	4	0.369409	3	3	13.84192	6.452379	4.602065	0.357011
siControl 3 nM LR vs. siGJA1 3 nM LR	7.343333	5.596667	0.622181	4	0.567532	3	3	4.073577	2.655039	2.354020	0.596299
siControl 10 nM UL vs. siGJA1 10 nM UL	4.833333	3.323333	0.877350	4	0.429832	3	3	2.704891	1.253010	4.660050	0.353354
siControl 10 nM UR vs. siGJA1 10 nM UR	20.35667	11.76667	1.515474	4	0.204232	3	3	8.579571	4.772445	3.231835	0.472608
siControl 10 nM LL vs. siGJA1 10 nM LL	66.88667	77.84000	-1.00498	4	0.371768	3	3	16.05146	9.935698	2.609951	0.554024
siControl 10 nM LR vs. siGJA1 10 nM LR	7.923333	7.066667	0.233652	4	0.826726	3	3	4.971683	3.951004	1.583405	0.774172

siControl 33 nM UL vs. siGJA1 33 nM UL	8.270000	6.770000	0.673919	4	0.537302	3	3	2.264244	3.120192	1.898962	0.689902
siControl 33 nM UR vs. siGJA1 33 nM UR	33.62000	24.59667	0.860968	4	0.437805	3	3	14.90806	10.35711	2.071885	0.651066
siControl 33 nM LL vs. siGJA1 33 nM LL	45.23000	55.18333	-0.604042	4	0.578401	3	3	22.94295	16.97594	1.826546	0.707577
siControl 33 nM LR vs. siGJA1 33 nM LR	12.87667	13.45000	-0.136468	4	0.898044	3	3	5.803312	4.390068	1.747468	0.727943

Table S22. continuation

siControl 100 nM UL vs. siGJA1 100 nM UL	8.610000	8.013333	0.246578	4	0.817372	3	3	2.180000	3.579628	2.696266	0.541087
siControl 100 nM UR vs. siGJA1 100 nM UR	34.77333	27.35667	0.609535	4	0.575095	3	3	16.09174	13.60951	1.398045	0.834013
siControl 100 nM LL vs. siGJA1 100 nM LL	42.59667	49.74000	-0.422721	4	0.694234	3	3	22.46032	18.76726	1.432287	0.822271
siControl 100 nM LR vs. siGJA1 100 nM LR	14.01667	14.89000	-0.286900	4	0.788437	3	3	4.524404	2.707046	2.793389	0.527233

Table S23. Changes in the paclitaxel-induced apoptosis of FaDu after knocking down Cx43. Annexin V-FLUOS/PI (Ann/PI)-stained HNSCC cells were analyzed by FACS after 48 h of treatment with paclitaxel at different concentrations. Live cells are presented by the Ann-/PI- fraction (LL), apoptotic cells by the Ann+/PI- fraction (LR), secondary necrotic cells by the Ann+/PI+ fraction (UR) and primary necrotic cells are detected in the Ann-/PI+ fraction (UL). (Figure S2).

Concentration (nM)		Paclitaxel-induced apoptosis of non-targeting siRNA treated FaDu cell (%)				
		Sample 1	Sample 2	Sample 3	Mean	SD
Control	UL	0.99	0.8	0.39	0.7	0.306649
	UR	6.47	18.53	10.77	11.9	6.112163
	LL	89.52	78.09	86.38	84.7	5.905204
	LR	3.02	2.57	2.46	2.7	0.296704
1	UL	0.76	0.89	0.57	0.7	0.160935
	UR	4.71	19.58	8.68	11.0	7.699435
	LL	91.78	76.06	88.56	85.5	8.303983
	LR	2.76	3.47	2.19	2.8	0.641275
3	UL	0.62	0.97	0.43	0.7	0.273922
	UR	5.45	21.33	10	12.3	8.177671
	LL	89.96	73.72	85.64	83.1	8.411167
	LR	3.97	3.98	3.93	4.0	0.026458
10	UL	1.37	1.57	1.02	1.3	0.278388
	UR	6.98	26.64	11.54	15.1	10.29012
	LL	85.46	62.9	80.28	76.2	11.81701
	LR	6.19	8.89	7.16	7.4	1.367711
33	UL	3.68	3.08	2.64	3.1	0.522047
	UR	8.42	32.7	20.46	20.5	12.14014

	LL	77.2	43.78	58.06	59.7	16.76879
	LR	10.71	20.45	18.84	16.7	5.221057
100	UL	7.78	3.66	7.86	6.4	2.40211
	UR	11.18	45.74	25.84	27.6	17.34608
	LL	59.06	28.6	47.9	45.2	15.41021
	LR	21.97	21.99	18.4	20.8	2.066938

Concentration (nM)		Paclitaxel-induced apoptosis of *GJA1* siRNA treated FaDu cell (%)				
		Sample 1	Sample 2	Sample 3	Mean	SD
Control	UL	0.45	0.42	0.64	0.50	0.119304
	UR	3.49	12.3	13.13	9.64	5.3422
	LL	93.77	84.16	84.22	87.38	5.531097
	LR	2.28	3.12	2.01	2.47	0.578878
1	UL	0.51	0.46	0.65	0.54	0.098489
	UR	5.56	13.97	9.16	9.56	4.219483
	LL	90.43	82.8	87.44	86.89	3.84462
	LR	3.49	2.77	2.75	3.00	0.421584
3	UL	0.76	0.71	0.61	0.69	0.076376
	UR	4.75	14.05	9.59	9.46	4.651294
	LL	91.6	81.22	86.28	86.37	5.190543
	LR	2.89	4.02	3.52	3.48	0.566245

Table S23. continuation

10	UL	0.6	0.99	0.75	0.78	0.196723
	UR	3.74	19.1	8.73	10.52	7.83546
	LL	92.1	73.4	83.63	83.04	9.363794
	LR	3.56	6.51	6.89	5.65	1.822809
33	UL	1.11	0.88	0.88	0.96	0.132791
	UR	6.05	14.76	11.43	10.75	4.395024
	LL	87.21	73.93	79.88	80.34	6.65194
	LR	5.63	10.43	7.81	7.96	2.403359
100	UL	1.58	0.98	0.77	1.11	0.420357
	UR	5.98	17.16	13.67	12.27	5.719974
	LL	82.66	67.51	75.07	75.08	7.575005
	LR	9.78	14.35	10.49	11.54	2.459289

Table S24. Changes in the paclitaxel-induced apoptosis of FaDu after knocking down Cx43. Annexin V-FLUOS/PI (Ann/PI)-stained HNSCC cells were analyzed by FACS after 48 h of treatment with paclitaxel at different concentrations. Live cells are presented by the Ann-/PI- fraction (LL), apoptotic cells by the Ann+/PI- fraction (LR), secondary necrotic cells by the Ann+/PI+ fraction (UR) and primary necrotic cells are detected in the Ann-/PI+ fraction (UL). Statistical analysis was performed by Student's t-test, the cell fractions in all concentration were compared to negative control fractions in non-targeting siRNA or *GJA1* siRNA treated samples. The cell fractions in *GJA1* siRNA treated samples were also compared to cell fractions in non-targeting siRNA treated samples.
Red color indicate if p < 0.05. (Figure S2).

Group 1 vs. Group 2	T-test for Independent Samples (Paclitaxel_FACS_FaDu_non-targeting siRNA) Note: Variables were treated as independent samples									
	Mean Group 1	Mean Group 2	t-value	df	p	Valid N Group 1	Valid N Group 2	Std.Dev. Group 1	Std.Dev. Group 2	F-ratio Variances	p Variances
Control UL vs. 1 nM UL	0.726667	0.740000	-0.06669	4	0.950032	3	3	0.306649	0.160935	3.6306	0.431907
Control UL vs. 3 nM UL	0.726667	0.673333	0.22466	4	0.833252	3	3	0.306649	0.273922	1.2532	0.887618
Control UL vs. 10 nM UL	0.726667	1.320000	-2.48133	4	0.068114	3	3	0.306649	0.278388	1.2133	0.903614
Control UL vs. 33 nM UL	0.726667	3.133333	-6.88494	4	0.002332	3	3	0.306649	0.522047	2.8983	0.513049
Control UL vs. 100 nM UL	0.726667	6.433333	-4.08169	4	0.015078	3	3	0.306649	2.402110	61.3626	0.032070
1 nM UL vs. 3 nM UL	0.740000	0.673333	0.36346	4	0.734659	3	3	0.160935	0.273922	2.8970	0.513210
1 nM UL vs. 10 nM UL	0.740000	1.320000	-3.12412	4	0.035385	3	3	0.160935	0.278388	2.9923	0.500967
1 nM UL vs. 33 nM UL	0.740000	3.133333	-7.58822	4	0.001618	3	3	0.160935	0.522047	10.5225	0.173573
1 nM UL vs. 100 nM UL	0.740000	6.433333	-4.09602	4	0.014902	3	3	0.160935	2.402110	222.7851	0.008937
3 nM UL vs. 10 nM UL	0.673333	1.320000	-2.86787	4	0.045567	3	3	0.273922	0.278388	1.0329	0.983829
3 nM UL vs. 33 nM UL	0.673333	3.133333	-7.22731	4	0.001944	3	3	0.273922	0.522047	3.6322	0.431764
3 nM UL vs. 100 nM UL	0.673333	6.433333	-4.12653	4	0.014535	3	3	0.273922	2.402110	76.9009	0.025674
10 nM UL vs. 33 nM UL	1.320000	3.133333	-5.30864	4	0.006052	3	3	0.278388	0.522047	3.5166	0.442815
10 nM UL vs. 100 nM UL	1.320000	6.433333	-3.66247	4	0.021533	3	3	0.278388	2.402110	74.4533	0.026506
33 nM UL vs. 100 nM UL	3.133333	6.433333	-2.32520	4	0.080675	3	3	0.522047	2.402110	21.1722	0.090203

Table S24. continuation

Group 1 vs. Group 2	T-test for Independent Samples (Paclitaxel_FACS_FaDu_non-targeting siRNA) Note: Variables were treated as independent samples									
	Mean Group 1	Mean Group 2	t-value	df	p	Valid N Group 1	Valid N Group 2	Std.Dev. Group 1	Std.Dev. Group 2	F-ratio Variances	p Variances
Control UR vs. 1 nM UR	11.92333	10.99000	0.16444	4	0.877357	3	3	6.11216	7.69944	1.586821	0.773150
Control UR vs. 3 nM UR	11.92333	12.26000	-0.05712	4	0.957192	3	3	6.11216	8.17767	1.790068	0.716829

Control UR vs. 10 nM UR	11.92333	15.05333	-0.45297	4	0.674056	3	3	6.11216	10.29012	2.834333	0.521603
Control UR vs. 33 nM UR	11.92333	20.52667	-1.09634	4	0.334504	3	3	6.11216	12.14014	3.945094	0.404441
Control UR vs. 100 nM UR	11.92333	27.58667	-1.47513	4	0.214205	3	3	6.11216	17.34608	8.054024	0.220896
1 nM UR vs. 3 nM UR	10.99000	12.26000	-0.19584	4	0.854279	3	3	7.69944	8.17767	1.128084	0.939812
1 nM UR vs. 10 nM UR	10.99000	15.05333	-0.54762	4	0.613074	3	3	7.69944	10.29012	1.786171	0.717831
1 nM UR vs. 33 nM UR	10.99000	20.52667	-1.14901	4	0.314585	3	3	7.69944	12.14014	2.486162	0.573697
1 nM UR vs. 100 nM UR	10.99000	27.58667	-1.51471	4	0.204417	3	3	7.69944	17.34608	5.075572	0.329187
3 nM UR vs. 10 nM UR	12.26000	15.05333	-0.36810	4	0.731454	3	3	8.17767	10.29012	1.583367	0.774184
3 nM UR vs. 33 nM UR	12.26000	20.52667	-0.97819	4	0.383367	3	3	8.17767	12.14014	2.203880	0.624243
3 nM UR vs. 100 nM UR	12.26000	27.58667	-1.38429	4	0.238490	3	3	8.17767	17.34608	4.499285	0.363684
10 nM UR vs. 33 nM UR	15.05333	20.52667	-0.59569	4	0.583450	3	3	10.29012	12.14014	1.391895	0.836157
10 nM UR vs. 100 nM UR	15.05333	27.58667	-1.07634	4	0.342362	3	3	10.29012	17.34608	2.841594	0.520617
33 nM UR vs. 100 nM UR	20.52667	27.58667	-0.57756	4	0.594515	3	3	12.14014	17.34608	2.041529	0.657564

Table S24. continuation

Group 1 vs. Group 2	T-test for Independent Samples (Paclitaxel_FACS_FaDu_non-targeting siRNA) Note: Variables were treated as independent samples										
	Mean Group 1	Mean Group 2	t-value	df	p	Valid N Group 1	Valid N Group 2	Std.Dev. Group 1	Std.Dev. Group 2	F-ratio Variances	p Variances
Control LL vs. 1 nM LL	84.66333	85.46667	-0.13655	4	0.897981	3	3	5.90520	8.30398	1.977439	0.671718
Control LL vs. 3 nM LL	84.66333	83.10667	0.26235	4	0.806007	3	3	5.90520	8.41117	2.028816	0.660324
Control LL vs. 10 nM LL	84.66333	76.21333	1.10791	4	0.330034	3	3	5.90520	11.81701	4.004474	0.399642
Control LL vs. 33 nM LL	84.66333	59.68000	2.43402	4	0.071669	3	3	5.90520	16.76879	8.063689	0.220661
Control LL vs. 100 nM LL	84.66333	45.18667	4.14325	4	0.014339	3	3	5.90520	15.41021	6.810002	0.256082
1 nM LL vs. 3 nM LL	85.46667	83.10667	0.34583	4	0.746890	3	3	8.30398	8.41117	1.025982	0.987176
1 nM LL vs. 10 nM LL	85.46667	76.21333	1.10970	4	0.329347	3	3	8.30398	11.81701	2.025081	0.661139
1 nM LL vs. 33 nM LL	85.46667	59.68000	2.38688	4	0.075423	3	3	8.30398	16.76879	4.077845	0.393868
1 nM LL vs. 100 nM LL	85.46667	45.18667	3.98551	4	0.016326	3	3	8.30398	15.41021	3.443849	0.450060
3 nM LL vs. 10 nM LL	83.10667	76.21333	0.82315	4	0.456667	3	3	8.41117	11.81701	1.973798	0.672541
3 nM LL vs. 33 nM LL	83.10667	59.68000	2.16290	4	0.096575	3	3	8.41117	16.76879	3.974578	0.402044

3 nM LL vs. 100 nM LL	83.10667	45.18667	3.74108	4	0.020102	3	3	8.41117	15.41021	3.356638	0.459070
10 nM LL vs. 33 nM LL	76.21333	59.68000	1.39594	4	0.235228	3	3	11.81701	16.76879	2.013670	0.663643
10 nM LL vs. 100 nM LL	76.21333	45.18667	2.76731	4	0.050470	3	3	11.81701	15.41021	1.700599	0.740577
33 nM LL vs. 100 nM LL	59.68000	45.18667	1.10226	4	0.332209	3	3	16.76879	15.41021	1.184095	0.915711

Table S24. continuation

Group 1 vs. Group 2	T-test for Independent Samples (Paclitaxel_FACS_FaDu_non-targeting siRNA) Note: Variables were treated as independent samples									
	Mean Group 1	Mean Group 2	t-value	df	p	Valid N Group 1	Valid N Group 2	Std.Dev. Group 1	Std.Dev. Group 2	F-ratio Variances	p Variances
Control LR vs. 1 nM LR	2.68333	2.80667	-0.3023	4	0.777472	3	3	0.296704	0.641275	4.67	0.352651
Control LR vs. 3 nM LR	2.68333	3.96000	-7.4233	4	0.001758	3	3	0.296704	0.026458	125.76	0.015778
Control LR vs. 10 nM LR	2.68333	7.41333	-5.8538	4	0.004249	3	3	0.296704	1.367711	21.25	0.089891
Control LR vs. 33 nM LR	2.68333	16.66667	-4.6314	4	0.009797	3	3	0.296704	5.221057	309.65	0.006438
Control LR vs. 100 nM LR	2.68333	20.78667	-15.0163	4	0.000115	3	3	0.296704	2.066938	48.53	0.040380
1 nM LR vs. 3 nM LR	2.80667	3.96000	-3.1124	4	0.035787	3	3	0.641275	0.026458	587.48	0.003399
1 nM LR vs. 10 nM LR	2.80667	7.41333	-5.2820	4	0.006162	3	3	0.641275	1.367711	4.55	0.360436
1 nM LR vs. 33 nM LR	2.80667	16.66667	-4.5637	4	0.010310	3	3	0.641275	5.221057	66.29	0.029723
1 nM LR vs. 100 nM LR	2.80667	20.78667	-14.3902	4	0.000136	3	3	0.641275	2.066938	10.39	0.175611
3 nM LR vs. 10 nM LR	3.96000	7.41333	-4.3724	4	0.011945	3	3	0.026458	1.367711	2672.33	0.000748
3 nM LR vs. 33 nM LR	3.96000	16.66667	-4.2153	4	0.013529	3	3	0.026458	5.221057	38942.05	0.000051
3 nM LR vs. 100 nM LR	3.96000	20.78667	-14.0992	4	0.000147	3	3	0.026458	2.066938	6103.19	0.000328
10 nM LR vs. 33 nM LR	7.41333	16.66667	-2.9695	4	0.041163	3	3	1.367711	5.221057	14.57	0.128433
10 nM LR vs. 100 nM LR	7.41333	20.78667	-9.3458	4	0.000730	3	3	1.367711	2.066938	2.28	0.609042
33 nM LR vs. 100 nM LR	16.66667	20.78667	-1.2708	4	0.272672	3	3	5.221057	2.066938	6.38	0.270980

Table S24. continuation

Group 1 vs. Group 2	T-test for Independent Samples (Paclitaxel_FACS_FaDu_GJA1 siRNA) Note: Variables were treated as independent samples									
	Mean Group 1	Mean Group 2	t-value	df	p	Valid N Group 1	Valid N Group 2	Std.Dev. Group 1	Std.Dev. Group 2	F-ratio Variances	p Variances
Control UL vs. 1 nM UL	0.503333	0.540000	-0.41052	4	0.702465	3	3	0.119304	0.098489	1.46735	0.810585
Control UL vs. 3 nM UL	0.503333	0.693333	-2.32315	4	0.080857	3	3	0.119304	0.076376	2.44000	0.581395

Control UL vs. 10 nM UL	0.503333	0.780000	-2.08283	4	0.105685	3	3	0.119304	0.196723	2.71897	0.537783
Control UL vs. 33 nM UL	0.503333	0.956667	-4.39855	4	0.011704	3	3	0.119304	0.132791	1.23888	0.893305
Control UL vs. 100 nM UL	0.503333	1.110000	-2.40475	4	0.073974	3	3	0.119304	0.420357	12.41452	0.149092
1 nM UL vs. 3 nM UL	0.540000	0.693333	-2.13091	4	0.100106	3	3	0.098489	0.076376	1.66286	0.751073
1 nM UL vs. 10 nM UL	0.540000	0.780000	-1.88951	4	0.131825	3	3	0.098489	0.196723	3.98969	0.400826
1 nM UL vs. 33 nM UL	0.540000	0.956667	-4.36519	4	0.012013	3	3	0.098489	0.132791	1.81787	0.709756
1 nM UL vs. 100 nM UL	0.540000	1.110000	-2.28672	4	0.084161	3	3	0.098489	0.420357	18.21649	0.104077
3 nM UL vs. 10 nM UL	0.693333	0.780000	-0.71133	4	0.516164	3	3	0.076376	0.196723	6.63429	0.261976
3 nM UL vs. 33 nM UL	0.693333	0.956667	-2.97742	4	0.040843	3	3	0.076376	0.132791	3.02286	0.497159
3 nM UL vs. 100 nM UL	0.693333	1.110000	-1.68919	4	0.166451	3	3	0.076376	0.420357	30.29143	0.063915
10 nM UL vs. 33 nM UL	0.780000	0.956667	-1.28924	4	0.266817	3	3	0.196723	0.132791	2.19471	0.626036
10 nM UL vs. 100 nM UL	0.780000	1.110000	-1.23155	4	0.285571	3	3	0.196723	0.420357	4.56589	0.359331
33 nM UL vs. 100 nM UL	0.956667	1.110000	-0.60245	4	0.579359	3	3	0.132791	0.420357	10.02079	0.181475

Table S24. continuation

Group 1 vs. Group 2	T-test for Independent Samples (Paclitaxel_FACS_FaDu_GJA1 siRNA) Note: Variables were treated as independent samples										
	Mean Group 1	Mean Group 2	t-value	df	p	Valid N Group 1	Valid N Group 2	Std.Dev. Group 1	Std.Dev. Group 2	F-ratio Variances	p Variances
Control UR vs. 1 nM UR	9.64000	9.56333	0.019506	4	0.985371	3	3	5.342200	4.219483	1.602957	0.768357
Control UR vs. 3 nM UR	9.64000	9.46333	0.043199	4	0.967613	3	3	5.342200	4.651294	1.319146	0.862387
Control UR vs. 10 nM UR	9.64000	10.52333	-0.161333	4	0.879652	3	3	5.342200	7.835460	2.151239	0.634671
Control UR vs. 33 nM UR	9.64000	10.74667	-0.277085	4	0.795445	3	3	5.342200	4.395024	1.477467	0.807276
Control UR vs. 100 nM UR	9.64000	12.27000	-0.582020	4	0.591780	3	3	5.342200	5.719974	1.146431	0.931779
1 nM UR vs. 3 nM UR	9.56333	9.46333	0.027580	4	0.979318	3	3	4.219483	4.651294	1.215148	0.902874
1 nM UR vs. 10 nM UR	9.56333	10.52333	-0.186842	4	0.860879	3	3	4.219483	7.835460	3.448344	0.449606
1 nM UR vs. 33 nM UR	9.56333	10.74667	-0.336405	4	0.753473	3	3	4.219483	4.395024	1.084936	0.959262
1 nM UR vs. 100 nM UR	9.56333	12.27000	-0.659560	4	0.545576	3	3	4.219483	5.719974	1.837679	0.704801
3 nM UR vs. 10 nM UR	9.46333	10.52333	-0.201489	4	0.850148	3	3	4.651294	7.835460	2.837798	0.521132
3 nM UR vs. 33 nM UR	9.46333	10.74667	-0.347351	4	0.745834	3	3	4.651294	4.395024	1.120018	0.943388

3 nM UR vs. 100 nM UR	9.46333	12.27000	-0.659388	4	0.545676	3	3	4.651294	5.719974	1.512309	0.796080
10 nM UR vs. 33 nM UR	10.52333	10.74667	-0.043057	4	0.967719	3	3	7.835460	4.395024	3.178385	0.478654
10 nM UR vs. 100 nM UR	10.52333	12.27000	-0.311851	4	0.770733	3	3	7.835460	5.719974	1.876467	0.695297
33 nM UR vs. 100 nM UR	10.74667	12.27000	-0.365772	4	0.733058	3	3	4.395024	5.719974	1.693814	0.742442

Table S24. continuation

Group 1 vs. Group 2	T-test for Independent Samples (Paclitaxel_FACS_FaDu_GJA1 siRNA) Note: Variables were treated as independent samples										
	Mean Group 1	Mean Group 2	t-value	df	p	Valid N Group 1	Valid N Group 2	Std.Dev. Group 1	Std.Dev. Group 2	F-ratio Variances	p Variances
Control LL vs. 1 nM LL	87.38333	86.89000	0.12685	4	0.905179	3	3	5.531097	3.844620	2.069740	0.651521
Control LL vs. 3 nM LL	87.38333	86.36667	0.23215	4	0.827813	3	3	5.531097	5.190543	1.135526	0.936538
Control LL vs. 10 nM LL	87.38333	83.04333	0.69120	4	0.527459	3	3	5.531097	9.363794	2.866033	0.517326
Control LL vs. 33 nM LL	87.38333	80.34000	1.41016	4	0.231306	3	3	5.531097	6.651940	1.446352	0.817544
Control LL vs. 100 nM LL	87.38333	75.08000	2.27199	4	0.085539	3	3	5.531097	7.575005	1.875613	0.695504
1 nM LL vs. 3 nM LL	86.89000	86.36667	0.14033	4	0.895182	3	3	3.844620	5.190543	1.822715	0.708538
1 nM LL vs. 10 nM LL	86.89000	83.04333	0.65821	4	0.546359	3	3	3.844620	9.363794	5.931942	0.288519
1 nM LL vs. 33 nM LL	86.89000	80.34000	1.47662	4	0.213828	3	3	3.844620	6.651940	2.993573	0.500805
1 nM LL vs. 100 nM LL	86.89000	75.08000	2.40800	4	0.073714	3	3	3.844620	7.575005	3.882032	0.409666
3 nM LL vs. 10 nM LL	86.36667	83.04333	0.53765	4	0.619336	3	3	5.190543	9.363794	3.254454	0.470096
3 nM LL vs. 33 nM LL	86.36667	80.34000	1.23717	4	0.283691	3	3	5.190543	6.651940	1.642370	0.756896
3 nM LL vs. 100 nM LL	86.36667	75.08000	2.12890	4	0.100332	3	3	5.190543	7.575005	2.129807	0.639017
10 nM LL vs. 33 nM LL	83.04333	80.34000	0.40765	4	0.704403	3	3	9.363794	6.651940	1.981559	0.670790
10 nM LL vs. 100 nM LL	83.04333	75.08000	1.14520	4	0.315991	3	3	9.363794	7.575005	1.528051	0.791123
33 nM LL vs. 100 nM LL	80.34000	75.08000	0.90373	4	0.417245	3	3	6.651940	7.575005	1.296789	0.870781

Table S24. continuation

Group 1 vs. Group 2	T-test for Independent Samples (Paclitaxel_FACS_FaDu_GJA1 siRNA) Note: Variables were treated as independent samples										
	Mean Group 1	Mean Group 2	t-value	df	p	Valid N Group 1	Valid N Group 2	Std.Dev. Group 1	Std.Dev. Group 2	F-ratio Variances	p Variances
Control LR vs. 1 nM LR	2.47000	3.00333	-1.28995	4	0.266594	3	3	0.578878	0.421584	1.88541	0.693143
Control LR vs. 3 nM LR	2.47000	3.47667	-2.15319	4	0.097631	3	3	0.578878	0.566245	1.04512	0.977938

Control LR vs. 10 nM LR	2.47000	5.65333	-2.88295	4	0.044880	3	3	0.578878	1.822809	9.91535	0.183228
Control LR vs. 33 nM LR	2.47000	7.95667	-3.84419	4	0.018394	3	3	0.578878	2.403359	17.23704	0.109667
Control LR vs. 100 nM LR	2.47000	11.54000	-6.21797	4	0.003405	3	3	0.578878	2.459289	18.04864	0.104994
1 nM LR vs. 3 nM LR	3.00333	3.47667	-1.16132	4	0.310089	3	3	0.421584	0.566245	1.80401	0.713263
1 nM LR vs. 10 nM LR	3.00333	5.65333	-2.45329	4	0.070196	3	3	0.421584	1.822809	18.69449	0.101551
1 nM LR vs. 33 nM LR	3.00333	7.95667	-3.51608	4	0.024537	3	3	0.421584	2.403359	32.49887	0.059703
1 nM LR vs. 100 nM LR	3.00333	11.54000	-5.92584	4	0.004063	3	3	0.421584	2.459289	34.02907	0.057095
3 nM LR vs. 10 nM LR	3.47667	5.65333	-1.97518	4	0.119459	3	3	0.566245	1.822809	10.36272	0.176014
3 nM LR vs. 33 nM LR	3.47667	7.95667	-3.14260	4	0.034761	3	3	0.566245	2.403359	18.01476	0.105181
3 nM LR vs. 100 nM LR	3.47667	11.54000	-5.53412	4	0.005210	3	3	0.566245	2.459289	18.86298	0.100690
10 nM LR vs. 33 nM LR	5.65333	7.95667	-1.32259	4	0.256521	3	3	1.822809	2.403359	1.73842	0.730348
10 nM LR vs. 100 nM LR	5.65333	11.54000	-3.33076	4	0.029085	3	3	1.822809	2.459289	1.82027	0.709151
33 nM LR vs. 100 nM LR	7.95667	11.54000	-1.80493	4	0.145401	3	3	2.403359	2.459289	1.04708	0.976999

Table S24. continuation

Group 1 vs. Group 2	T-test for Independent Samples (Paclitaxel_FACS_FaDu_non-targeting siRNA + GJA1 siRNA) Note: Variables were treated as independent samples										
	Mean Group 1	Mean Group 2	t-value	df	p	N 1	N 2	Std.Dev. Group 1	Std.Dev. Group 2	F-ratio Variances	p Variances
siControl Control UL vs. siGJA1 Control UL	0.726667	0.503333	1.175620	4	0.304943	3	3	0.306649	0.119304	6.606557	0.262931
siControl Control UR vs. siGJA1 Control UR	11.92333	9.640000	0.487186	4	0.651620	3	3	6.112163	5.342200	1.309030	0.866165
siControl Control LL vs. siGJA1 Control LL	84.66333	87.38333	-0.582273	4	0.591625	3	3	5.905204	5.531097	1.139849	0.934645
siControl Control LR vs. siGJA1 Control LR	2.683333	2.470000	0.568042	4	0.600375	3	3	0.296704	0.578878	3.806513	0.416102
siControl 1 nM UL vs. siGJA1 1 nM UL	0.740000	0.540000	1.835970	4	0.140253	3	3	0.160935	0.098489	2.670103	0.544944
siControl 1 nM UR vs. siGJA1 1 nM UR	10.99000	9.563333	0.281447	4	0.792327	3	3	7.699435	4.219483	3.329656	0.461931
siControl 1 nM LL vs. siGJA1 1 nM LL	85.46667	86.89000	-0.269406	4	0.800943	3	3	8.303983	3.844620	4.665156	0.353035
siControl 1 nM LR vs. siGJA1 1 nM LR	2.806667	3.003333	-0.443860	4	0.680097	3	3	0.641275	0.421584	2.313766	0.603543
siControl 3 nM UL vs. siGJA1 3 nM UL	0.673333	0.693333	-0.121816	4	0.908919	3	3	0.273922	0.076376	12.86286	0.144270
siControl 3 nM UR vs. siGJA1 3 nM UR	12.26000	9.463333	0.514882	4	0.633780	3	3	8.177671	4.651294	3.091090	0.488867
siControl 3 nM LL vs. siGJA1 3 nM LL	83.10667	86.36667	-0.571287	4	0.598372	3	3	8.411167	5.190543	2.625953	0.551579
siControl 3 nM LR vs. siGJA1 3 nM LR	3.960000	3.476667	1.476827	4	0.213775	3	3	0.026458	0.566245	458.0476	0.004357

siControl 10 nM UL vs. siGJA1 10 nM UL	1.320000	0.780000	2.743793	4	0.051703	3	3	0.278388	0.196723	2.002584	0.666093
siControl 10 nM UR vs. siGJA1 10 nM UR	15.05333	10.52333	0.606646	4	0.576832	3	3	10.29012	7.835460	1.724693	0.734028
siControl 10 nM LL vs. siGJA1 10 nM LL	76.21333	83.04333	-0.784622	4	0.476538	3	3	11.81701	9.363794	1.592618	0.771421
siControl 10 nM LR vs. siGJA1 10 nM LR	7.413333	5.653333	1.337682	4	0.251991	3	3	1.367711	1.822809	1.776208	0.720407
siControl 33 nM UL vs. siGJA1 33 nM UL	3.133333	0.956667	6.998884	4	0.002193	3	3	0.522047	0.132791	15.45558	0.121539
siControl 33 nM UR vs. siGJA1 33 nM UR	20.52667	10.74667	1.311997	4	0.259749	3	3	12.14014	4.395024	7.630004	0.231750
siControl 33 nM LL vs. siGJA1 33 nM LL	59.68000	80.34000	-1.98360	4	0.118313	3	3	16.76879	6.651940	6.354875	0.271928
siControl 33 nM LR vs. siGJA1 33 nM LR	16.66667	7.956667	2.624750	4	0.058507	3	3	5.221057	2.403359	4.719322	0.349692

Table S24. continuation

siControl 100 nM UL vs. siGJA1 100 nM UL	6.433333	1.110000	3.780954	4	0.019420	3	3	2.402110	0.420357	32.65497	0.059427
siControl 100 nM UR vs. siGJA1 100 nM UR	27.58667	12.27000	1.452476	4	0.220018	3	3	17.34608	5.719974	9.196333	0.196149
siControl 100 nM LL vs. siGJA1 100 nM LL	45.18667	75.08000	-3.01530	4	0.039345	3	3	15.41021	7.575005	4.138579	0.389213
siControl 100 nM LR vs. siGJA1 100 nM LR	20.78667	11.54000	4.985388	4	0.007568	3	3	2.066938	2.459289	1.415676	0.827925

Table S25. Changes in the effect of paclitaxel on cell viability after transfection Cx43. FaDu was analyzed by trypan blue exclusion test after 48 h of treatment with paclitaxel at different concentrations. Statistical analysis was performed by Student's t-test, the IC_{50} values of the cell lines were compared to each other. Red color indicate if $p < 0.05$. (Figure 6)

Group 1 vs. Group 2	T-test for Independent Samples (plasmid+paclitaxel) Note: Variables were treated as independent samples										
	Mean Group 1	Mean Group 2	t-value	df	p	N 1	N 2	Std.Dev. Group 1	Std.Dev. Group 2	F-ratio Variances	p Variances
FaDu ctrl plasmid vs. FaDu Cx43 plasmid	63.2667	6.6186667	2.62890	4	0.05825	3	3	37.1378	3.7090195	100.256738	0.0197518

Table S26. Data tables summarizing the results of Cx43 and Bcl-2 immunohistochemical analysis.

**Primary tumor site**	**Bcl-2 protein expression**		**Total**
	**0**	**1**	
Oropharynx	15	5	20
Larynx	18	2	20
Hypopharynx	15	2	17
Oral cavity	1	0	1
**Total**	49	9	58
**Primary tumor site**	**Cx43 protein expression**		**Total**
	**0**	**1**	
Oropharynx	5	15	20

Larynx	6	14	20
Hypopharynx	3	14	17
Oral cavity	0	1	1
**Total**	14	44	58

**Grade**	**Cx43 protein expression**		**Total**
	**0**	**1**	
1	2	3	5
2	5	20	25
3	6	17	23
**Total**	13	40	53

**Grade**	**Bcl-2 protein expression**		**Total**
	**0**	**1**	
1	4	1	5
2	25	0	25
3	16	7	23
**Total**	45	8	53

**Stage**	**Bcl-2 protein expression**		**Total**
	**0**	**1**	
1	6	0	6
2	8	1	9
3	10	3	13
4a	19	4	23
4b	3	1	4
4c	3	0	3
**Total**	49	9	58

**Stage**	**Cx43 protein expression**		**Total**
	**0**	**1**	
1	1	5	6
2	1	8	9
3	3	10	13
4a	8	15	23
4b	1	3	4

4c	0	3	3
**Total**	14	44	58

Table S26. continuation

**TNM[1] T parameter**	**Cx43 protein expression**		**Total**
	**0**	**1**	
1	2	8	10
2	5	10	15
3	5	16	21
4a	2	8	10
4b	0	2	2
**Total**	14	44	58
**TNM[1] T parameter**	**Bcl-2 protein expression**		**Total**
	**0**	**1**	
1	10	0	10
2	12	3	15
3	15	6	21
4a	10	0	10
4b	2	0	2
**Total**	49	9	58
**Presence of lymph node metastasis**	**Bcl2 protein expression**		**Total**
	**0**	**1**	
yes	25	2	27
no	24	7	31
**Total**	49	9	58
**Presence of lymph node metastasis**	**Cx43 protein expression**		**Total**
	**0**	**1**	
yes	5	22	27
no	9	22	31
**Total**	14	44	58

**Presence of lymph node metastasis**	**Cx43 and Bcl-2 protein expression**				**Total**
	**Bcl-2 0 Cx43 0**	**Bcl-2 0 Cx43 1**	**Bcl-2 1 Cx43 0**	**Bcl-2 1 Cx43 1**	

yes	4	21	1	1	27
no	4	20	5	2	31
**Total**	8	41	6	3	58
**Primary tumor site**	**Cx43 and Bcl-2 protein expression**				**Total**
	**Bcl-2 0 Cx43 0**	**Bcl-2 0 Cx43 1**	**Bcl-2 1 Cx43 0**	**Bcl-2 1 Cx43 1**	
Oropharynx	2	13	3	2	20
Larynx	4	14	2	0	20
Hypopharynx	2	13	1	1	17
Oral cavity	0	1	0	0	1
**Total**	8	41	6	3	58

[1] TNM: tumor, node and metastasis, UICC TNM 7th edition

Table S26. continuation

**Stage**	**Cx43 and Bcl-2 protein expression**				**Total**
	**Bcl-2 0 Cx43 0**	**Bcl-2 0 Cx43 1**	**Bcl-2 1 Cx43 0**	**Bcl-2 1 Cx43 1**	
1	1	5	0	0	6
2	1	7	0	1	9
3	1	9	2	1	13
4a	5	14	3	1	23
4b	0	3	1	0	4
4c	0	3	0	0	3
**Total**	8	41	6	3	58
**Grade**	**Cx43 and Bcl-2 protein expression**				**Total**
	**Bcl-2 0 Cx43 0**	**Bcl-2 0 Cx43 1**	**Bcl-2 1 Cx43 0**	**Bcl-2 1 Cx43 1**	
1	1	3	1	0	5
2	5	20	0	0	25
3	2	14	4	3	23
**Total**	8	37	5	3	53

© 2019 by the authors. Submitted for possible open access publication under the terms and conditions of the Creative Commons Attribution (CC BY) license (http://creativecommons.org/licenses/by/4.0/).
